# Nature-Inspired Biphenyls and Diphenyl Ethers: Design, Synthesis, and Biological Evaluation

**DOI:** 10.1021/acsomega.5c02099

**Published:** 2025-05-16

**Authors:** Francesca Sacchi, Sharmila Ghosh, Sabrina Dallavalle, Dimitrios Fessas, Paolo Cortesi, Piera Anna Martino, José Luis Ermini Starna, Andrea Pinto, Francesca Annunziata, Salvatore Princiotto, Andrea Kunova

**Affiliations:** † Department of Food, Environmental and Nutritional Sciences (DeFENS), 9304 University of Milan, Via Celoria 2, 20133 Milan, Italy; ‡ Department of Biomedical, Surgical and Dental Sciences (DSBCO), One Health Unit, University of Milan, Via Pascal 36, 20133 Milan, Italy

## Abstract

Phlorotannins are polyphenolic compounds made of phloroglucinol units mainly found in brown algae, exhibiting diverse structural features and bioactive properties. Notably, dimeric phlorotannins, i.e., fucols and phloroethols, share the biphenyl and diphenyl ether motifs characteristic of several antimicrobial phytoalexins, typically produced by plants under biotic and abiotic stress conditions. Considering the difficult supply from their biological matrices, natural difucol, hexaacetyl-difucol, and diphloroethol have been synthesized; moreover, a small collection of analogues has been prepared by versatile synthetic approaches consisting in a partial or complete methylation (or acetylation) of the monomers. Finally, oxidative dimerization or Ullmann condensation provided the desired compounds. The resulting derivatives have been evaluated as inhibitors of mycelium growth, spore germination, and appressorium formation of Pyricularia oryzae (PO-2107 Qol-resistant strain and PO-A252 Qol-sensitive strain), Botrytis cinerea (BC-2A10), and Fusarium culmorum (FC-UK). None of the biphenyl derivatives significantly affected the tested fungal strains; however, polymethylated diphenyl ethers **10**, **11**, and **14** at 500 μM concentration showed inhibition of mycelium growth between 20 and 45% against all the tested strains, highlighting that the methylation pattern, as well as the connection between the two aromatic rings, could have a role in the interaction with the biological target. Antibacterial assays against one Gram-positive (Staphylococcus aureus) and three Gram-negative bacteria (Escherichia coli, Salmonella enterica Enteritidis, and Pseudomonas aeruginosa) showed minimum inhibitory concentrations (MICs) equal or higher than 128 μg/mL for all the tested compounds.

## Graphical abstract

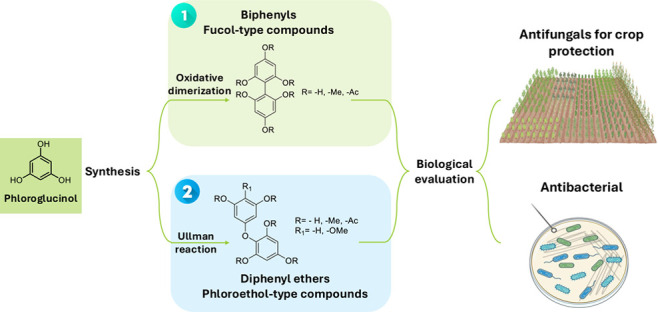

## Introduction

Crop protection represents an absolute priority to ensure food safety, especially for poor countries, with fungal infections considered among the most dangerous threats to agriculture and primary industry.^1–3^ Hence, there is an urgent need for effective treatments that can prevent and limit the development of plant diseases and subsequent food loss. During the last years, biphenyls and diphenyl ethers from natural sources have gained interest for their enormous potential as antimicrobial agents, showing very promising activities and raising interest toward these classes of molecules. For instance, antifungal biphenyl lignans magnolol and honokiol and phytoalexin noraucuparin from Sorbus aucuparia (family Rosaceae) have been widely studied and used as a source of inspiration for the preparation of bioactive derivatives.^4–7^ In particular, biphenyls derived from species belonging to the Malinae subtribe have gained momentum for their biological properties: to date, 46 biphenyls have been isolated from 44 different species, with half of them identified as phytoalexins able to inhibit fungal growth.^8,9^ In the same way, interesting inhibition potential toward invasive species (e.g., bacteria, fungi, and viruses) was shown by diphenyl ethers found in endophytic fungus Arthrinium arundinis isolated from tobacco leaves and abundantly occurring in marine plants and microorganisms.^10–12^

Phlorotannins are oligomers of phloroglucinol characterized by biphenyl and diphenyl ether backbones, which are present in macroalgae. Similarly to other plants, marine algae are capable to generate phytoalexins as bioactive compounds for self-protection from external factors, such as UV radiation, physical damages, as well as other abiotic and biotic stress conditions.^13^ In particular, phlorotannins, similarly to tannins and flavonols in higher plants, have multifunctional roles as essential components in the polymerization of the cell wall (insoluble cell wall-bound fraction) and are powerful antioxidants, thanks to their high degree of hydroxylation.^14^ Phlorotannins are present in a wide range of molecular sizes (126 Da–650 kDa) and essentially consist of a biphenyl or diphenyl ether skeleton, depending on the bond between the units of phloroglucinol. For example, phloroethols show diaryl ether portions, while fucols are characterized by the presence of a C–C bond between two aryl structures (Figure 1).^13,15^

**Figure 1:**
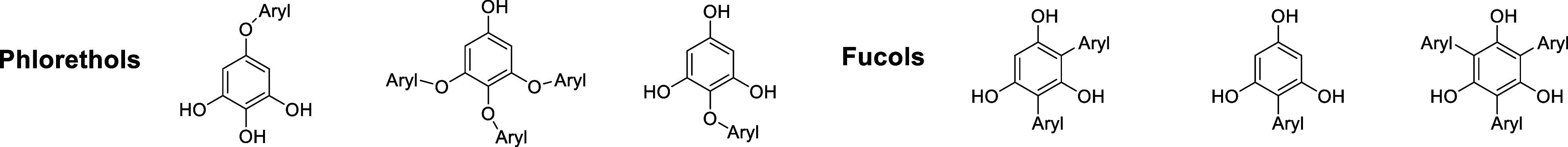
Basic chemical structure of phloroethols and fucols.

De Corato and colleagues tested the antifungal activity of brown seaweed extracts against phytopathogens (Botrytis cinerea, Monilinia laxa, and Penicillium digitatum). The results showed that phloroethols and fucophloroethols contained in Laminaria digitata extracts are effective against B. cinerea and M. laxa (100% mycelial growth inhibition), at 30 g/L concentration.^16,17^ Several papers described phlorotannins also as antibacterial agents;^18–20^ however, biological evaluation is typically carried out on enriched fractions rather than on pure isolated compounds. Nonetheless, direct isolation of phlorotannins from their natural sources is quite impractical because of the low amount and the presence of multiple structural and conformational isomers.^21^ In addition to the great interest in these specialized metabolites, during the last 50 years, only a few derivatives of natural phlorotannins have been chemically prepared.^22–24^ Therefore, the development of versatile synthetic methodologies to afford highly pure natural and nature-derived phlorotannins is a valid strategy to deal with current challenges in extraction procedures and to perform structure–activity relationship (SAR) studies.

In this context, to investigate the potential application of natural biphenyl and diphenyl ether derivatives as antimicrobial agents, a series of natural and nature-derived phloroethols and fucols with diverse methylation/acetylation patterns were designed and synthesized following two different synthetic approaches (Figure 2). Their potential as fungicides has been evaluated against four strains of phytopathogenic fungi: Pyricularia oryzae (PO-2107 QoI-resistant strain and PO-A252 QoI-sensitive strain), B. cinerea (BC-2A10), and Fusarium culmorum (FC-UK). In addition to the inhibition of mycelium growth, the effect on spore germination and appressorium formation was evaluated as well. Moreover, minimum inhibitory concentrations have been investigated against one Gram-positive (Staphylococcus aureus) and three Gram-negative bacteria (Escherichia coli, Salmonella enterica Enteritidis, and Pseudomonas aeruginosa).

**Figure 2:**
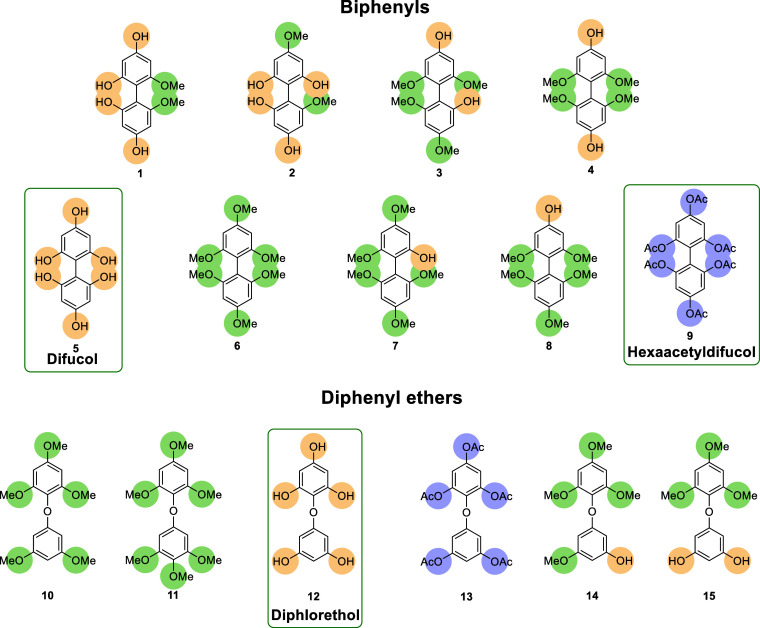
Chemical structures of the synthesized biphenyls and diphenyl ethers.

## Results and Discussion

### Synthesis

Oxidative dimerization of pure phloroglucinol mediated by FeCl_3_ was first attempted (Scheme 1). Due to the high reactivity of the radical species involved, including the phenolic units on the coupling products, complex mixtures of oligomers (i.e., dimers, trimers, tetramers, and so on) were generated by exploiting this approach. To facilitate the purification step, the obtained mixture was acetylated as described by. Isaza Martínez and Torres Castañeda.^25^ Also, in this case, the purification was troublesome, giving only hexa-acetyldifucol **9** as a pure compound in a very low yield. Consequently, fucol-type dimers were synthesized starting from partially protected phloroglucinol units following the procedure reported by Vershinin et al. with minor modifications.^26^ This approach allowed the obtainment of a small collection of methylated fucols in sufficient quantities to perform SAR studies.

**Scheme 1:**
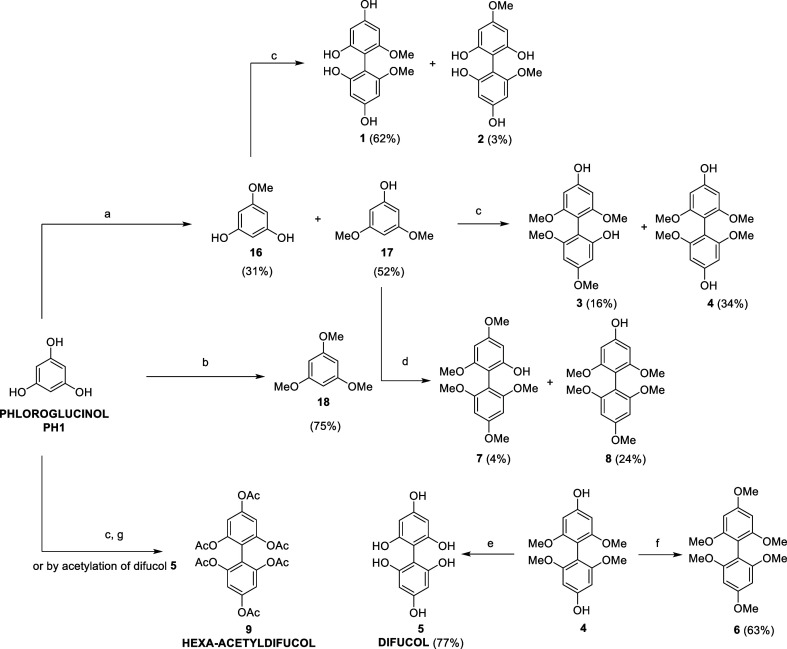
(a) Me_2_SO_4_ (0.3 equiv), K_2_CO_3_, Acetone, 55 °C, 24 h; (b) Me_2_SO_4_ (3 equiv), K_2_CO_3_, Acetone, 55 °C, 3 h; (c) FeCl_3_·6H_2_O (2 equiv), MeOH/H_2_O, r.t., 24–48 h; (d) FeCl_3_·6H_2_O (0.15 equiv), (*t*-BuO)_2_O, HFIP, r.t., 6 h; (e) BBr_3_, DCM, N_2_, −78 °C to r.t., 18 h; (f) Me_2_SO_4_, K_2_CO_3_, Acetone, r.t., 21 h; and (g) Ac_2_O, Pyridine, r.t., 24 h

Partial methylation of phloroglucinol was obtained by adjusting equivalents of dimethyl sulfate and reaction times. Oxidative dimerization of **16**, **17**, and **18** generated derivatives **1**–**4** and **7**–**8** (Scheme 1). In particular, compounds **1**–**4** were synthesized using iron trichloride in a stoichiometric amount in a methanol/water solution, whereas compounds **7** and **8** were prepared using catalytic iron trichloride and Luperox P as the radical initiator and hexafluoroisopropanol (HFIP) as the solvent. Complete deprotection of **4** gave natural difucol **5**; conversely, methylation of **4** with dimethyl sulfate gave permethylated difucol derivative **6**.

On the other hand, diphenyl ethers were not achievable through oxidative dimerization, therefore a different approach was designed. Phloroethols were obtained using the reaction between phenol and aryl bromide as a key step to form an ether bond (Scheme 2).

**Scheme 2:**
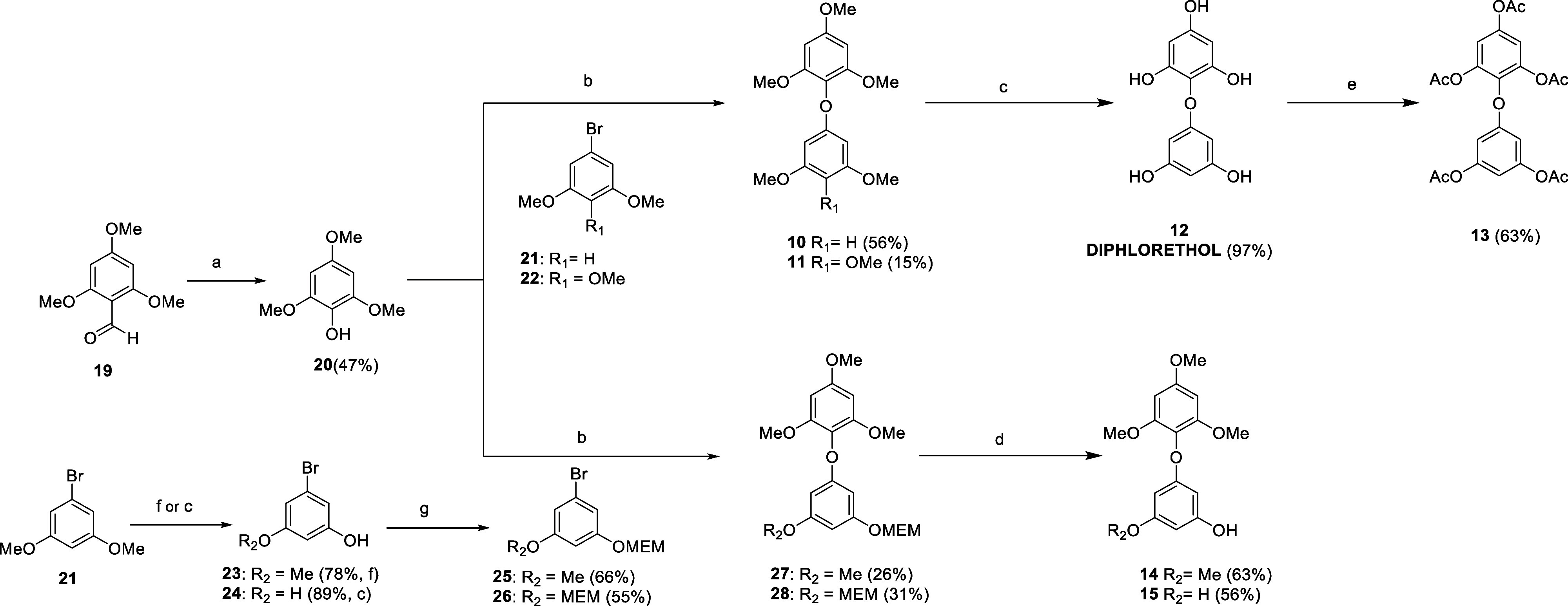
(a) (1) *m*CPBA, DCM, N_2_, 0 °C to r.t, 4 h; (2) KOH, MeOH, 0 °C, 45 min; (b) CuI, Picolinic Acid, K_2_CO_3_, DMSO, 130 °C, 5 d; (c) BBr_3_, DCM, N_2_, −78 °C to r.t., 16 h; (d) HCl, MeOH, r.t., 16 h; (e) Ac_2_O, DMAP, TEA, THF, r.t., 7 h; (f) 1-Dodecanethiol, NaOH, NMP, 130 °C, 1 h; and (g) MEM-Cl, DIPEA, THF, N_2_, 0 °C to r.t., 16 h

First, phenol **20** was prepared by a Baeyer–Villiger oxidation of commercially available aldehyde **19**. The synthesis of permethylated **10**, from phenol **20** and aryl bromide **21**, was attempted following the procedure reported by Paizs et al., giving a low yield (14%).^27^ Multiple reaction conditions were investigated, which eventually led to the choice of copper salt (CuI) as catalyst, picolinic acid as ligand, and DMSO as solvent, yielding **10** in an acceptable 56% yield (130 °C for 5 days). Analogously, starting from **22**, compound **11** was obtained. Deprotection of **10** with boron tribromide led to natural diphloroethol **12**, which was reacted with acetic anhydride to get peracetylated derivative **13**.

In parallel, deprotection of **21** led to phenols **23** and **24**, that were reacted with MEM-Cl to give intermediates **25** and **26**. Compounds **27** and **28** were synthesized by the Ullman reaction between phenol **20** and aryl bromide **25** and **26**, respectively. Selective MEM-deprotection of **27** and **28** with hydrochloric acid gave derivatives **14** and **15**.

### Antifungal Activity

The panel of 15 biphenyls and diphenyl ethers as well as the monomer phloroglucinol (**PH1**) were tested to evaluate their antifungal activity against four different strains of phytopathogenic fungi, P. oryzae (PO21_07 QoI-resistant strain and PO_A252 QoI-sensitive strain), B. cinerea (BC-2A10), and F. culmorum (FC-UK). First, the biological activity of the compounds was assessed in terms of the inhibition of mycelium growth. The monomer **PH1** and the fucol-type dimers showed only low or no inhibition toward fungal mycelium, regardless the presence of free hydroxyl groups or different degrees of methylation patterns (Figure 3). On the other hand, phloroethols showed more promising activity, in particular toward the two strains of P. oryzae (compounds **10** and **14**) and B. cinerea (compound **11**). These results indicate that the connection between the monomers is crucial for the activity, since the C–C bond typical of fucols seems to be detrimental for the antifungal properties. Polymethylated diphenyl ethers resulted the most promising compounds, suggesting that increasing lipophilicity could have some impact on bioavailability and biological target interaction. Lack of antifungal activity was indeed observed for compounds with *c*Log *P* lower than 1 (compounds **1**, **3**, **4**, **5**, **9**, **12**, and **13**, as well as phloroglucinol) (Table S1). On the other hand, increased lipophilicity was related to an inhibitory activity toward the assayed fungi. No clear correlation was highlighted between predicted Log *P* and the biological effect for intermediate values.

**Figure 3:**
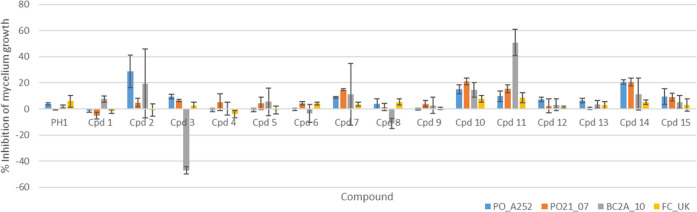
Inhibition of mycelium growth of P. oryzae (PO_A252, PO21_07), B. cinerea (BC2A_10), and F. culmorum (FC_UK) by phloroglucinol (**PH1**), biphenyls (compounds **1**–**9**), and diphenyl ethers (compounds **10**–**15**).

Considering these preliminary results, the promising compounds **10**, **11**, and **14** were tested also at 500 μM (Figure 4). The results show a dose-dependent response, and the highest inhibitory activity was observed against B. cinerea.

**Figure 4:**
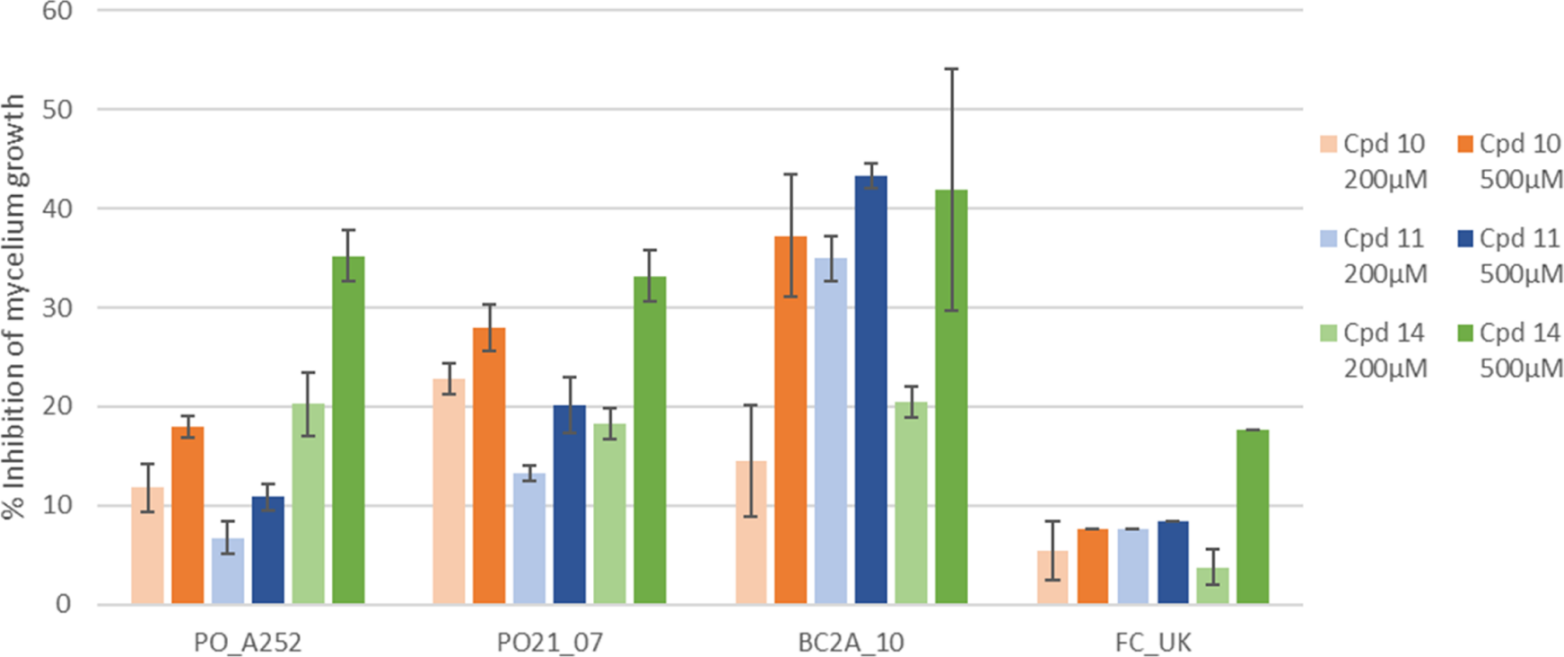
Inhibition of the mycelium growth of P. oryzae (PO_A252, PO21_07), B. cinerea (BC2A_10), and F. culmorum (FC_UK) by compounds **10**, **11**, and **14** at 200 and 500 μM concentration. The error bars represent the standard deviation.

To better investigate the potential application as antifungals of these compounds against P. oryzae and B. cinerea, they were further assessed in terms of the inhibition of spore germination. In P. oryzae, the germinating conidium develops at the end of the germ tube, a specialized dome-shaped infection structure called “appressorium”, necessary for the penetration of the rice cuticle.^28,29^ Therefore, in P. oryzae, also inhibition of appressorium formation by the compounds was tested.

However, none of the compounds inhibited the germination of either P. oryzae or B. cinerea, showing no or very low (>10%) inhibition of spore germination (Figure S23). The only exception was compound **15**, which inhibited the appressorium formation of P. oryzae by 48% without affecting the germination.

### Antibacterial Activity

For a complete biological characterization of the synthesized compounds, antimicrobial properties were evaluated using the Minimum Inhibitory Concentration (MIC) test against representative and well-characterized Gram-positive (S. aureus) and Gram-negative bacteria (E. coli, S. enterica Enteritidis, and P. aeruginosa). Nevertheless, the MIC’s values ranged between 256 and 128 μg/mL toward the selected microorganisms (Table S2), high concentrations compared to values normally indicating efficacy of the antimicrobial molecules. However, a low efficacy toward the bacteria and no differences between fucols and phloroethols were observed ascribable to the bond between monomers (C–C or C–O–C bonds).

## Conclusions

Biphenyls and diphenyl ethers are widely described natural structural motifs with antimicrobial potential. Among the others, naturally occurring phlorotannins consist of both structural units (known as fucols and phloroethols); however, difficult isolation processes from their natural matrices make synthetic efforts necessary for a proper biological evaluation. Natural and nature-derived biphenyls **1**–**9** and diphenyl ethers **10**–**15** were prepared and tested as antifungal and antibacterial agents. Notably, none of the natural compounds were able to completely inhibit fungal growth at the tested concentrations, while diphenyl ethers **10**, **11**, and **14** showed significant inhibition of the mycelium growth of P. oryzae and B. cinerea, with lower activity observed against F. culmorum. The most promising compounds were all characterized by the presence of a (poly)­methylated diphenyl ether nucleus. Indeed, the methylation pattern together with the C–O–C fragment seemed to heavily affect the interaction with the biological target. Conversely, no antibacterial effect was detected. Further investigations are needed to deepen the structure–activity relationship to enhance the antifungal activity, and the key structural features required by these scaffolds to act as antibacterial agents.

## Experimental Section

All reagents and solvents were purchased from commercial suppliers and used without further purification. The ^1^H NMR and ^13^C NMR spectra were recorded with a Bruker AVANCE Neo 400 MHz spectrometer. Chemical shifts (δ) are expressed in ppm, and coupling constants (*J*) are in Hertz. All reactions requiring anhydrous conditions were performed under a positive nitrogen flow, and all glassware was oven-dried. Isolation and purification of the compounds were performed by flash column chromatography on silica gel 60 (230–400 mesh) through isocratic or gradient elution with different ratios of the cyclohexane/ethyl acetate mixture and Büchi Pump Manager (C-615 and C-601) equipment. Thin layer chromatography (TLC) analyses were performed by using commercial silica gel 60 F254 aluminum sheets; spots were further evidenced by spraying with an acidic solution of *p*-anisaldehyde in EtOH. *c*Log *P* values have been calculated by ChemDraw 23.0.1 exploiting the algorithm licensed by BioByte Corp. Mass spectrometry analyses were performed at the Mass Spectrometry facility of Unitech COSPECT at the University of Milan (Italy).

### General Procedure for Phloroglucinol Methylation

To a solution of phloroglucinol (2.0 g, 15.8 mmol, 1 equiv, 0.5 M) was added K_2_CO_3_ (2.2 g, 15.0 mmol, 1 equiv). Me_2_SO_4_ was added dropwise (0.33 equiv for the synthesis of compounds **16** and **17**, 3 equiv for the synthesis of compound **18**). The reaction mixture was left stirring under reflux (24 h for the synthesis of **26** and **27**, 3 h for the synthesis of **18**). Acetone was removed under pressure; the crude was dissolved in water (40 mL) and HCl 1 N was added until pH 1. The mixture was extracted with DCM (50 mL × 4), and the organic layers were washed with brine, dried over anhydrous Na_2_SO_4_, and concentrated under reduced pressure. The residue was purified by flash chromatography (cyclohexane/EtOAc gradient 8:2 to 0:100) to afford the desired products as a beige solid.

### General Procedure for the Oxidative Dimerization in MeOH–H_2_O

To a solution of phenol (1 equiv) in MeOH was added dropwise a solution of FeCl_3_·6H_2_O (2 equiv) in water. The mixture was stirred at room temperature (24 h–48 h). MeOH was evaporated and the residue was diluted with water and extracted with EtOAc. The combined organic layers were dried over anhydrous Na_2_SO_4_ and concentrated under reduced pressure. The crude product was purified by flash chromatography.

### General Procedure for Oxidative Dimerization in HFIP

To a solution of 3,5-dimethoxyphenol (1 equiv), 1,3,5-trimethoxybenzene (2.1 equiv), and FeCl_3_·6H_2_O (0.15 equiv) in HFIP 0.5 M (1.3 mL), di-*t*-butylperoxide (2.1 equiv) was added dropwise. The mixture was stirred at room temperature for 6 h. The volatiles were removed under reduced pressure, and the crude residue was purified by flash chromatography.

### General Procedure for MEM Protection of Phenols

DIPEA (2 equiv for each –OH group) was added to a solution of phenol (1 equiv) in dry THF (0.3 M) under a nitrogen atmosphere and was stirred at 0 °C for 10 min. MEM-Cl (2 equiv for each –OH group) was added to the reaction mixture, which was stirred at room temperature overnight and then acidified with 1 M HCl. The aqueous layer was extracted with ethyl acetate, the collected organic phases were washed with brine, dried over Na_2_SO_4_, and filtered, and the solvent was evaporated under reduced pressure. The crude products were purified by flash column chromatography as described below.

### General Procedure for Ullman Reaction

Solid reagents were placed in a pyrex screw cap tube, equipped with a stirring bar, in the following order: CuI (0.11 equiv), picolinic acid (0.21 equiv), phenol (1.07 equiv), aryl-bromide (1.00 equiv), and K_2_PO_4_ (1.96 equiv). Finally, dry DMSO (0.3 M) was added in the tube and stirred in a closed atmosphere at 110 °C for 5 days, then the reaction mixture was cooled at room temperature and diluted with water and ethyl acetate. The aqueous layer was extracted with ethyl acetate, the collected organic phases were washed with brine, dried over Na_2_SO_4_, and filtered, and the solvent was evaporated under reduced pressure. The crude products were purified by flash column chromatography as described below.

### General Procedure for MEM Deprotection

Concentrated HCl (36%, 3 equiv for each –OMEM group) was added to a solution of MEM-protected intermediate (1 equiv) in MeOH (0.1 M) and was stirred at room temperature overnight. The solvent was concentrated in vacuum, then water was added, and the aqueous phase was extracted with ethyl acetate. The collected organic phases were washed with brine, dried over Na_2_SO_4_, and filtered, and the solvent was evaporated under reduced pressure. The crude products were purified by flash column chromatography, as described below.

### General Procedure for Methoxy-Deprotection

1 M BBr_3_ solution in DCM (2 equiv for each –OMe group) was added to a solution of the permethylated derivative (1 equiv) in dry DCM (0.12 M) under a nitrogen atmosphere at −78 °C. The reaction was stirred at the same temperature for 1 h and then at room temperature overnight. The reaction was cooled at 0 °C and then quenched with water, then the organic solvent was evaporated under reduced pressure. The remaining aqueous phase was extracted with ethyl acetate; the collected organic phases were washed with brine, dried over Na_2_SO_4_, and filtered, and the solvent was evaporated under reduced pressure. The crude products were purified by flash column chromatography as described below.

### Antifungal Activity of the Compounds

#### Fungal Strains

In this study, four strains belonging to three different fungal species were used: P. oryzae strain sensitive to quinone outside inhibitor (QoI) fungicides A252 and resistant to QoI PO21_07, F. culmorum Fc-UK (NRRL54111),^30^ and B. cinerea BC_2A. The strains belong to a vast collection of monoconidial isolates maintained at the Laboratory of Plant Pathology, University of Milan. The strains were maintained as single-spore isolates on a malt-agar medium (MA: 20 g/L malt extract, Oxoid, U.K.; 15 g/L agar, VWR Life Science, U.S.A.) at 4 °C.

#### Inhibition of Mycelium Growth

The mycelium inhibition of fungal strains by the tested compounds was evaluated as previously described.^31^ Briefly, a 0.5 cm mycelium plug obtained from actively growing fungal colonies was transferred to MA medium plates supplemented or not with tested compounds in three biological replicates. The compounds were tested at a concentration 200 μM, and compounds **10**, **11**, and **14** were tested also at a concentration of 500 μM. Due to a low solubility of the tested molecules in water, they were dissolved in DMSO. Therefore, multiple controls were included: MA medium without any supplement (MA) and MA medium supplemented with DMSO at a final concentration of 1% v/v. The plates were incubated at 24 °C in the dark. The mycelium growth was measured 3 days after inoculation (DAI) for B. cinerea and F. culmorum and 7 DAI for P. oryzae. The inhibition of mycelium growth (%) was calculated by comparing the mycelium growth on solvent-containing medium and compound-supplemented plates (Tables S3–S6).

#### P. oryzae Spore Germination and Appressorium Inhibition

P. oryzae PO21_02 and PO21_O7 were inoculated on an MA medium and incubated in a growth chamber at 24 °C in the dark for 12–14 days. Then, 2 mL of sterile water was added to the mycelium and the formed conidia were scraped from the whole mycelium surface with the help of the glass spatula. The spore suspension was collected and filtered through two layers of sterile gauze into an Eppendorf tube to remove the mycelium. The spore concentration was estimated using a Thoma hemocytometer and adjusted to a concentration of 2 × 10^4^ conidia/mL.

The tested compounds were dissolved in DMSO and were added to 100 μL of conidial suspension to obtain a final concentration of 200 μM and 1% DMSO. Conidia treated with 1% DMSO were considered a control.

20 μL of the conidial suspension in three replicates was applied on a microscopic cover slide in a wet chamber and incubated for 24 h at 24 °C in the dark. The germination of 100 randomly chosen conidia for each treatment and replica was determined. The conidia were assigned to germination classes: NG = nongerminated, G = germinated, and A = germinated with appressorium.

#### B. cinerea Spore Germination and Germ Tube Elongation

B. cinerea BC_2A was inoculated on a Czapek-Dox Yeast medium (CZY; 35 g/L Difco Czapek-Dox broth, BD, France; 2 g/L Difco yeast extract, Oxoid, U.K.; 15 g/L agar, VWR Life Science, U.S.A.) and incubated in the growth chamber at 24 °C in the dark for 7–10 days. The spores were collected as described for P. oryzae and the concentration was adjusted to 1 × 10^4^ conidia/mL and were then diluted in 20% Potato dextrose broth (PDB; Difco Potato dextrose broth, BD, France).

The compounds were added to the spore suspension as described before. 20 μL of conidial suspension in three replicates was applied on a microscopic cover slide in a wet chamber and incubated for 16 h at 15 °C in the dark. The germination of 100 randomly chosen conidia for each treatment and replica was determined.

#### Inhibition Percentage Calculations

The inhibition percentage of mycelium growth was calculated as *I* % = (*C* – *T*)/*C* × 100, where *C* = mycelium growth in the solvent medium and *T* = mycelium growth in the medium added to the tested compound.

For P. oryzae spore germination, the percentage of spore germination was calculated according to the formula: Germ (%) = Σ­(*G* + *A* + ANM)/*n* × 100, where *n* = total number of spores observed per replica. For B. cinerea, only germinated (*G*) and not germinated (NG) categories were assessed; therefore, the percentage of spore germination was calculated according to the formula: Germ (%) = *G*/*n* × 100. The inhibition of spore germination was calculated as IG (%) = (GermC – GermT)/GermC × 100, where GermC was % germination in the control (solvent), and GermT was % germination in the treated sample.

Inhibition of appressoria formation was calculated as IA (%) = (GermT – AT)/GermT × 100, where AT was the percentage of spores with appressoria in the treated sample.

### Statistical Analysis

The statistical analyses were performed using R software, version 4.4.0,^32^ in R Studio, version 2024.09.0.375.^33^ The percentage data of mycelium growth inhibition and spore germination, as well as P. oryzae appressorium formation, were square root arcsine transformed and submitted to ANOVA, followed by Tukey’s post hoc test for multiple comparison (*P* = 0.05), using the TukeyC package.^34^

### Minimum Inhibitory Concentration Test

The Minimum Inhibitory Concentration (MIC) was determined using the microdilution assay according to the Clinical and Laboratory Standards Institute (CLSI) guidelines (Clinical and Laboratory Standards Institute (CLSI, 2018) Performance Standards for Antimicrobial Susceptibility Testing. CLSI Approved Standard M100-S15. Clinical and Laboratory Standards Institute, Wayne). The evaluation of the antibacterial activity of the compounds (dissolved in DMSO) was performed using E. coli ATCC 25922 (Ec), S. enterica subsp. enterica ser. Enteritidis ISM 8324 (Se), P. aeruginosa IMV 1 (Pa), and S. aureus ATCC 6538 (Sa).

Stocks of the previously identified bacteria were thawed, and then they were streaked onto blood agar plates (Tryptic Soy Agar + 5% sheep blood [Microbiol, Italy]) and incubated at 37 °C for 24 h under aerobic conditions to obtain isolated and pure bacterial colonies. Then, all the bacteria were grown on Tryptic Soy Agar (TSA, Oxoid, Milan, Italy) and 3 or 4 isolated colonies (depending on the size of the colonies) were suspended in a sterile saline solution (9 g/L NaCl) to reach an initial concentration of 1.5 × 10^8^ CFU/mL (equivalent to 0.5 MacFarland standard). One hundred microliters of the 1:100 diluted cell suspensions were dispensed into each well of a 96-well microtiter plate. The strains were exposed to a 2-fold dilution series of each derivative (dissolved in DMSO). After incubation at 37 °C aerobically for 24 h, the MICs were determined as the lowest dilution of molecules able to inhibit visible bacterial growth. Positive and negative controls were tested for each plate. Assays were performed in triplicate.

## Supplementary Material



## Notes

Experimental procedures, product characterization, and biological evaluation data (PDF)
