# Supplementary material for: Nature-Inspired Biphenyls and Diphenyl Ethers: Design, Synthesis, and Biological Evaluation
Source: ACS Omega. 2025 May 16;10(21):22028–35. doi: 10.1021/acsomega.5c02099 (PMC12138666; doi:10.1021/acsomega.5c02099)
Supplement: Supplementary file 1 [file ao5c02099_si_001.pdf]

SUPPORTING INFORMATION

Nature inspired biphenyls and diphenyl ethers: design, synthesis and biological evaluation

Francesca Sacchi,[a,‡] Sharmila Ghosh,[a,‡] Sabrina Dallavalle,[a] Dimitrios Fessas,[a] Paolo Cortesi,[a] Piera Anna Martino,[b] José Luis Ermini Starna,[a] Andrea Pinto,[a] Francesca Annunziata,[a,*] Salvatore Princiotto,[a,*] Andrea Kunova[a]

[a] *Department of Food, Environmental and Nutritional sciences, Department of Food, Environmental and Nutritional Sciences (DeFENS), University of Milan, Via Celoria 2, 20133, Milan, Italy*
[b] *Department of Biomedical, Surgical and Dental Sciences, One Health Unit, University of Milan, via Pascal 36, 20133, Milan, Italy*

TABLE OF CONTENTS

General procedure for synthesis of compounds **1** and **2**……….……………………………………..S2

General procedure for compounds **3** and **4**……………………………………………………S3

Experimental procedure for the synthesis of compound **6**…………………………………….……S3

General procedure for synthesis of compounds **7** and **8**……………………………………….……S4

Experimental procedure for the synthesis of compound **9** ……………………………………………S5

Experimental procedure for the synthesis of **20**…………………………………………………S5

General Procedure for Ullman reaction ……………………...…………………………..S6

Experimental procedure for the synthesis of **23**…………………………………………………S8

General Procedure for MEM-protection of phenols………………………………………..S8

General Procedure for MEM-deprotection..…………………………………………………...S9

General Procedure for methoxy-deprotection…………………………………………….S10

Experimental procedure for the synthesis of **13**……………………………………………..S12

General procedure for **16**, **17** and **18**……………………………………………………S13

Figure S1-S22 (NMR spectra)..……………………………………………………...……S14

Figure S23…...………………………………………………………………………….....S35

Table S1-S6..……………………………………………………………………………...S36

General procedure for synthesis of compounds 1 and 2

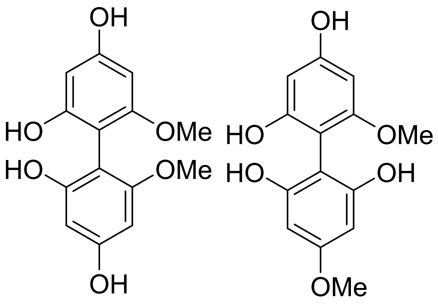

1 **2**

To a solution of 5-methoxybenzene-1,3-diol **16** (367 mg, 2.6 mmol, 1 eq) in MeOH (5 mL) a solution of $FeCl_3 \cdot 6H_2O$ (5.2 g, 5.2 mmol, 2 eq) in water (10 mL) was added dropwise. The mixture was stirred at room temperature for 48 h. MeOH was evaporated and the residue was diluted with water (20 mL) and extracted with EtOAc (4 × 40 mL). The combined organic layers were dried over anhydrous Na_2SO_4 and concentrated in vacuum. The crude residue was purified first by flash chromatography with a mixture of CyHex/EtOAc 4:6 and then with DCM/MeOH 95:5.

1: yellow solid; yield: 30%. R_f = 0.34 (DCM/MeOH 95:5). ^{1}H NMR (400 MHz, acetone-d_6) δ 6.08 (d, J = 2.2 Hz, 2H), 6.07 (d, J = 2.2 Hz, 2H), 3.61 (s, 6H). ^{13}C NMR (100 MHz, acetone-d_6) δ 160.0 (2C), 158.5 (2C), 157.0 (2C), 100.4 (2C), 95.5 (2C), 91.5 (2C), 54.8 (2C). HRMS (ESI) m/z calcd for $C_{14}H_{13}O_6$, $[M - H]^-$: 277.0712, found: 277.0713.

2: yellow solid; yield: 2%. R_f = 0.38 (DCM/MeOH 95:5). ^{1}H NMR (400 MHz, acetone-d_6) δ 6.11 (d, J = 2.2 Hz, 1H), 6.08 (d, J = 2.2 Hz, 1H), 6.05 (s, 2H), 3.73 (s, 3H), 3.64 (s, 3H). ^{13}C NMR (100 MHz, acetone-d_6) δ 160.8, 160.3, 159.1, 157.5, 157.2 (2C), 100.5, 98.9, 95.8, 93.3 (2C), 91.7, 54.9, 54.3. HRMS (ESI) m/z calcd for $C_{14}H_{13}O_6$, $[M - H]^-$: 277.0712, found: 277.0717.

General procedure for compounds 3 and 4

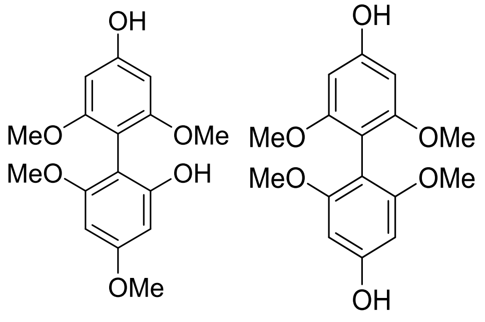

3 **4**

To a solution of 3,5-dimethoxyphenol **17** (1 g, 6.4 mmol, 1 eq) in MeOH (10 mL) was added dropwise a solution of $FeCl_3 \cdot 6H_2O$ (3.4 g, 12.8 mmol, 2 eq) in water (20 mL). The mixture was stirred at room temperature for 28 h. MeOH was evaporated and the residue was diluted with water (20 mL) and extracted with EtOAc (3 × 40 mL). The combined organic layers were dried over anhydrous Na_2SO_4 and concentrated under reduced pressure. The crude was purified by flash chromatography (CyHex/EtOAc gradient from 6:4 to 4:6)

3: beige solid; yield: 19%. R_f = 0.43 (CyHex/EtOAc 1:1). ^{1}H NMR (400 MHz, acetone-*d*$_6$) δ 8.27 (s, 1H), 6.72 (s, 1H), 6.18 (s, 2H), 6.11 (s, 2H), 3.77 (s, 3H), 3.63 (s, 6H), 3.62 (s, 3H). ^{13}C NMR (100 MHz acetone-*d*$_6$) δ 161.4, 160.8 (2C), 160.5, 159.7, 157.4, 104.3, 102.9, 94.3, 93.3 (2C), 91.5, 55.9 (3C), 55.4.

4: white solid; yield: 37%. R_f = 0.23 (CyHex/EtOAc 1:1). ^{1}H NMR (400 MHz, acetone-*d*$_6$) δ 8.11 (s, 2H), 6.16 (s, 4H), 3.59 (s, 12H). ^{13}C NMR (100 MHz, acetone-*d*$_6$) δ 159.4 (2C), 158.0 (4C), 104.4 (2C), 92.3 (4C), 55.0 (4C).

Experimental procedure for the synthesis of compound 6

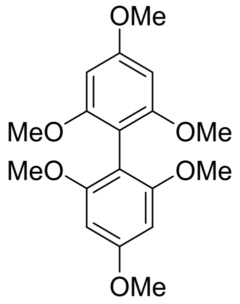

6

To a solution of 2,2',6,6'-tetramethoxy-[1,1'-biphenyl]-4,4'-diol **4** (300 mg, 0.94 mmol, 1 eq) in dry acetone (12 mL) K_2CO_3 (782 mg, 5.62 mmol, 6 eq) was added. The mixture was stirred at room temperature under nitrogen and Me_2SO_4 (700 µL, 11,24 mmol, 12 eq) was added dropwise. After 21 h, the mixture was filtered, and the solvent was evaporated under reduced pressure. The residue was dissolved in EtOAc (30 mL) and extracted with water (15 mL × 3). The organic phase was dried over anhydrous Na_2SO_4 and concentrated in

vacuum. The crude residue was purified by flash chromatography (CyHex/EtOAc 1:1) to afford the product as a white solid.

Yield: 63%. R_f = 0.79 (CyHex/EtOAc 1:1).^{1}H NMR (400 MHz, acetone-d_6) δ 6.25 (s, 4H), 3.84 (s, 6H), 3.65 (s, 12H). ^{13}C NMR (100 MHz, acetone-d_6) δ 160.7 (2C), 159.3 (4C), 105.4 (2C), 91.0 (4C), 55.1 (4C), 54.6 (2C).

General procedure for synthesis of compounds 7 and 8

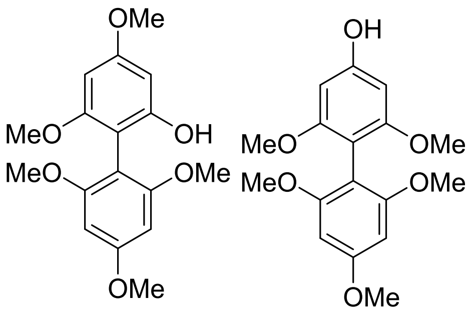

7 **8**

To a solution of 3,5-dimethoxyphenol **17** (100 mg, 0.65 mmol, 1 eq), 1,3,5-trimethoxybenzene (229 mg, 1.36 mmol, 2.1 eq) and $FeCl_3 \cdot 6H_2O$ (26 mg, 0.11 mmol, 0.15 eq) in HFIP 0.5 M (1.3 mL), di-*t*-butylperoxide (250 µL, 2.1 eq) was added dropwise. The mixture was stirred at room temperature for 6 h. The volatiles were removed under reduced pressure and the crude residue was purified by flash chromatography (CyHex/EtOAc gradient from 6:4 to 1:1)

7: beige solid; yield: 4%. R_f = 0.40 (CyHex/EtOAc 6:4). ^{1}H NMR (400 MHz, acetone-d_6) δ 6.85 (s, 1H), 6.27 (s, 2H), 6.12 (s, 2H), 3.85 (s, 3H), 3.78 (s, 3H), 3.68 (s, 6H), 3.63 (s, 3H). ^{13}C NMR (100 MHz, acetone-d_6) δ 162.3, 161.6, 160.8 (2C), 160.5, 157.5, 104.5, 104.3, 94.4, 92.1 (2C), 91.5, 56.1 (2C), 56.0, 55.7, 55.6, 55.5. HRMS (ESI) m/z calcd for $C_{17}H_{20}O_6Na$, $[M + Na]^+$: 343.1158, found: 343.1154.

8: beige solid; yield 24%. R_f = 0.21 (CyHex/EtOAc 6:4). ^{1}H NMR (400 MHz, acetone-d_6) δ 8.14 (s, 1H), 6.24 (s, 2H), 6.17 (s, 2H), 3.83 (s, 3H), 3.64 (s, 6H), 3.60 (s, 6H). ^{13}C NMR (100 MHz, acetone-d_6) δ 160.6, 159.4 (2C), 159.3 (2C),158.1, 105.6,104.1, 92.2 (2C), 91.0 (2C), 55.1 (2C), 54.9 (2C), 54.6.

Experimental procedure for the synthesis of compound 9

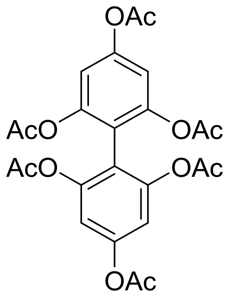

9

To a solution of phloroglucinol (1 g, 8 mmol, 1 eq) in MeOH (10 mL) a solution of $FeCl_3{\cdot}6H_2O$ (4.3 g, 16 mmol, 2 eq) in water (20 mL) was added dropwise. The mixture was stirred at room temperature for 96 h. MeOH was evaporated and the residue was diluted with water (30 mL) and extracted with EtOAc (50 mL × 4). The combined layers were dried over anhydrous Na_2SO_4 and concentrated in vacuum. The crude was dissolved in a solution of Ac_2O (15 mL) and pyridine (15 mL) at room temperature, stirring for 24 h. The mixture was diluted with EtOAc (100 mL), washed with 1 M HCl (100 mL × 2) and then quenched with a 5% $NaHCO_3$ solution (100 mL). The combined organic layers were washed with brine, dried over anhydrous Na_2SO4 and concentrated under reduced pressure. The residue was purified by flash chromatography (CyHex/EtOAc gradient from 8:2 to 0:100) to afford the desired product as a beige solid.

Yield 3%. R_f = 0.37 (CyHex/EtOAc 1:1). ^{1}H NMR (400 MHz, $CDCl_3$) δ 7.01 (s, 4H), 2.31 (s, 6H), 2.04 (s, 12H). ^{13}C NMR (100 MHz, $CDCl_3$) δ 168.3 (6C), 150.6 (2C), 149.3 (4C), 115.7 (2C),113.8 (4C), 21.2 (2C), 20.6 (4C).

Experimental procedure for the synthesis of 20

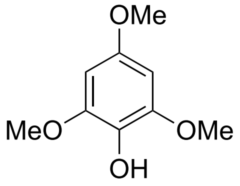

20

A solution of 3-chloroperbenzoic acid (17.6 g, 0.102 mol, 2 eq.) in dry DCM (165 mL) was dropped into a solution of commercially available 2,4,6-trimethoxy benzaldehyde **19** (10.0 g, 0.051 mol, 1 eq.) in dry DCM (60 mL) under nitrogen atmosphere, at 0 °C in 45 min. The reaction mixture was heated to room temperature and stirred overnight, then was washed with a saturated solution of $NaHCO_3$. The organic phase was washed with brine, dried over Na_2SO_4, filtered, and the solvent was evaporated under reduced pressure. The resulting crude was dissolved in MeOH (85 mL) and cooled at 0 °C. 3.5 M KOH (51 mL) aqueous solution was added dropwise and the reaction was stirred at 0 °C for 45 min. Concentrated HCl was added until pH = 2, and the aqueous phase was extracted with EtOAc. The collected organic phases were washed with brine, dried over

Na_2SO_4, filtered, and the solvent was evaporated under reduced pressure. The crude product was purified by flash column chromatography (Hex/EtOAc 7:3) to obtain a yellow solid.

Yield: 47%. $R_f = 0.50$ (Hex/EtOAc 1:1). ^1H NMR (400 MHz, $CDCl_3$) δ 6.19 (s, 2H), 5.10 (s, 1H), 3.87 (s, 6H), 3.77 (s, 3H). [1]

General Procedure for Ullman reaction

The solid reagents were placed in a pyrex screw cap tube, equipped with a stirring bar, in the following order: CuI (0.11 eq), picolinic acid (0.21 eq), phenol (1.07 eq), aryl bromide (1.00 eq), and K_2PO_4 (1.96 eq.). Finally dry DMSO (0.3 M) was added and the mixture was stirred in closed atmosphere at 130 °C. After 5 days the crude was cooled at room temperature and diluted with water and EtOAc. The aqueous layer was extracted with EtOAc, the collected organic phases were washed with brine, dried over Na_2SO_4, filtered, and the solvent was evaporated under reduced pressure. The crude products were purified by flash column chromatography as described below.

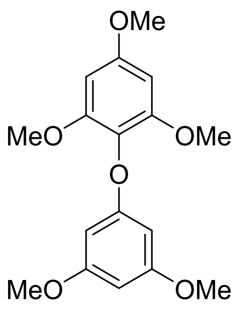

10

10: the general procedure for Ullman reaction was applied to **20** and 1-bromo-3,5-dimethoxybenzene **21**. The crude product was purified by flash column chromatography (Hex/EtOAc gradient from 9:1 to 8:2) affording the desired product as a white solid.

Yield: 56%. $R_f = 0.25$ (Hex/EtOAc 8:2). ^1H NMR (400 MHz, $CDCl_3$) δ 6.20 (s, 2H), 6.10 (t, $J = 2.2$ Hz, 1H), 6.07 (d, $J = 2.2$ Hz, 2H), 3.82 (s, 3H), 3.77 (s, 6H), 3.77 (s, 6H). ^{13}C NMR (100 MHz, $CDCl_3$) δ 161.5 (2C), 160.9, 157.8, 153.9 (2C), 126.0, 93.9, 93.8 (2C), 91.9 (2C), 56.4 (2C), 55.7, 55.4 (2C). HRMS (ESI) m/z calcd for $C_{17}H_{20}O_6Na$, $[M + Na]^+$: 343.1158, found: 343.1154.

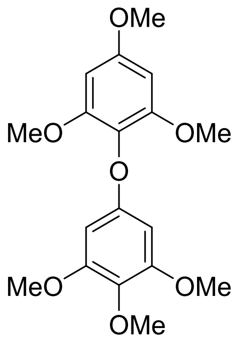

11

11: the general procedure for Ullman reaction was applied to **20** and 5-bromo-1,3,5-trimethoxybenzene **21**. The crude product was purified by flash column chromatography (Hex/EtOAc gradient from 8:2 to 7:3) affording the desired product as a white solid.

Yield: 24%. $R_f = 0.20$ (Hex/EtOAc 7:3). ^{1}H NMR (400 MHz, $CDCl_3$) δ 6.23 (s, 2H), 6.12 (s, 2H), 3.84 (s, 3H), 3.79 (s, 6H), 3.78 (s, 3H), 3.75 (s, 6H). ^{13}C NMR (100 MHz, $CDCl_3$) δ 157.8, 155.4, 154.0 (2C), 153.7 (2C), 132.8, 126.1, 92.3 (2C), 92.0 (2C), 61.1, 56.4 (2C), 56.1 (2C), 55.7.

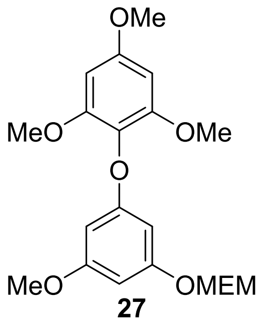

27

27: the general procedure for Ullman reaction was applied to **20** and aryl bromide **25**. The crude product was purified by flash column chromatography (Hex/EtOAc gradient from 7:3 to 6:4) affording the desired product as a viscous oil.

Yield: 31%. $R_f = 0.15$ (Hex/EtOAc 7:3). ^{1}H NMR (400 MHz, $CDCl_3$) δ 6.27 (t, $J = 2.2$ Hz, 1H), 6.20 (s, 2H), 6.16 (t, $J = 2.2$ Hz, 1H), 6.13 (t, $J = 2.2$ Hz, 1H), 5.19 (s, 2H), 3.82 (s, 3H), 3.81 – 3.77 (m, 2H), 3.77 (s, 6H), 3.73 (s, 3H), 3.56 – 3.52 (m, 2H), 3.37 (s, 3H). ^{13}C NMR (100 MHz, $CDCl_3$) δ 161.4, 160.8, 159.1, 157.8, 153.9 (2C), 126.1, 96.1, 95.8, 95.1, 93.7, 92.0 (2C), 71.7, 67.9, 59.2, 56.4 (2C), 55.7, 55.5.

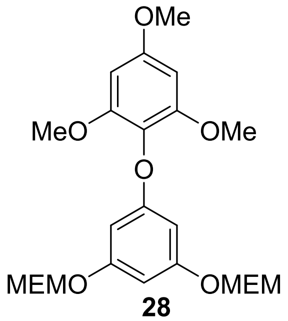

28

28: the general procedure for Ullman reaction was applied to **20** and aryl bromide **26**. The crude product was purified by flash column chromatography (Hex/EtOAc gradient from 6:4 to 1:1) affording the desired product as a viscous oil.

Yield: 31% yield. R_f = 0.13 (Hex/EtOAc 7:3). ^{1}H NMR (400 MHz, $CDCl_3$) δ 6.40 (t, J = 2.2 Hz, 1H), 6.23 (d, J = 2.1 HZ, 2H), 6.20 (s, 2 H), 5.18 (s, 4H), 3.82 (s, 3H), 3.80 – 3.77 (m, 4H), 3.76 (s, 6H), 3.57- 3.51 (m, 4H), 3.37 (s, 6H). ^{13}C NMR (100 MHz, $CDCl_3$) 160.7, 159.0 (2C), 157.8, 153.9 (2C), 126.1, 98.1, 97.3 (2C), 93.7 (2C), 92.0 (2C), 71.7 (2C), 67.9 (2C), 59.2 (2C), 56.4 (2C), 55.7.

Experimental procedure for the synthesis of 23

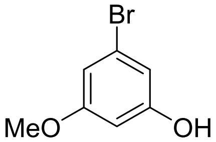

23

NaOH (1.1 g, 27.6 mmol, 3 eq.) and 1-dodecanthiol (3.3 mL, 13.8 mmol, 1.5 eq.) were added to a solution of 1-bromo-3,5-dimethoxybenzene **21** (2 g, 9.2 mmol, 1eq.) in dry NMP (9.2 mL) in a pyrex screw cap tube. The reaction was heated at 130 °C and stirred for 2 h, then was cooled and acidified with 1 M HCl. The aqueous layer was extracted with EtOAc, the collected organic phases were washed with brine, dried over Na_2SO_4, filtered, and the solvent was evaporated under reduced pressure. The crude products were purified by flash column chromatography (Hex/EtOAc 8:2) affording the desired product as white solid.

Yield: 78%. R_f = 0.24 (Hex/EtOAc 8:2). ^{1}H NMR (400 MHz, $CDCl_3$) δ 6.65 (t, J = 2.2 Hz, 1H), 6.61 (t, J = 2.2 Hz, 1H), 6.33 (t, J = 2.2 Hz, 1H), 4.87 (bs, 1H), 3.76 (s, 3H). [2]

General Procedure for MEM-protection of phenols

DIPEA (2 eq. for each -OH group) was added to a solution of phenol (1 eq.) in dry THF (0.3 M) under nitrogen atmosphere and was stirred at 0 °C for 10 min. MEM-Cl (2 eq. for each -OH group) was added to the reaction mixture which was stirred at room temperature overnight and then acidified with 1 M HCl. The aqueous layer was extracted with EtOAc, the collected organic phases were washed with brine, dried over Na_2SO_4, filtered, and the solvent as evaporated under reduced pressure. The crude products were purified by flash column chromatography as described below.

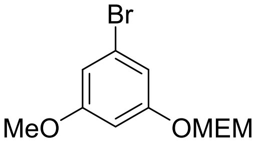

25

25: was obtained from MEM-protection of **23**. The crude product was purified by flash chromatography (Hex/MeOAc 9:1) affording the desired product as a transparent oil.

Yield: 66%. R_f = 0.40 (Hex/MeOAc 8:2). ^{1}H NMR (400 MHz, $CDCl_3$) δ 6.83 (t, *J* = 2.2 Hz, 1H), 6.71 (t, *J* = 2.2 Hz, 1H), 6.54 (t, *J* = 2.2 Hz, 1H), 5.22 (s, 2H), 3.84 – 3.79 (m, 2H), 3.77 (s, 3H), 3.58 – 3.52 (m, 2H), 3.38 (s, 3H). ^{13}C NMR (100 MHz, $CDCl_3$) δ 161.3, 159.0, 123.0, 112.3, 111.2, 101.9, 93.7, 71.7, 68.0, 59.2, 55.7.

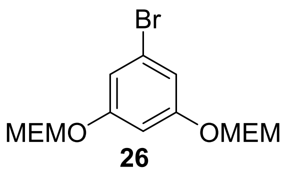

26

26: was obtained from MEM-protection of **24**. The crude product was purified by flash chromatography (Hex/MeOAc 7:3) affording the desired product as a light-yellow oil.

Yield: 55%. R_f = 0.34 (Hex/MeOAc 7:3). ^{1}H NMR (400 MHz, $CDCl_3$) δ 6.89 (d, *J* = 2.2 Hz, 2H), 6.67 (t, *J* = 2.2 Hz, 1H), 5.22 (s, 4H), 3.83 – 3.77 (m, 4H), 3.59 – 3.52 (m, 4H), 3.38 (s, 6H). ^{13}C NMR (100 MHz, $CDCl_3$) δ 158.8 (2C), 122.9, 113.4 (2C), 104.2, 93.7 (2C), 71.7 (2C), 68.0 (2C), 59.2 (2C).

General Procedure for MEM-deprotection.

Concentrated HCl (12 M, 3 eq. for each -OMEM group) was added to a solution of MEM-protected intermediate (1 eq.) in MeOH (0.1 M) and was stirred at room temperature overnight. The solvent was concentrated in vacuum, then water was added, and the aqueous phase was extracted with EtOAc. The collected organic phases were washed with brine, dried over Na_2SO_4, filtered, and the solvent as evaporated under reduced pressure. The crude products were purified by flash column chromatography as described below.

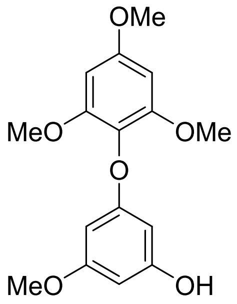

14

14: was obtained from MEM-deprotection of **27**. The crude product was purified by flash column chromatography (Hex/EtOAc 6:4) affording the desired product as a yellow sticky solid.

Yield: 56%. R_f = 0.18 (Hex/EtOAc 7:3). ^{1}H NMR (400 MHz, acetone-d_6) δ 8.20 (s, 1H), 6.34 (s, 2H), 6.02 (t, *J* = 2.2 Hz, 1H), 5.90 (t, *J* = 2.2 Hz, 1H), 5.83 (t, *J* = 2.1 Hz, 1H), 3.83 (s, 3H), 3.74 (s, 6H), 3.68 (s, 3H). ^{13}C NMR (100 MHz, acetone–d_6) δ 162.6, 162.2, 160.0, 158.9, 154.9 (2C), 126.9, 95.6, 95.4, 93.8, 92.8 (2C), 56.5 (2C), 56.0, 55.5. HRMS (ESI) m/z calcd for $C_{16}H_{18}O_6Na$, $[M + Na]^+$: 329.1001, found: 329.1010.

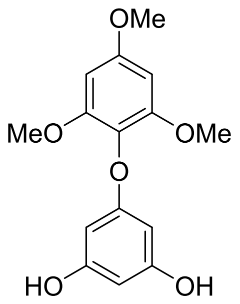

15

15: was obtained from MEM-deprotection of **28**. The crude product was purified by flash column chromatography (Hex/EtOAc gradient from 1:1 to 4:6) affording the desired product as a white solid.

Yield: 63%. R_f = 0.30 (Hex/EtOAc 1:1). ^{1}H NMR (400 MHz, acetone-d_6) δ 8.07 (s, 2H), 6.33 (s, 2H), 5.96 (t, J = 2.1 Hz, 1H), 5.80 (d, J = 2.1 Hz, 2H), 3.83 (s, 3H), 3.74 (s, 6H). ^{13}C NMR (100 MHz, acetone-d_6) δ 162.2, 159.8 (2C), 158.7, 154.9 (2C), 126.9, 96.8, 94.8 (2C), 92.7 (2C), 56.4 (2C), 55.8. HRMS (ESI) m/z calcd for $C_{15}H_{16}O_6Na$, $[M + Na]^+$: 315.0845, found: 315.0846.

General Procedure for methoxy-deprotection

1 M BBr_3 solution in DCM (2 eq. for each -OMe group) was added to a solution of permethylated derivative (1 eq) in dry DCM (0.12 M) under nitrogen atmosphere at –78 °C. The reaction was stirred at the same temperature for 1 h and then at room temperature overnight. The reaction was cooled at 0 °C and then quenched with water, then the organic solvent was evaporated under reduced pressure. The remaining aqueous phase was extracted with EtOAc, the collected organic phases were washed with brine, dried over Na_2SO_4, filtered, and the solvent was evaporated under reduced pressure. The crude products were purified by flash column chromatography as described below.

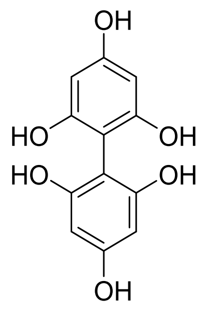

5

5: the crude was purified by column chromatography (DCM/MeOH 92:8). to afford the product as a white solid.

Yield: 77%. R_f = 0.25 (DCM/MeOH 92:8). ^{1}H NMR (400 MHz, acetone-d_6) δ 8.15 (s, 2H), 7.25 (s, 4H), 6.03 (s, 4H). ^{13}C NMR (100 MHz, acetone-d_6) δ 158.8 (2C), 157.5 (4C), 98.2 (2C), 95.1 (4C). HRMS (ESI) m/z calcd for $C_{12}H_9O_6$, $[M – H]^-$: 249.0399, found: 249.0402.

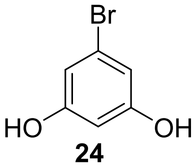

24

24: was added obtained from fully deprotection of 1-bromo-3,5-dimethoxybenzene **21**. The crude products were purified by flash column chromatography (Hex/EtOAc gradient from 8:2 to 7:3 + 1% of formic acid) affording the desired product as white solid.

Yield: 89%. R_f = 0.32 (Hex/EtOAc 7:3 + 1% formic acid). ^{1}H NMR (400 MHz, DMSO-d_6) δ 9.68 (bs, 2H), 6.37 (d, J = 2.1 Hz, 2H), 6.18 (t, J = 2.1 Hz, 1H). [3]

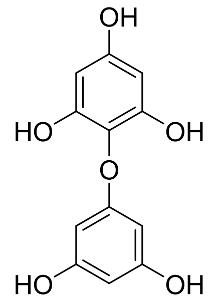

12
DIPHLORETHOL

12 (diphloroethol): was obtained from complete deprotection of **10**. The crude product was purified by flash column chromatography (DCM/MeOH 9:1 + 1% formic acid) affording the desired product as a brownish solid.

Yield: 63%. R_f = 0.19 (DCM/MeOH 9:1 + 1% formic acid). ^{1}H NMR (400 MHz, CD_3OD) δ 5.94 (s, 2H), 3.90 – 3.88 (m, 3H). ^{13}C NMR (100 MHz, CD_3OD) δ 162.1, 159.9 (2C), 156.2, 152.3 (2C), 124.9, 97.2, 96.3 (2C), 95.4 (2C). HRMS (ESI) m/z calcd for $C_{12}H_9O_6$, $[M - H]^-$: 249.0399, found: 249.0403.

Experimental procedure for the synthesis of 13

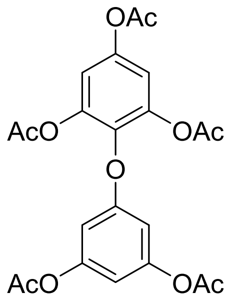

13

DMAP (6.1 mg, 0.05 mmol, 0.25 eq.) and triethylamine (167 µL, 1.2 mmol, 6 eq.) were added to a solution of **12** in THF (0.5 mL). Acetic anhydride (113 µL, 1.2 mmol, 6 eq.) was dropped into the reaction mixture, which was stirred at room temperature for 7 h, then diluted with DCM and washed with water. The organic phase was washed with brine, dried over Na_2SO_4, filtered, and the solvent as evaporated under reduced pressure. The crude products were purified by flash column chromatography (Hex/EtOAc 6:4) affording the desired product as white solid.

Yield: 63%. R_f = 0.44 (Hex/EtOAc 1:1). ^{1}H NMR (400 MHz, $CDCl_3$) δ 6.95 (s, 2H), 6.64 (t, J = 2.0 Hz, 1H), 6.57 (d, J = 2.0 Hz, 2H), 2.28 (s, 3H), 2.25 (s, 6H), 2.07 (s, 6H). ^{13}C NMR (100 MHz, $CDCl_3$) δ 168.7 (2C), 168.5, 167.9 (2C), 158.1, 151.1 (2C), 146.7, 143.7 (2C), 136.4, 115.0 (2C), 110.5, 107.5 (2C), 21.2, 21.1 (2C), 20.4 (2C).

General procedure for 26, 27 and 28

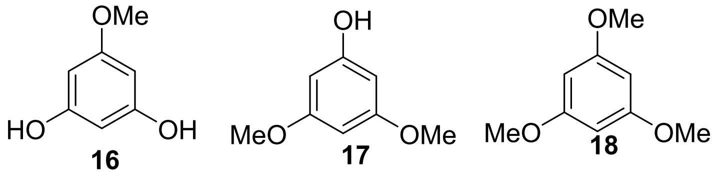

To a solution of phloroglucinol (2 g, 15.8 mmol, 1 eq) in acetone (30 mL) was added K_2CO_3 (2.19 g), 15.8 mmol, 1 eq). Then, dimethyl sulphate (0.33 eq for **16** and **17**, 3 eq for **18**) was added dropwise. The mixture was stirred at 55 °C (24 h for **16** and **17**, 3 h for **18**). The reaction was quenched with 1 M HCl (80 mL). The aqueous phase was extracted with EtOAc (4 × 100 mL). The combined organic layers were washed with a solution of brine, dried over Na_2SO_4 and concentrated under reduced pressure. The residue was purified by flash chromatography (CyHex/EtOAc gradient 8:2 to 0:100).

16: white solid; yield: 13%. R_f = 0.71 (CyHex/EtOAc 85:15). ^{1}H NMR (400 MHz, acetone-*d6*) δ 6.10 (s, 3H), 3.76 (s, 9H).

17: beige solid; yield: 53%. R_f = 0.42 (CyHex/EtOAc 85:15). ^{1}H NMR (400 MHz, acetone-*d6*) δ 8.28 (s, 1H), 6.04 (d, *J* = 2.1 Hz, 2H), 6.01 (t, 1H), 3.73 (s, 6H).

18: beige solid; yield: 31%. R_f = 0.21 (CyHex/EtOAc 85:15). ^{1}H NMR (400 MHz, acetone-*d6*) δ 8.14 (s, 2H), 5.99 (t, *J* = 2.1 Hz, 1H), 5.94 (d, *J* = 2.1 Hz, 2H), 3.70 (s, 3H).

[1] Gurale BP, Shashidhar MS, Sardessai RS, Gonnade RG. Inositol to aromatics –benzene free synthesis of poly oxygenated aromatics. Carbohydr Res 2018;461:38–44. https://doi.org/10.1016/j.carres.2018.03.007.

[2] Lindgren AEG, Öberg CT, Hillgren JM, Elofsson M. Total synthesis of the resveratrol oligomers (±)-Ampelopsin B and (±)-σ-Viniferin. European J Org Chem 2016;2016:426–9. https://doi.org/10.1002/ejoc.201501486.

[3] Bloemendal VRLJ, Sondag D, Elferink H, Boltje TJ, van Hest JCM, Rutjes FPJT. A Revised Modular Approach to (–)-trans-Δ 8 -THC and Derivatives Through Late-Stage Suzuki–Miyaura Cross-Coupling Reactions. European J Org Chem 2019;2019:2289–96. https://doi.org/10.1002/ejoc.201900059.

NMR Spectra

Figure S1. ^{1}H (top) and ^{13}C (bottom) NMR of compound 1.

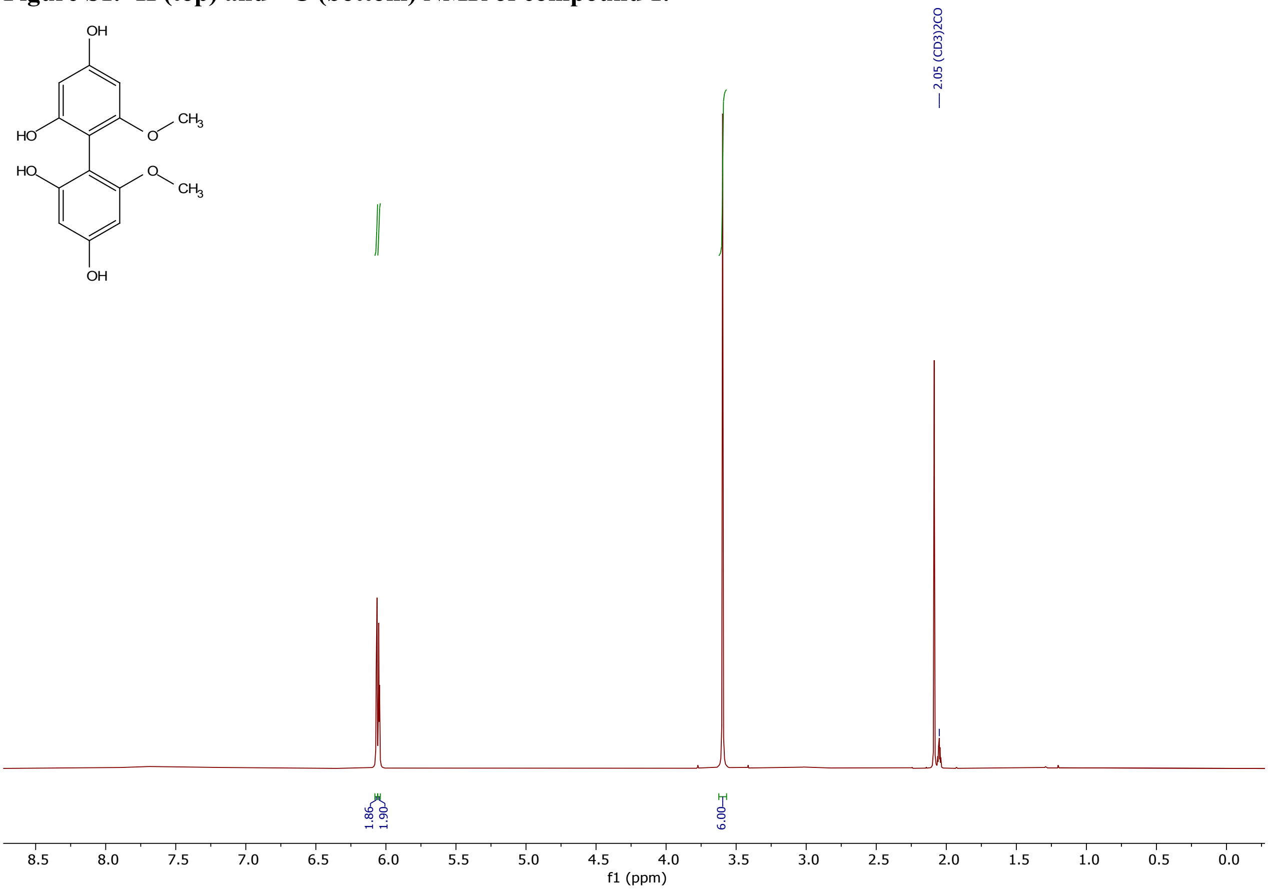

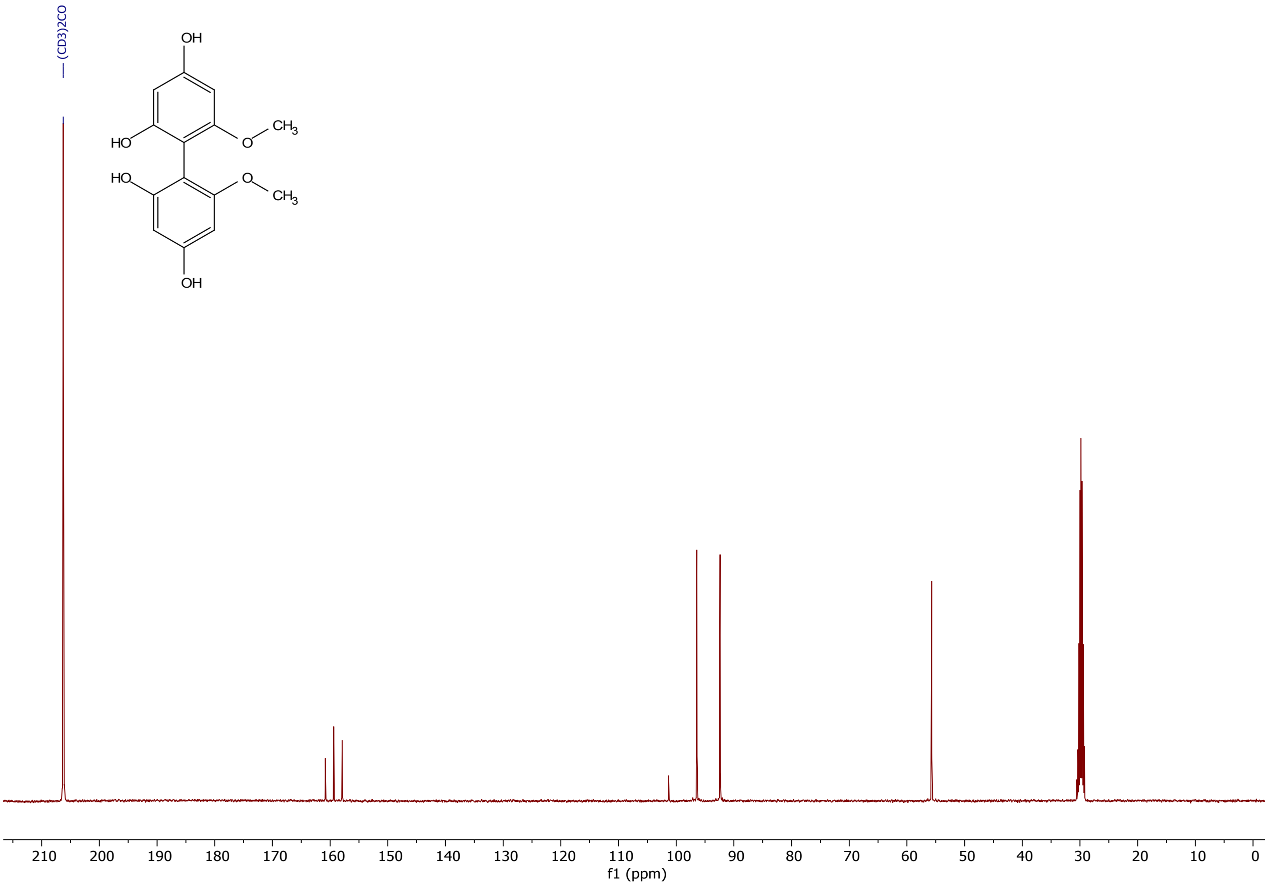

Figure S2. ^{1}H (top) and ^{13}C (bottom) NMR of compound 2.

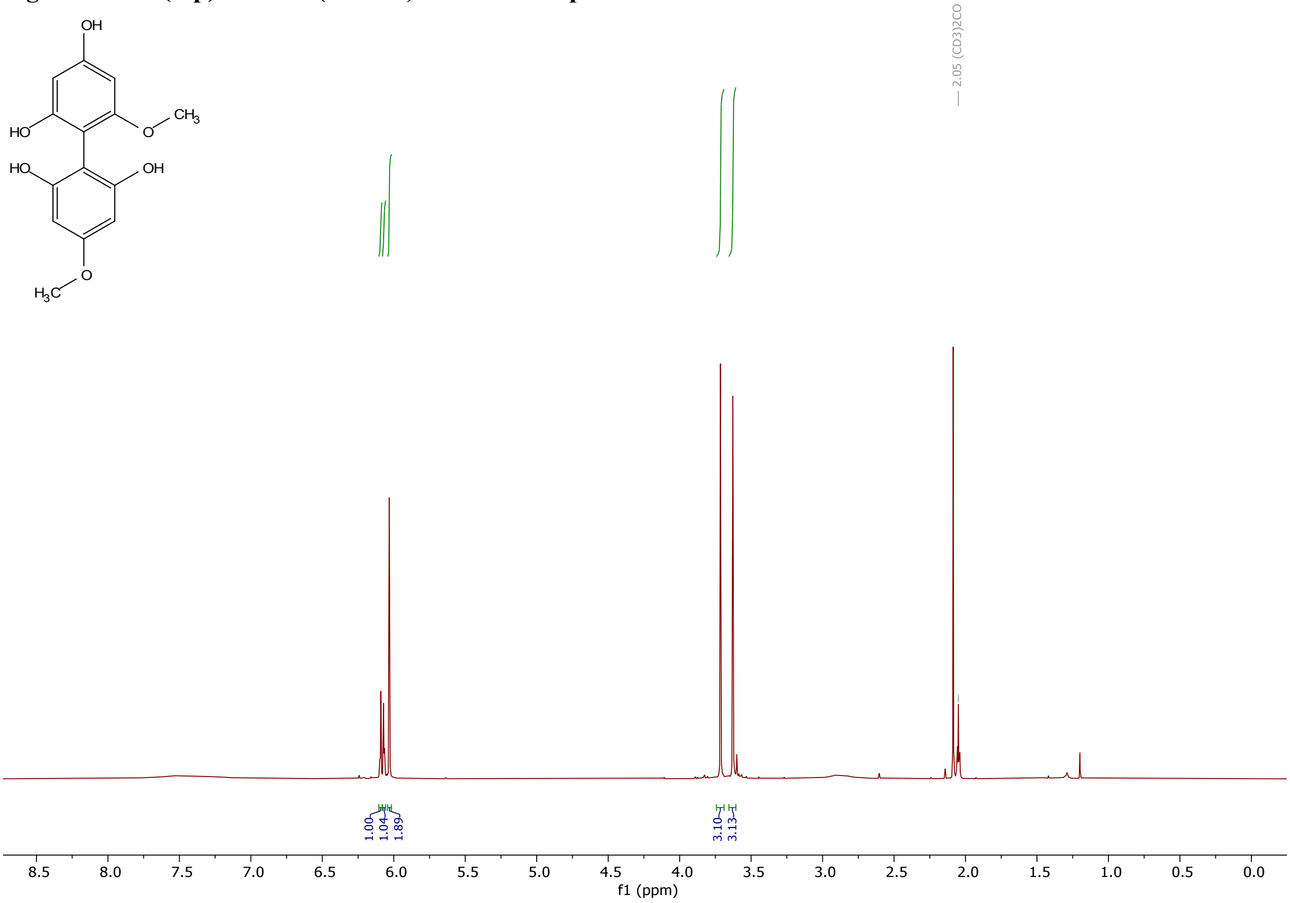

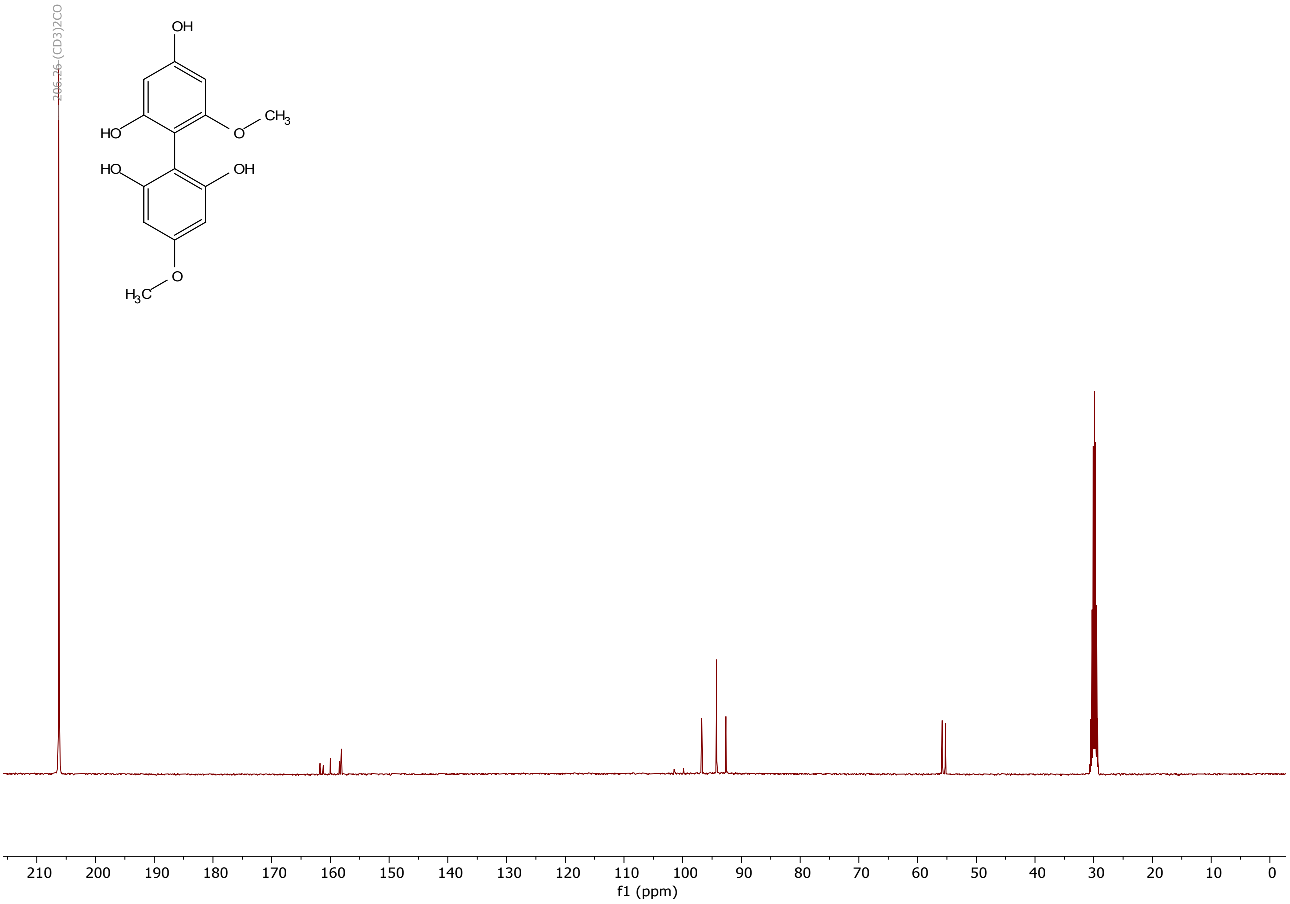

Figure S3. ^{1}H (top) and ^{13}C (bottom) NMR of compound 3.

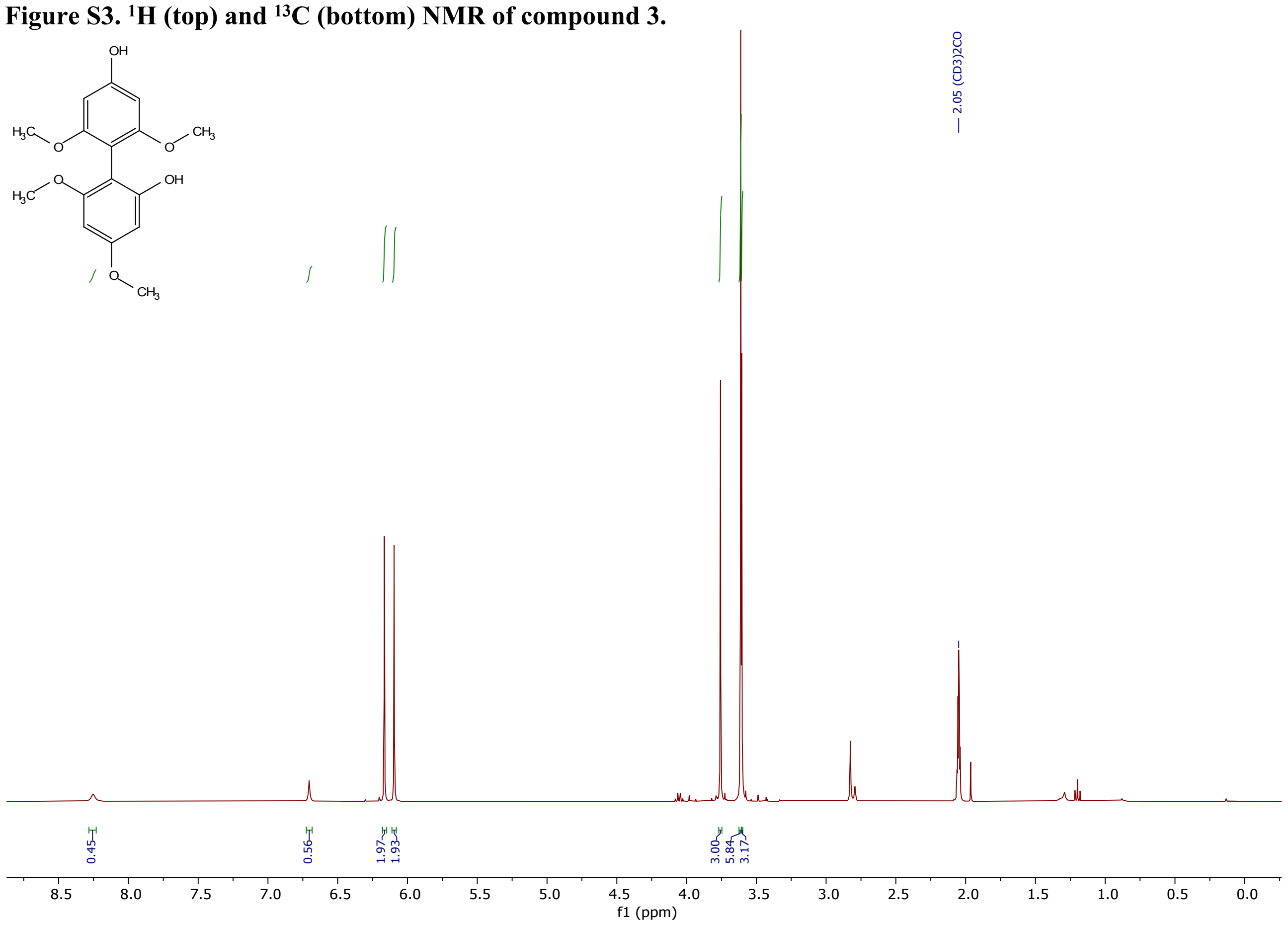

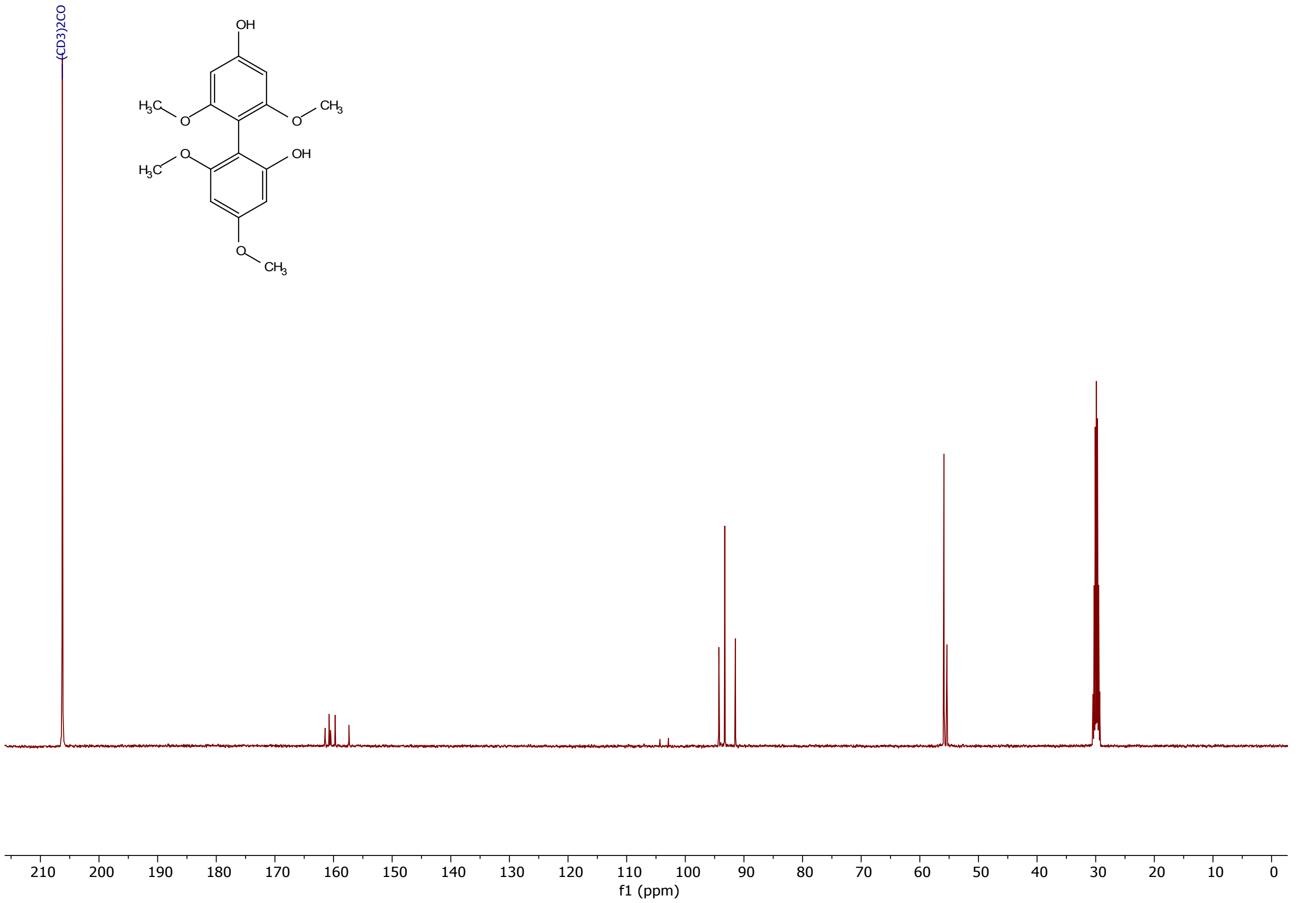

Figure S4. ^{1}H (top) and ^{13}C (bottom) NMR of compound 4.

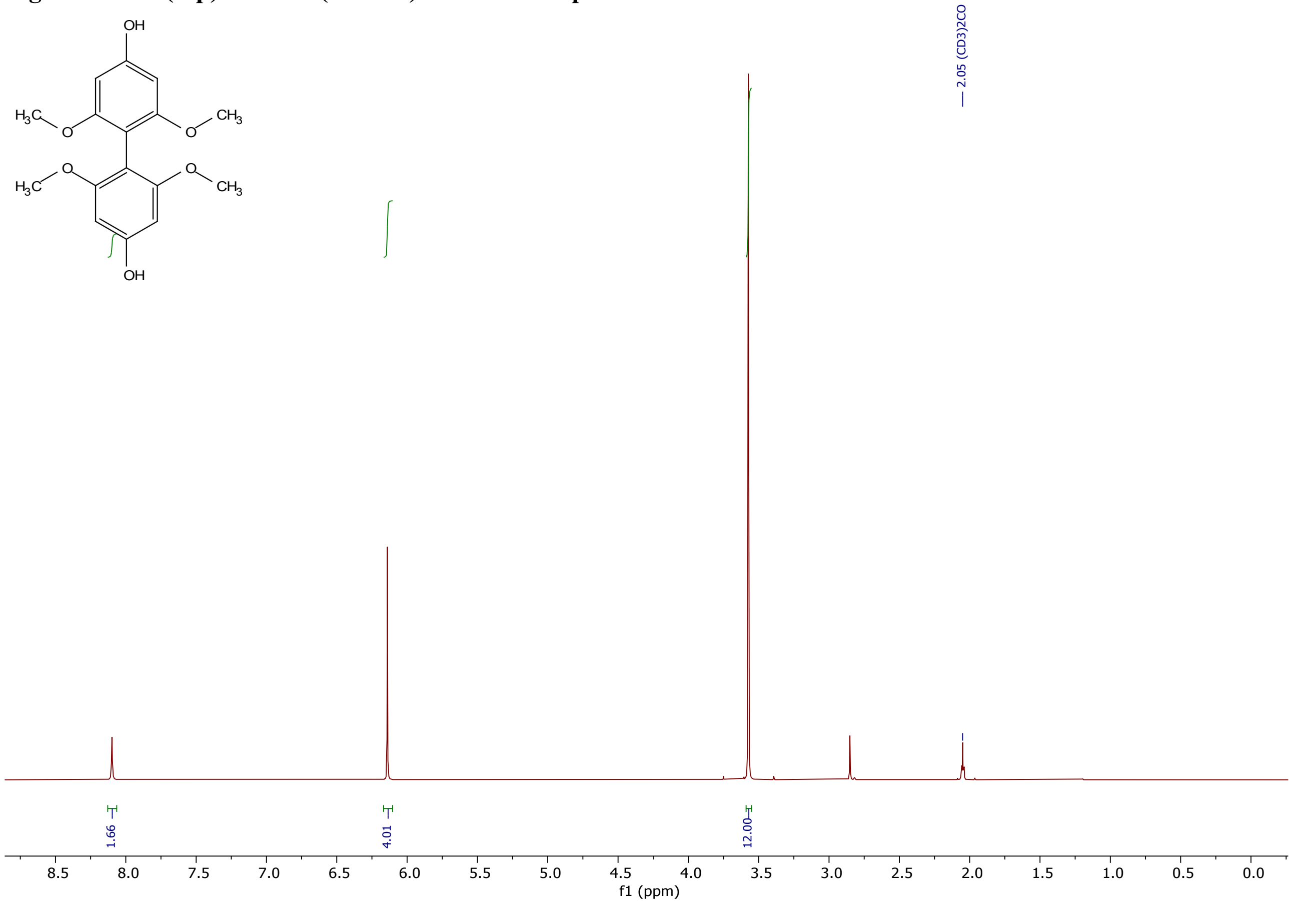

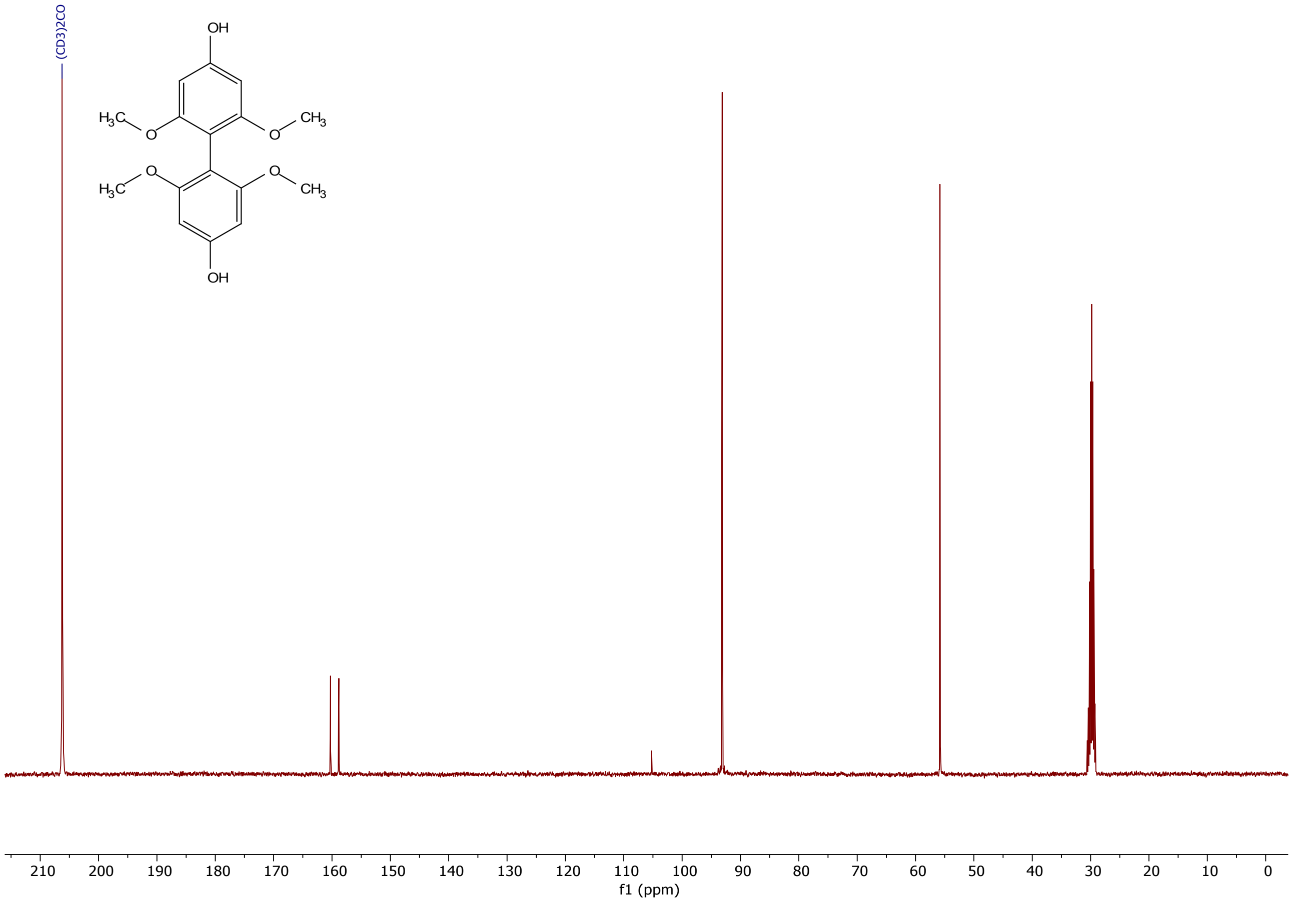

Figure S5. ^{1}H **(top) and** ^{13}C **(bottom) NMR of compound 5.**

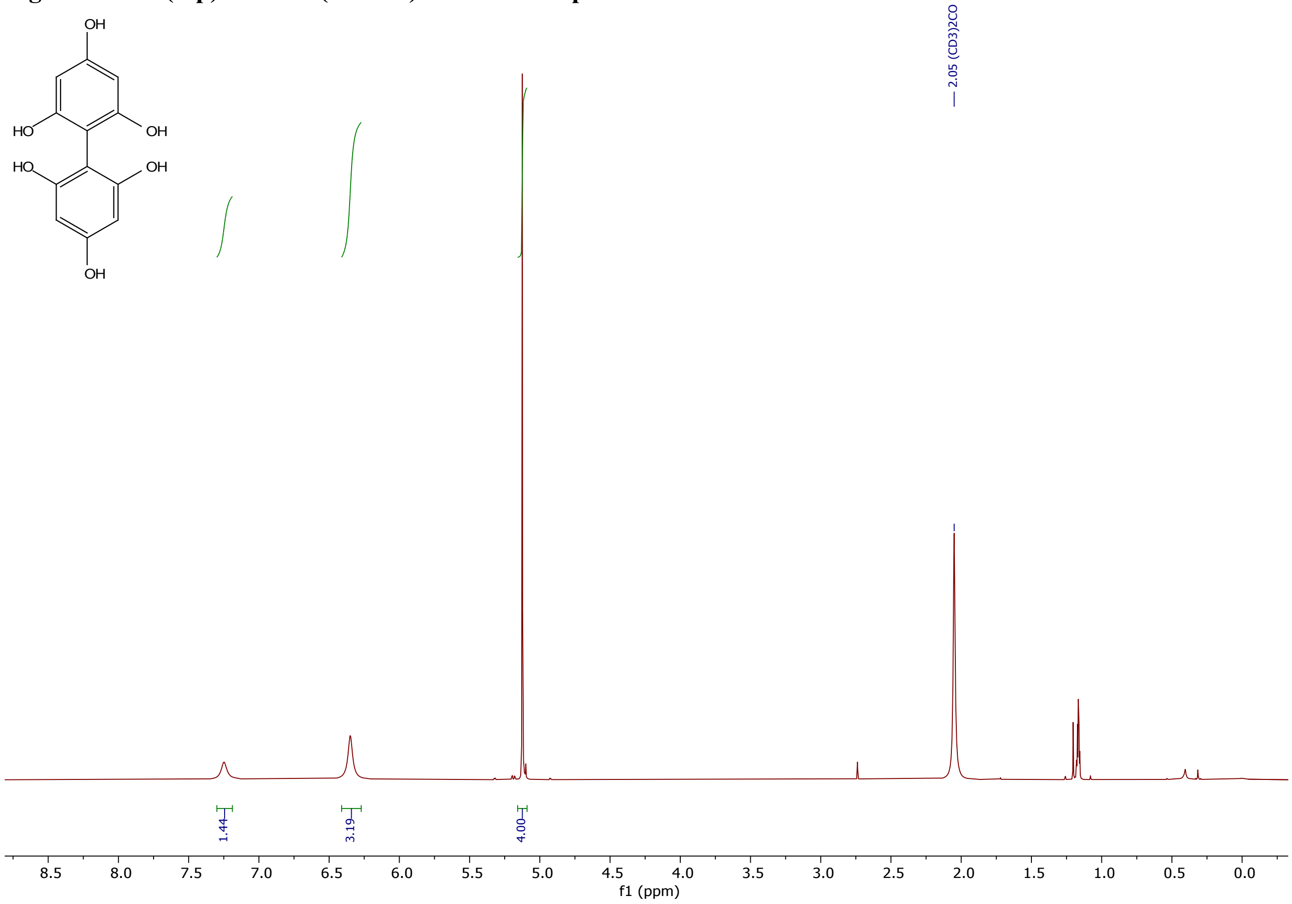

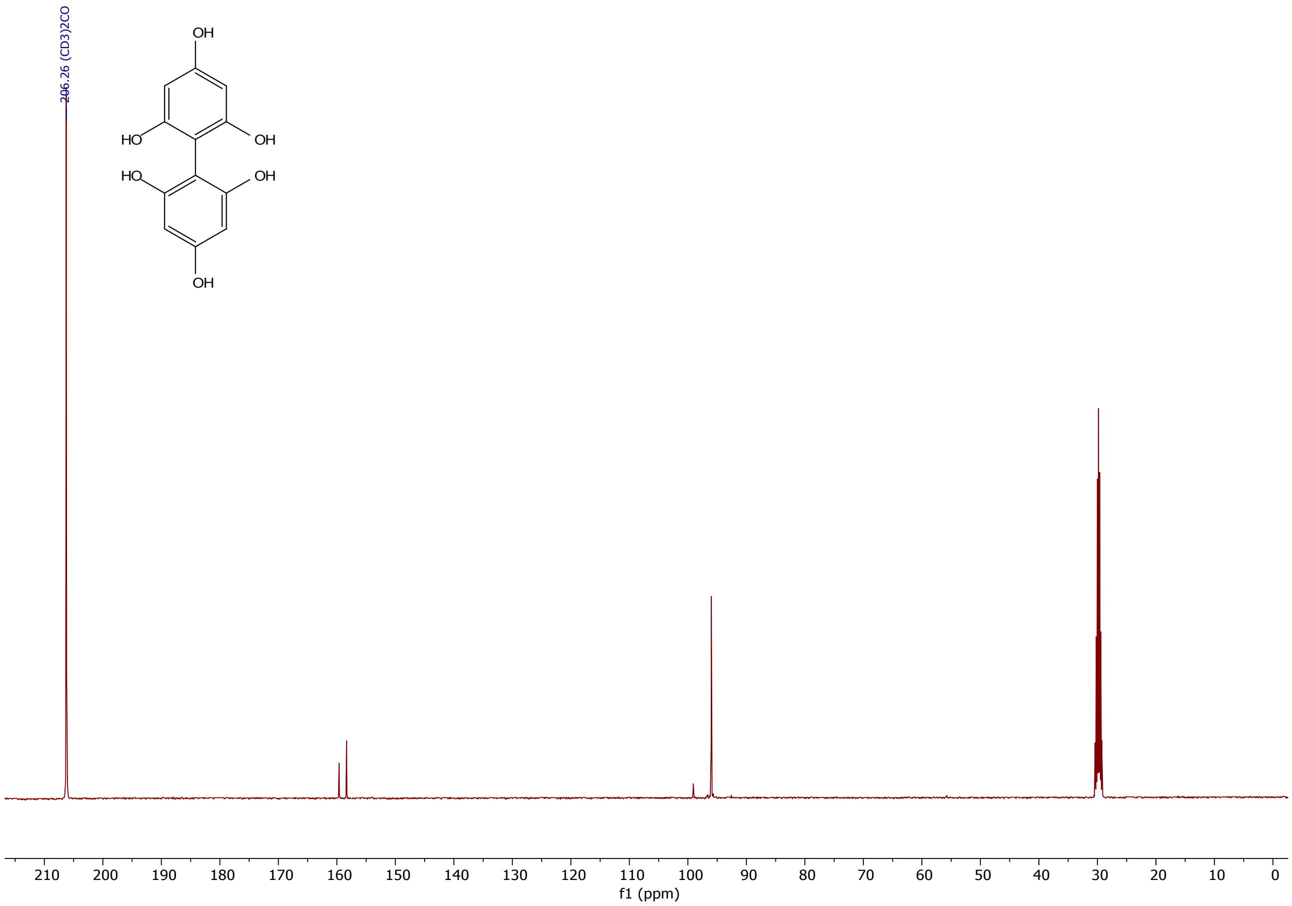

Figure S6. ^{1}H (top) and ^{13}C (bottom) NMR of compound 6.

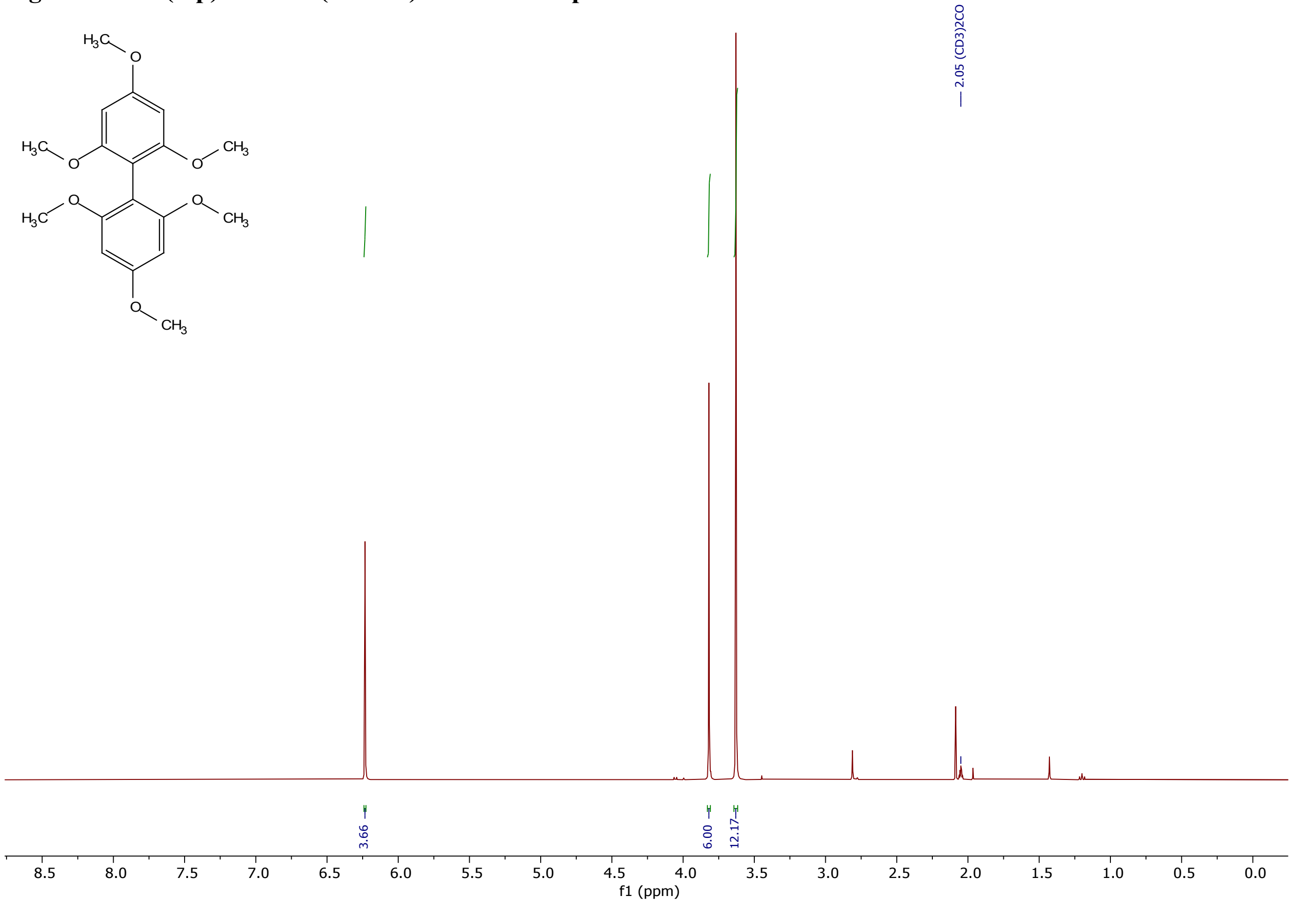

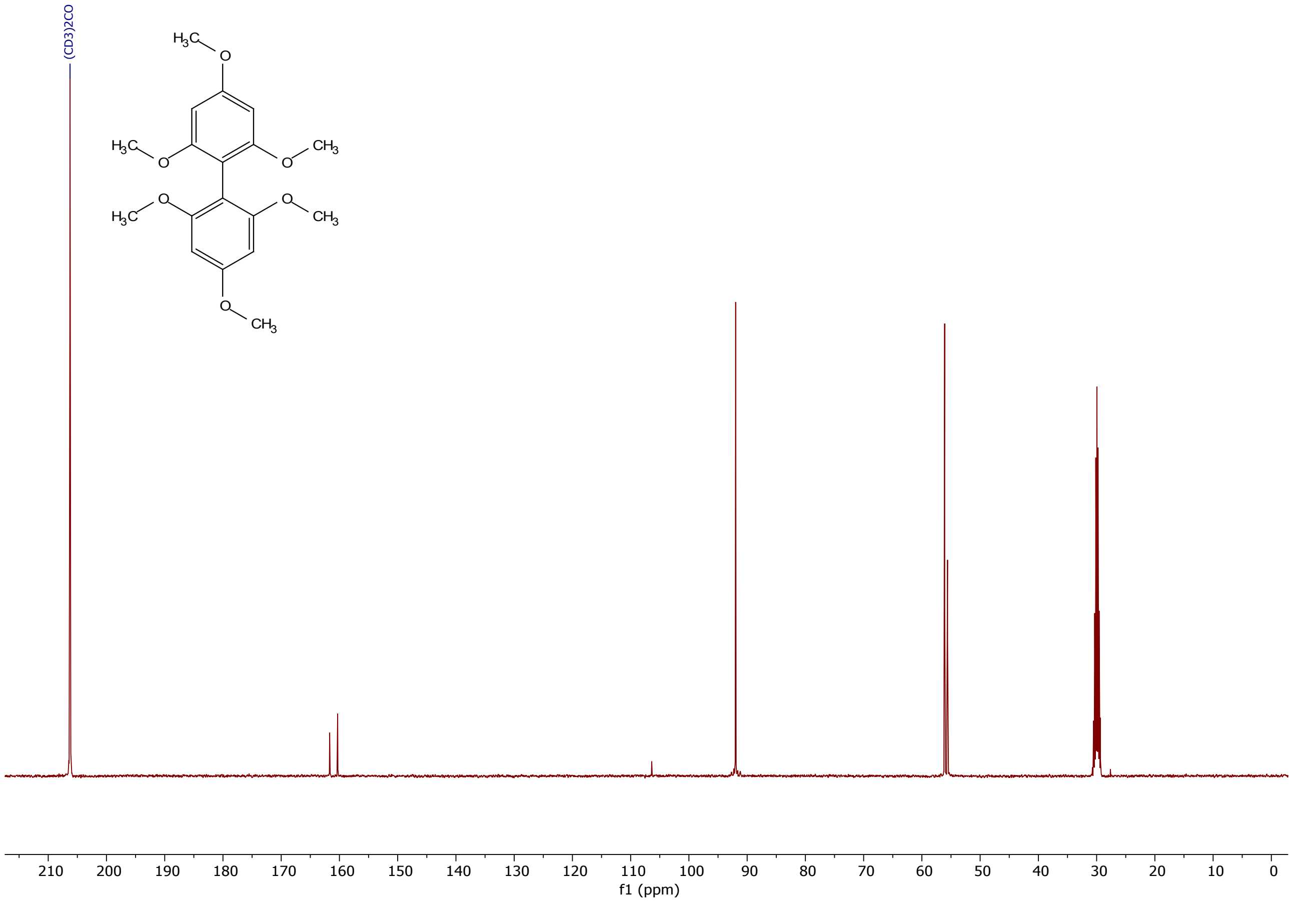

Figure S7. ^{1}H (top) and ^{13}C (bottom) NMR of compound 7.

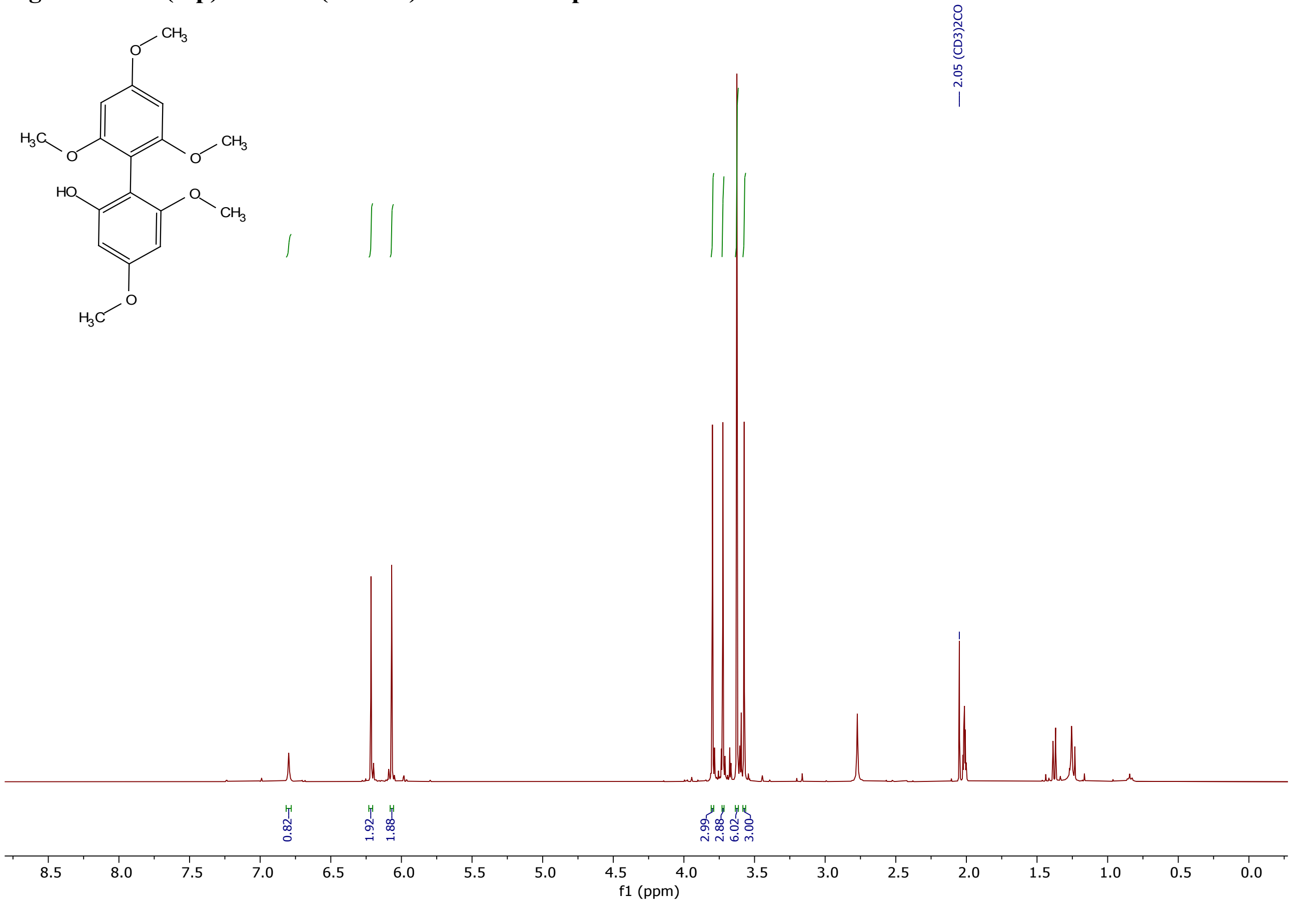

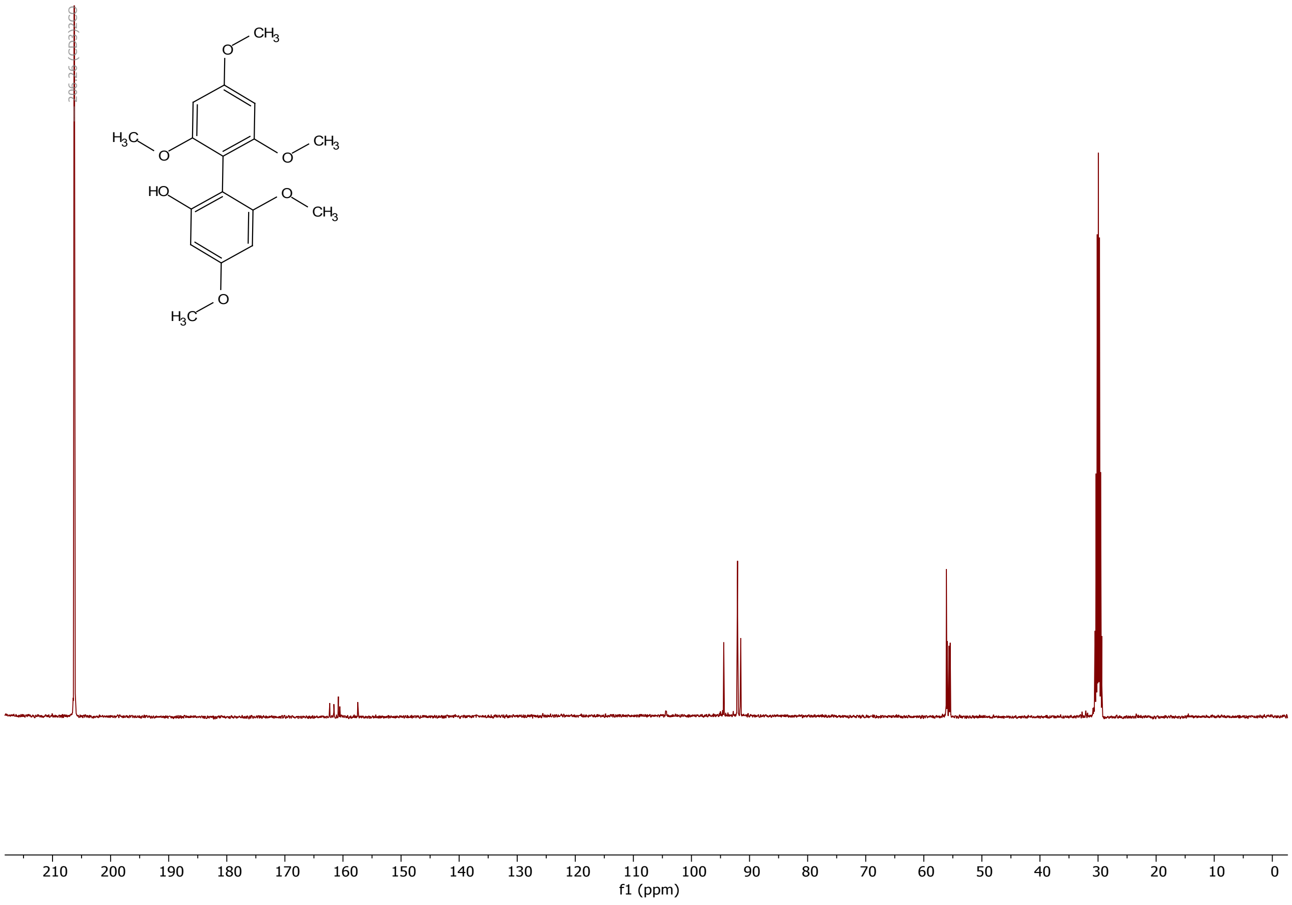

Figure S8. ^{1}H (top) and ^{13}C (bottom) NMR of compound 8.

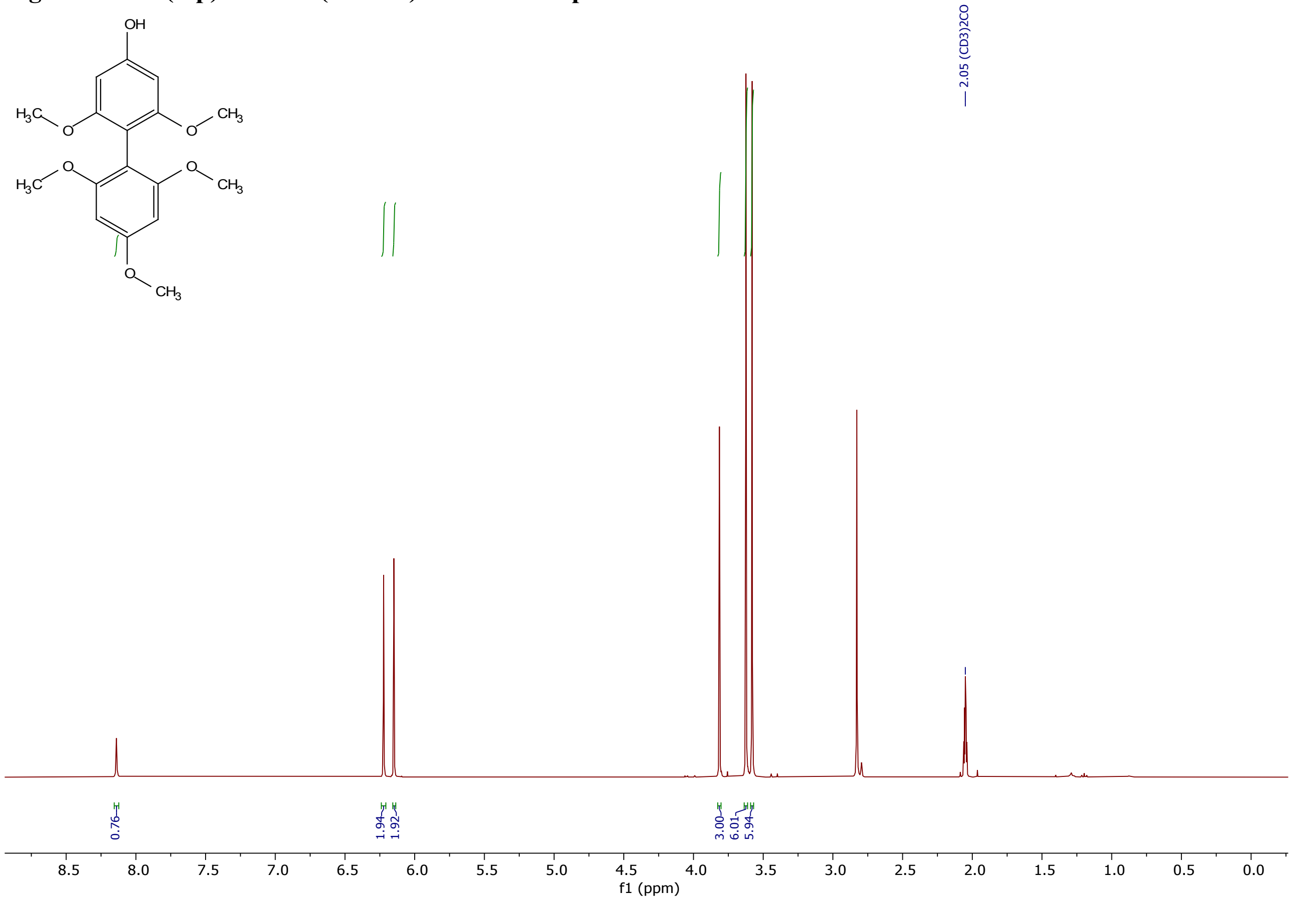

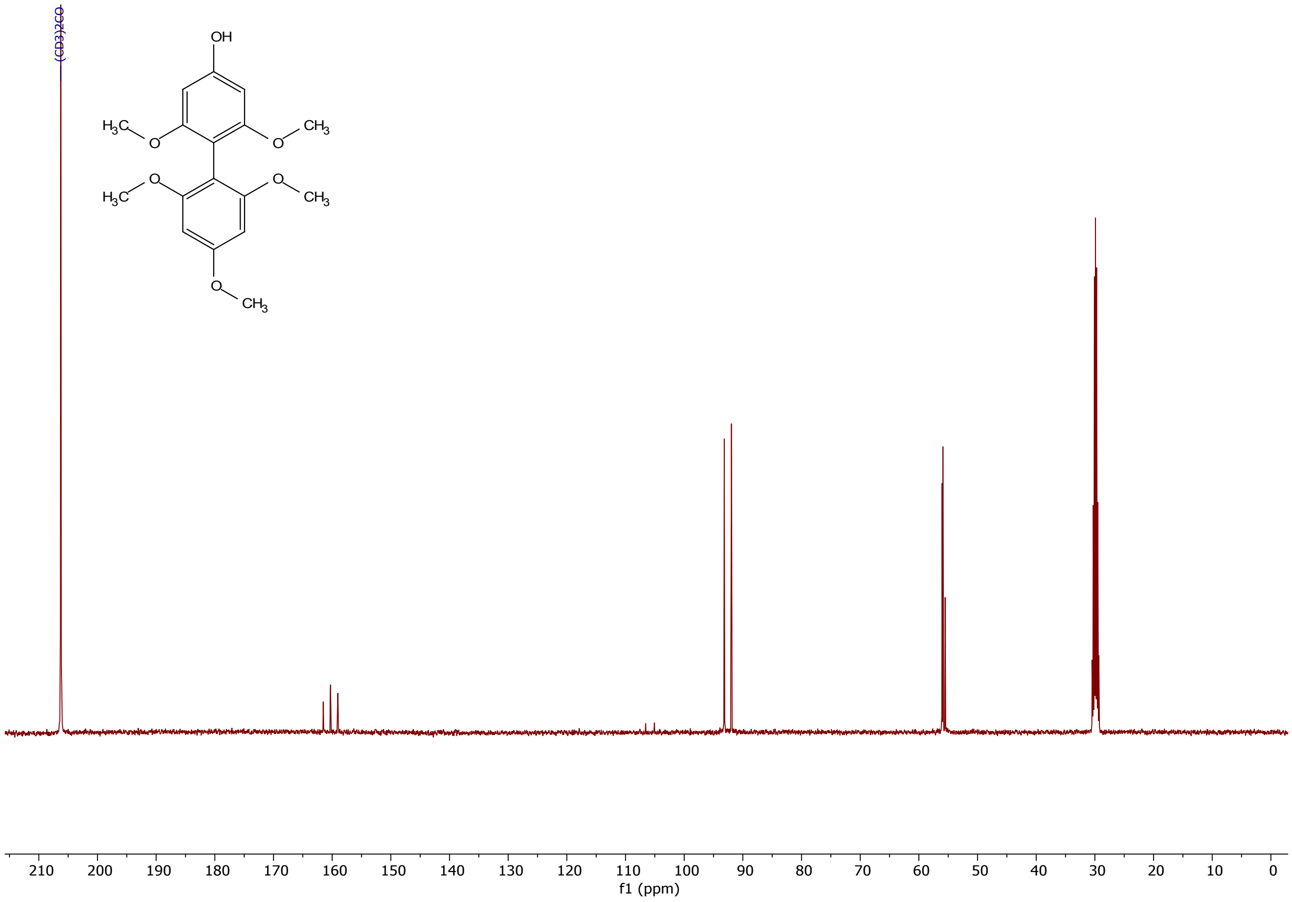

Figure S9. [1]**H (top) and** [13]**C (bottom) NMR of compound 9.**

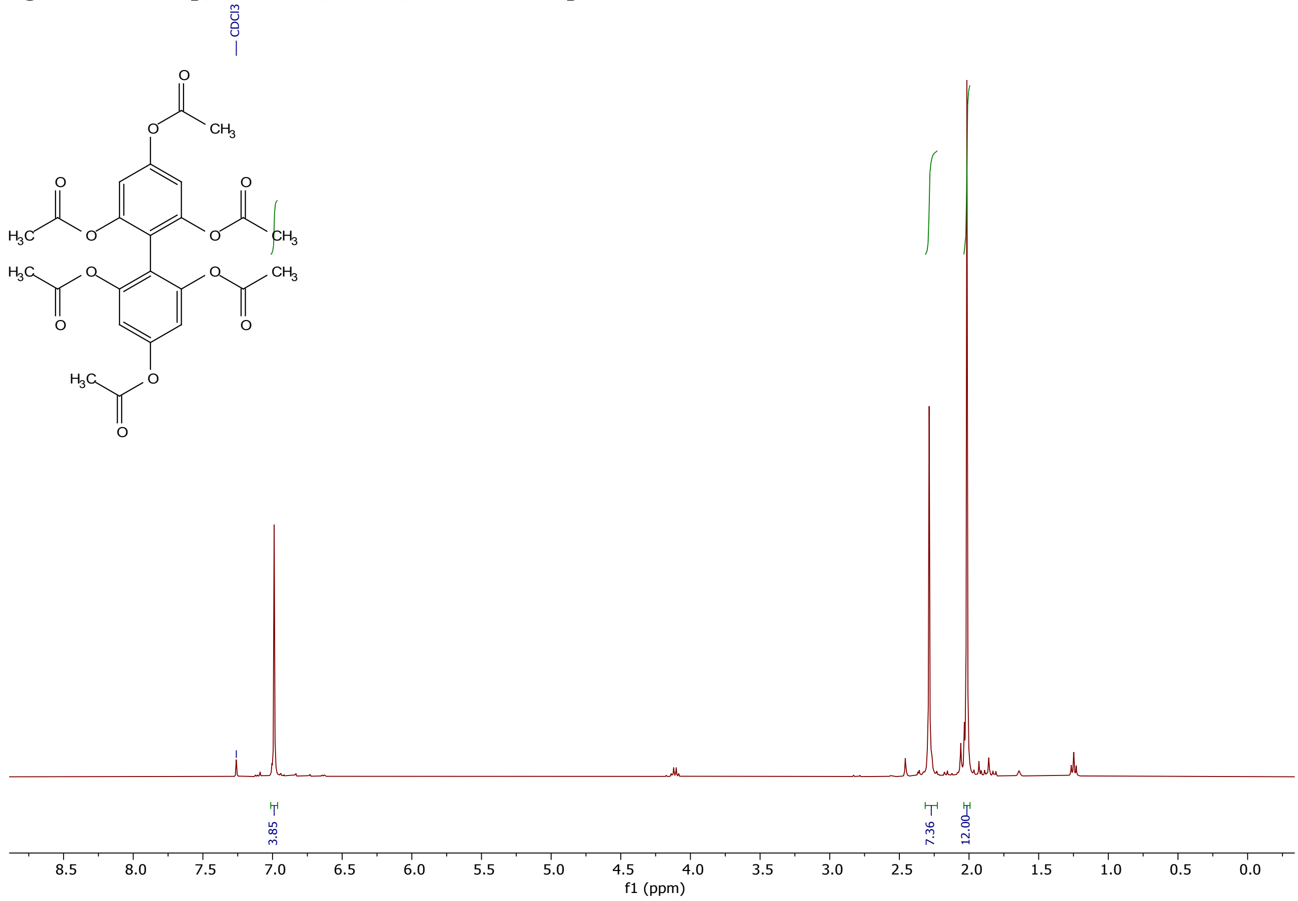

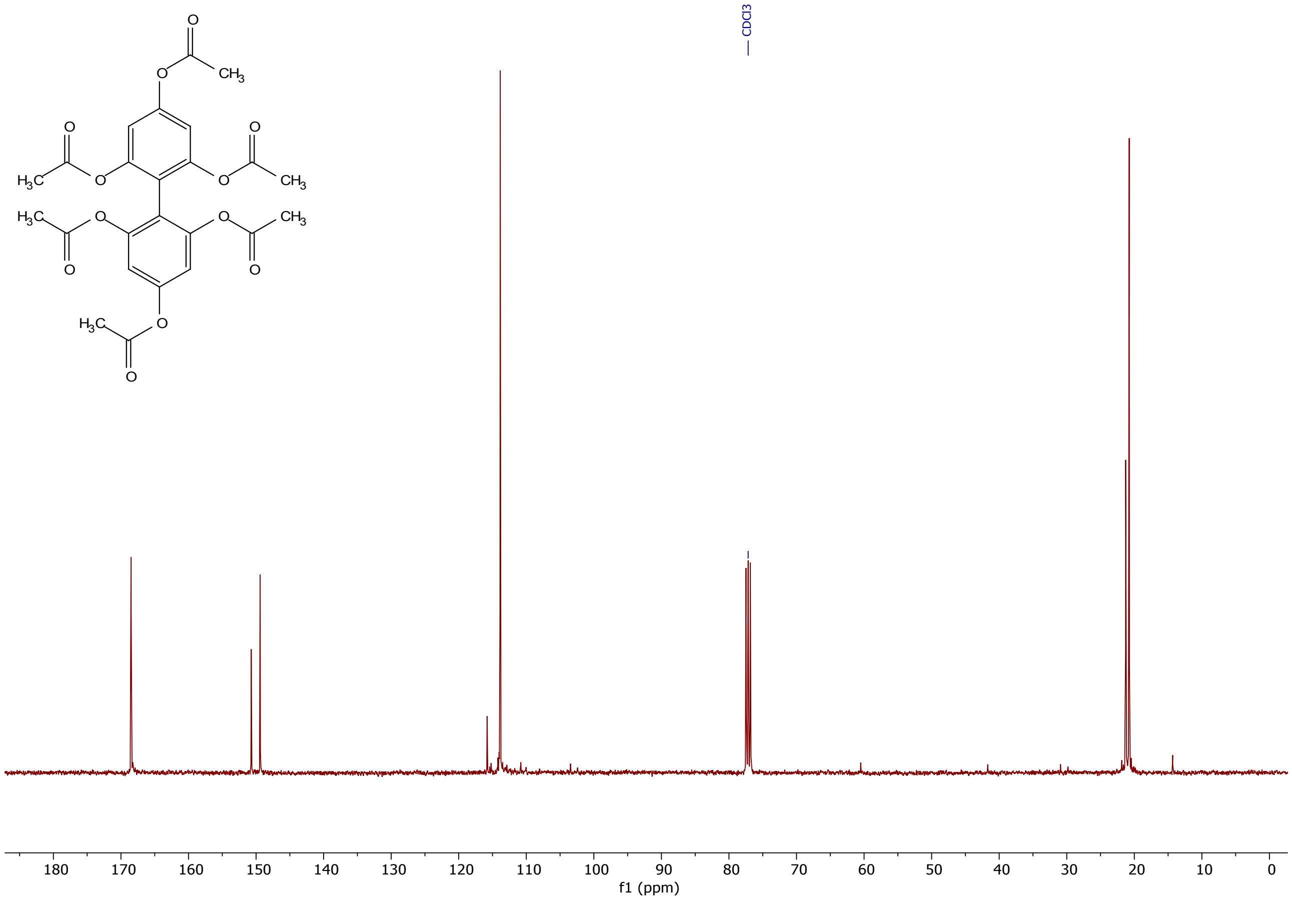

Figure S10. ^{1}H (top) and ^{13}C (bottom) NMR of compound 10.

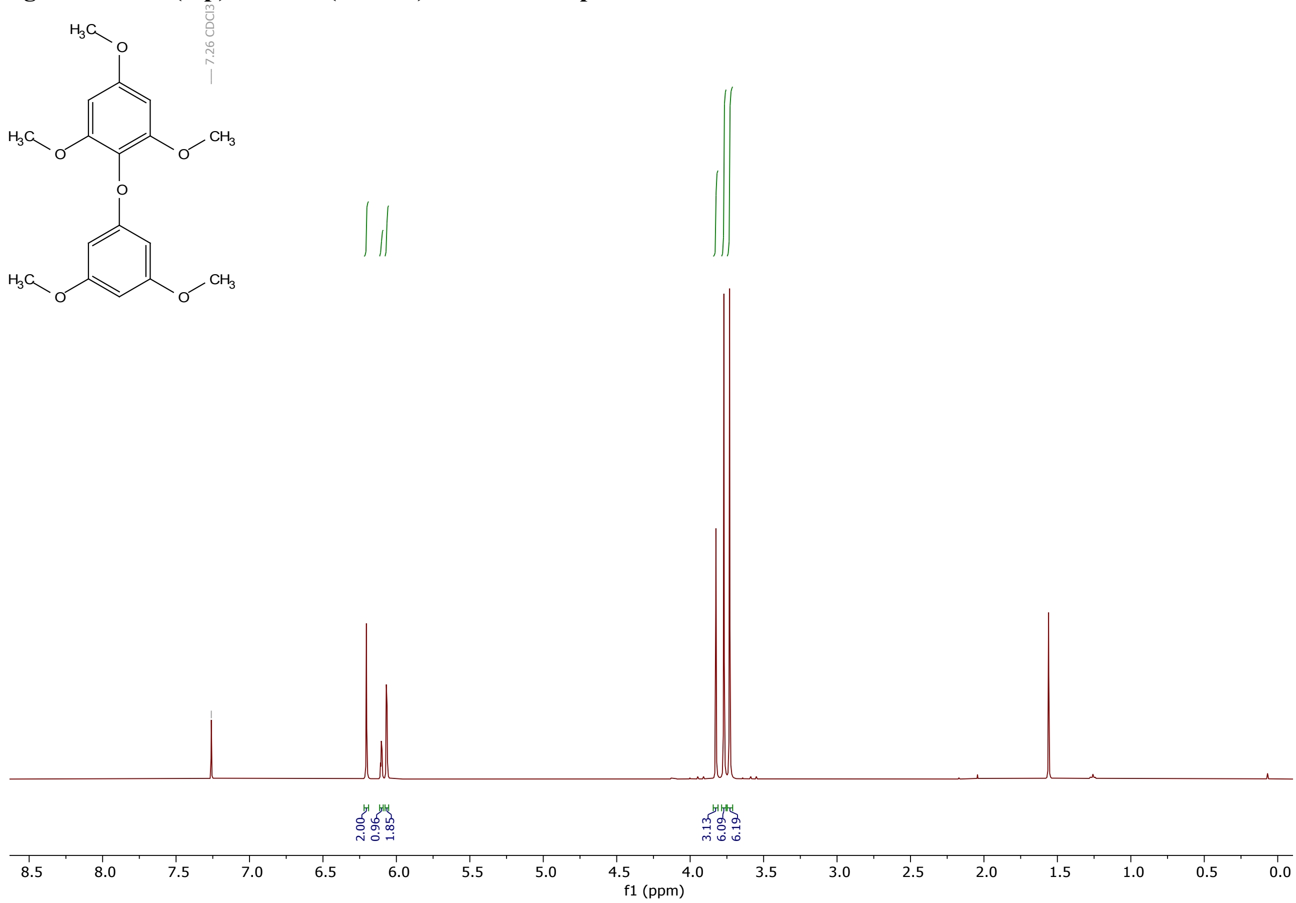

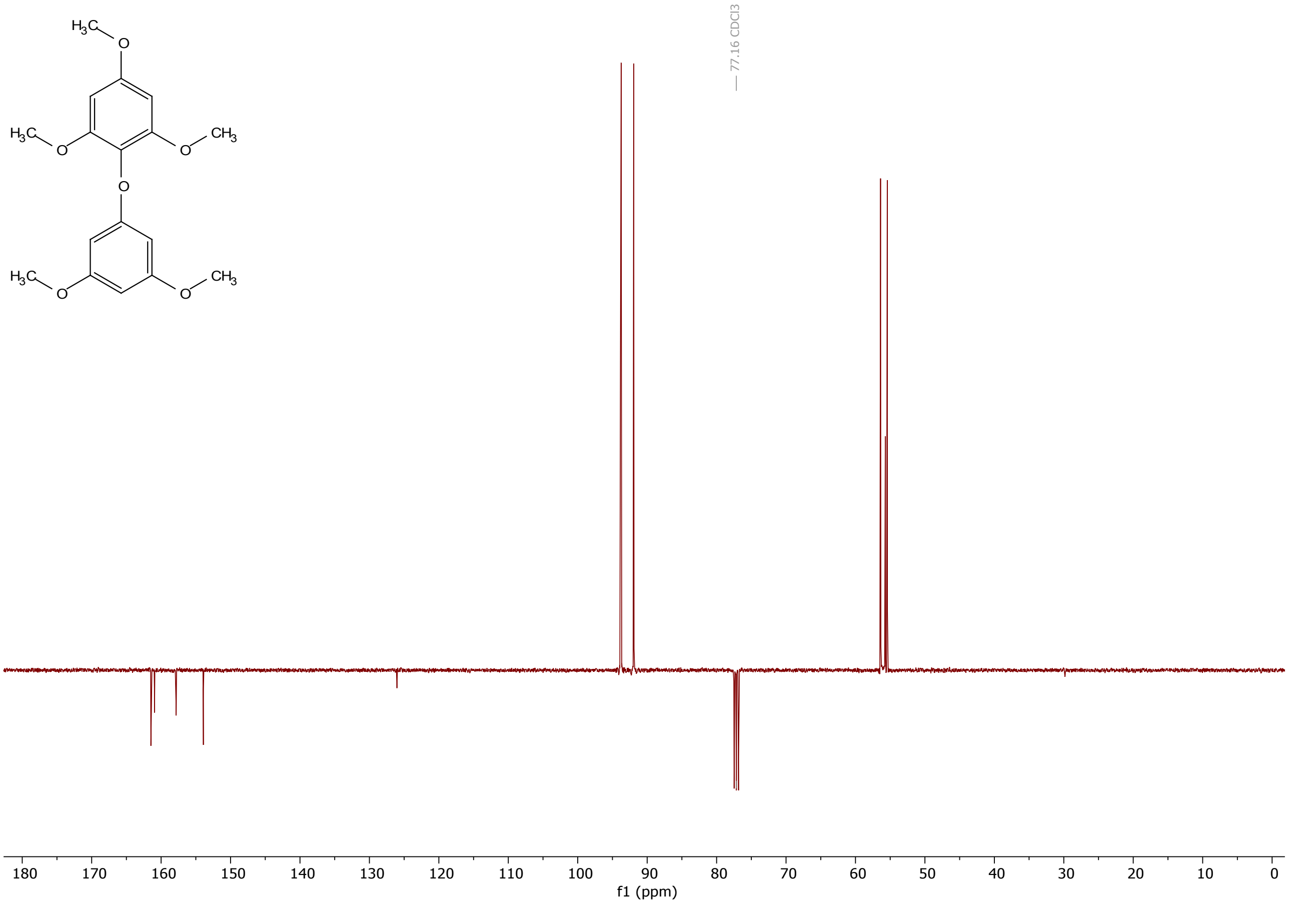

Figure S11. ^{1}H (top) and ^{13}C (bottom) NMR of compound 11.

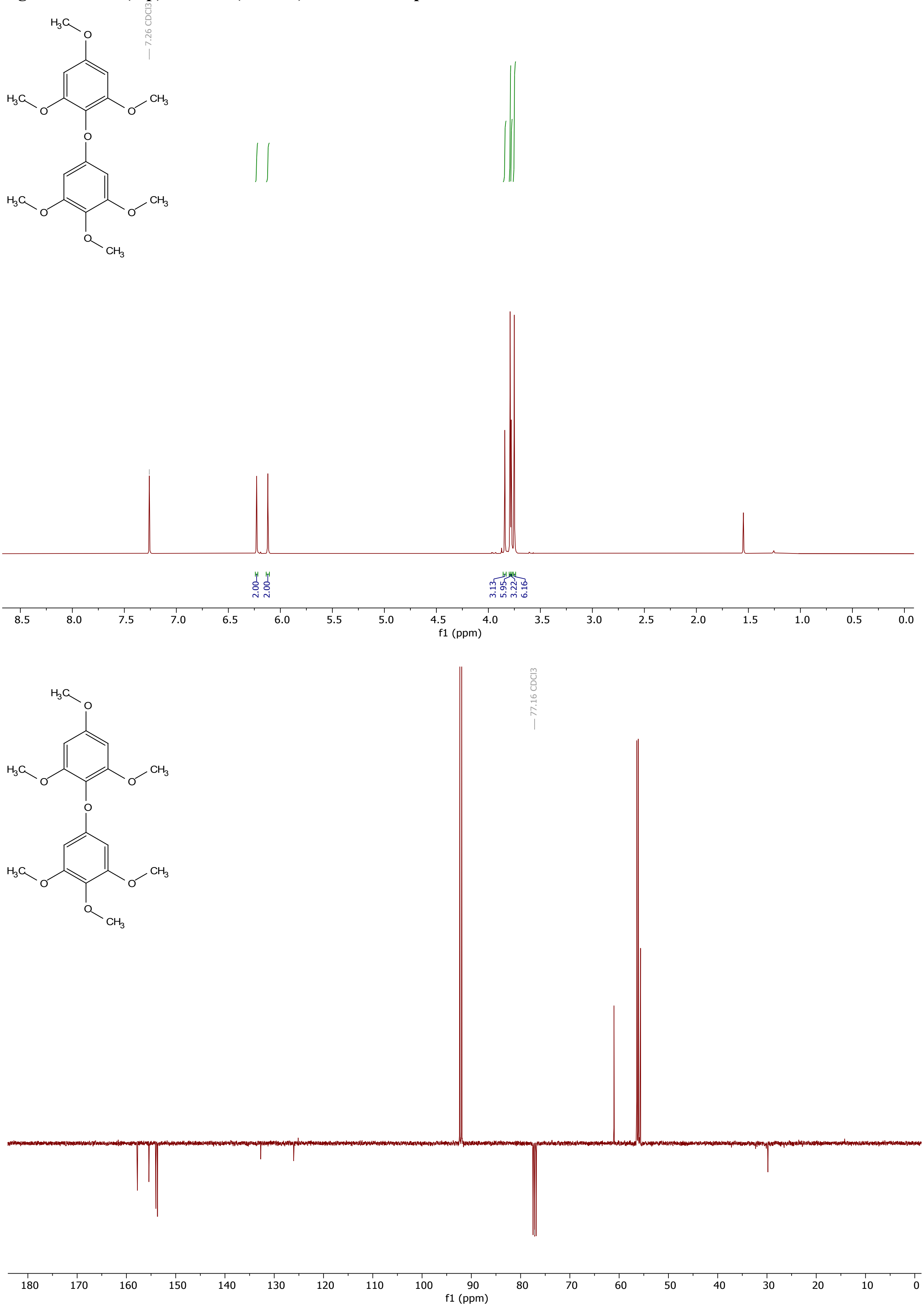

Figure S12. ^{1}H (top) and ^{13}C (bottom) NMR of compound 12.

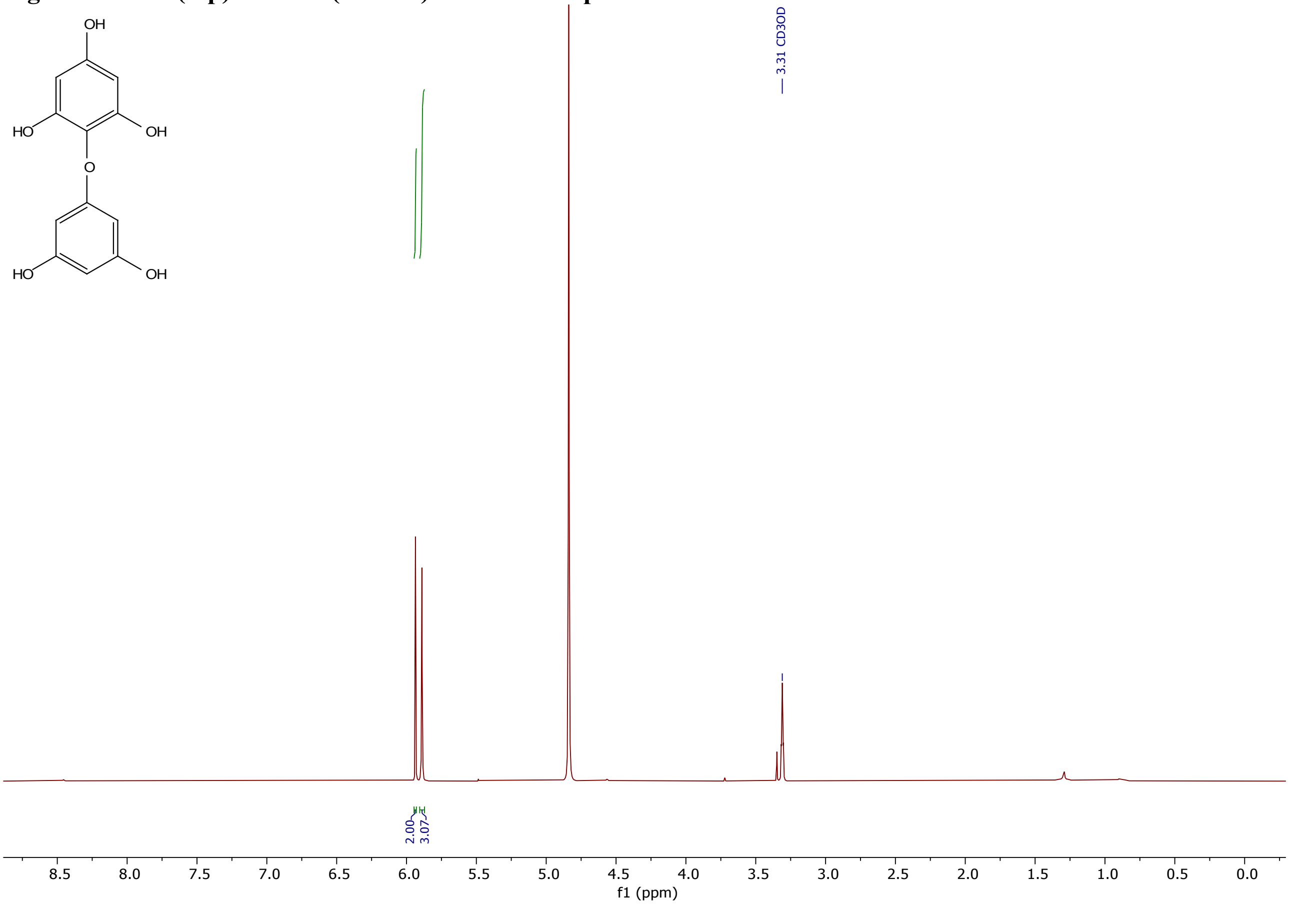

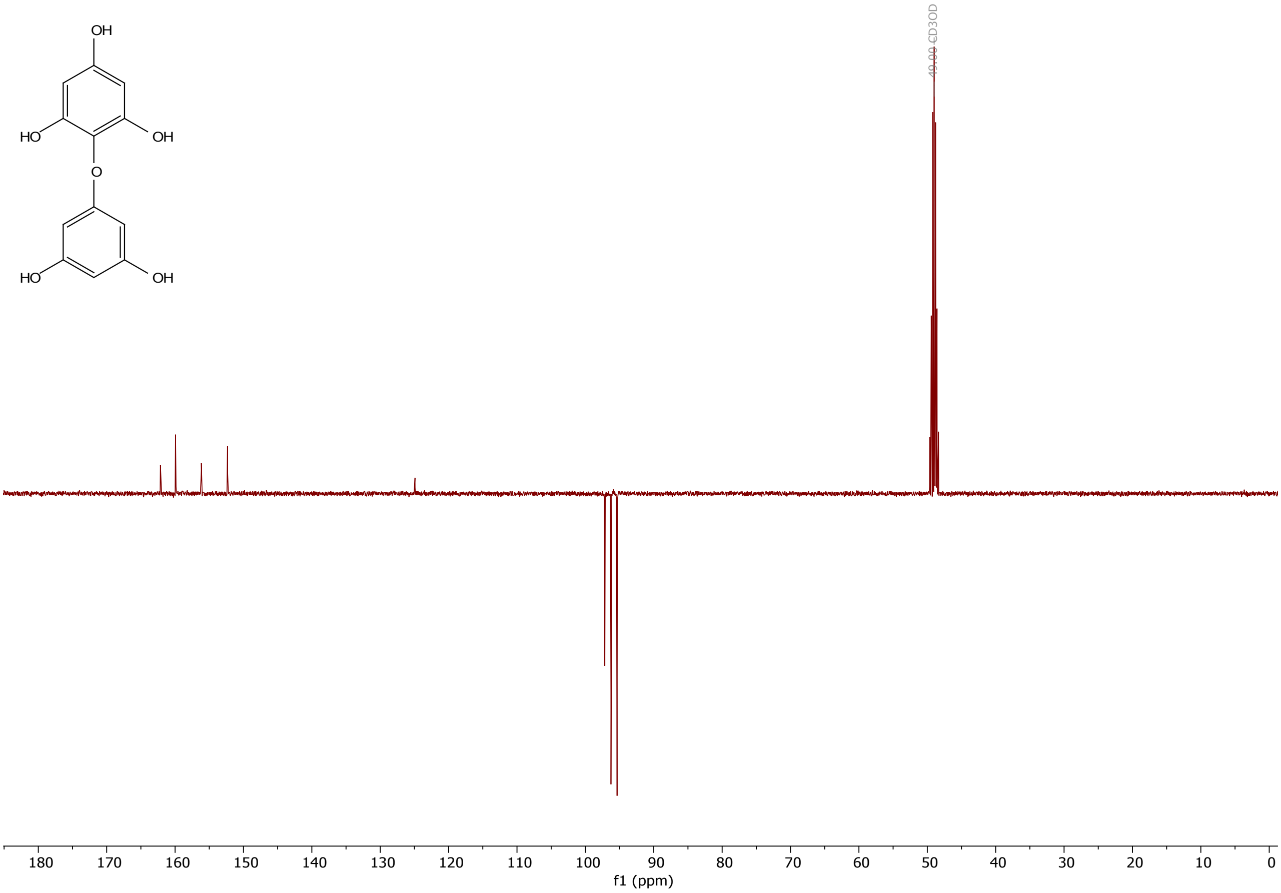

Figure S13. ^{1}H (top) and ^{13}C (bottom) NMR of compound 13.

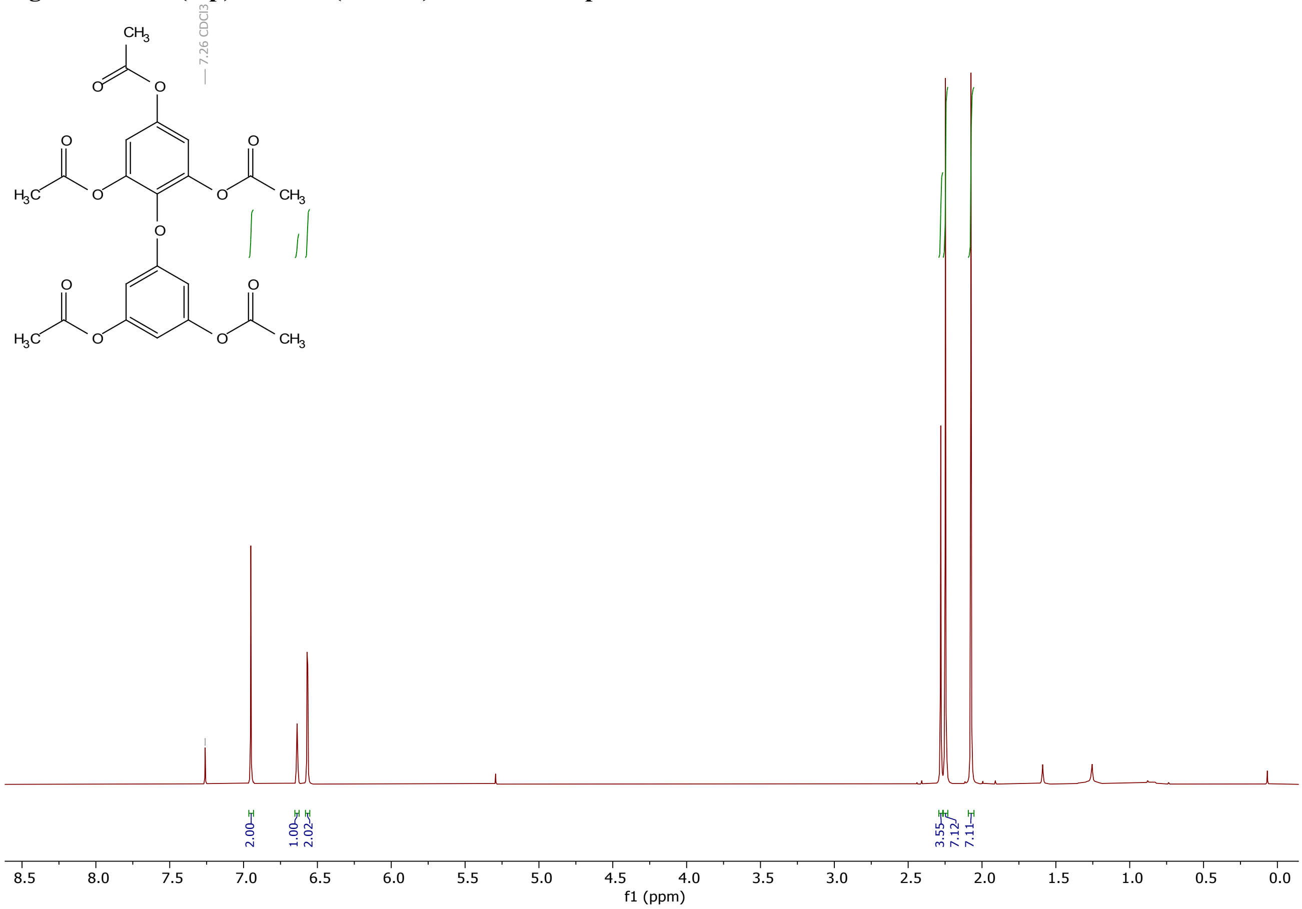

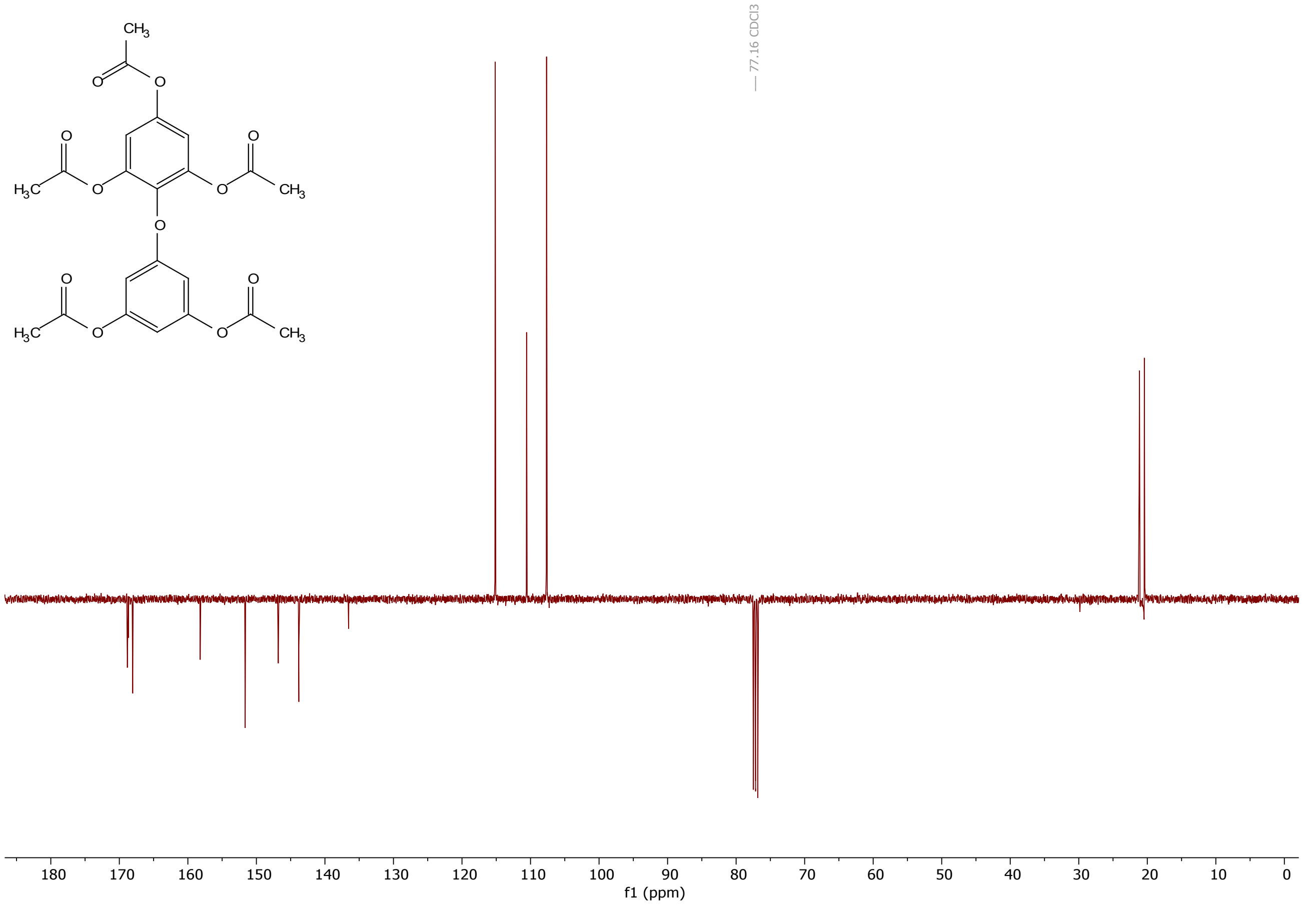

Figure S14. ^{1}H (top) and ^{13}C (bottom) NMR of compound 14.

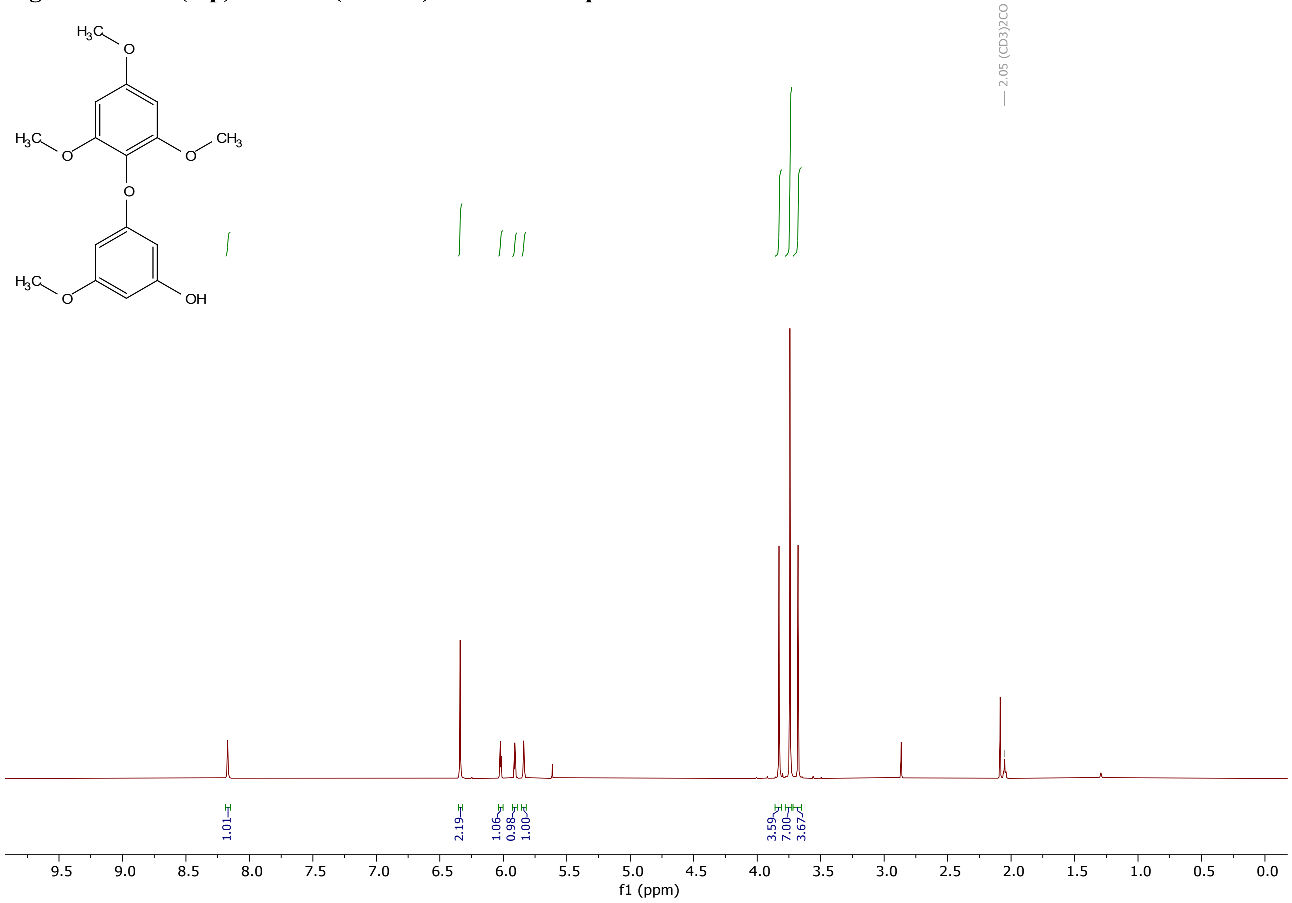

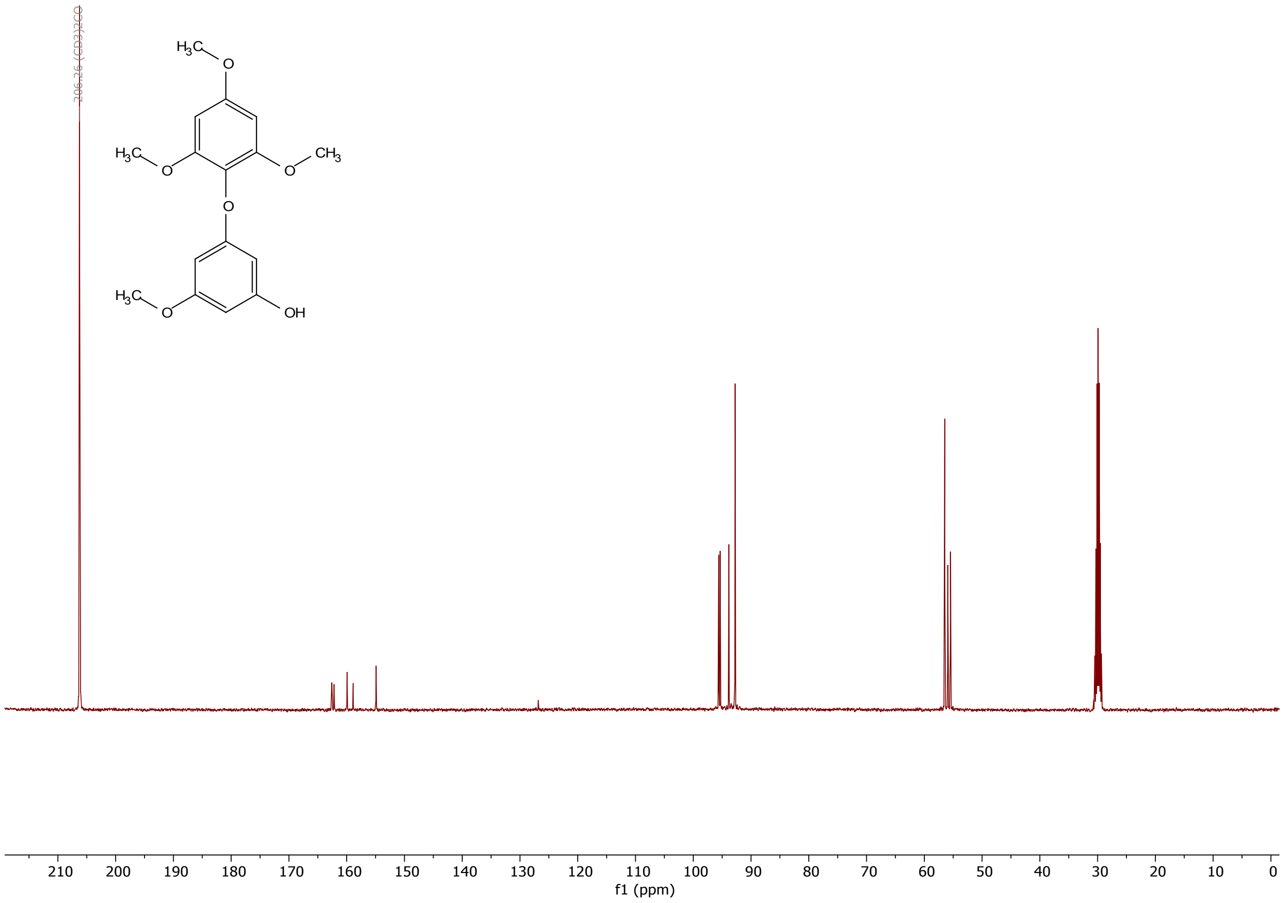

Figure S15. ^{1}H **(top) and** ^{13}C **(bottom) NMR of compound 15.**

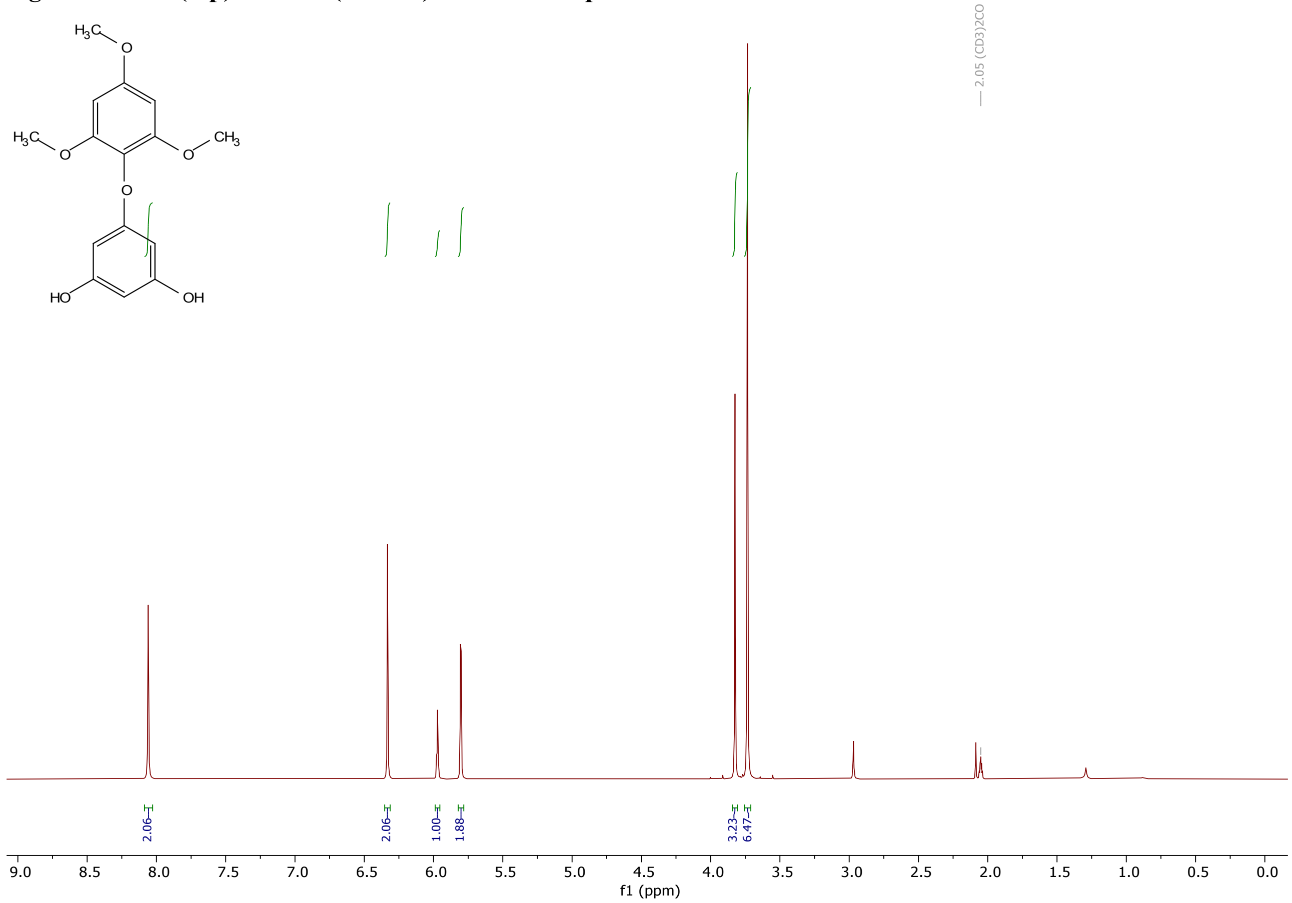

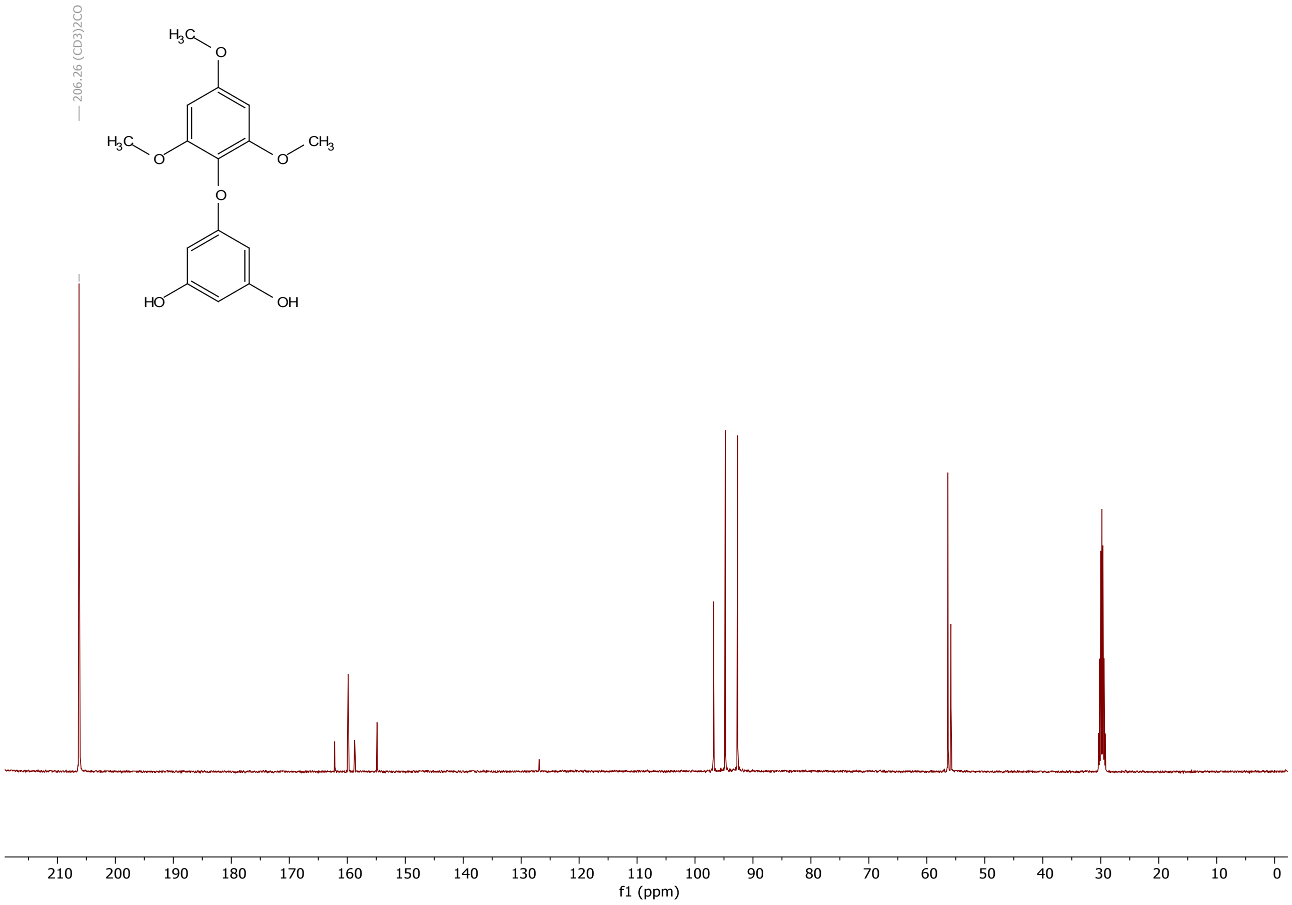

Figure S16. ^{1}H NMR of compound 20.

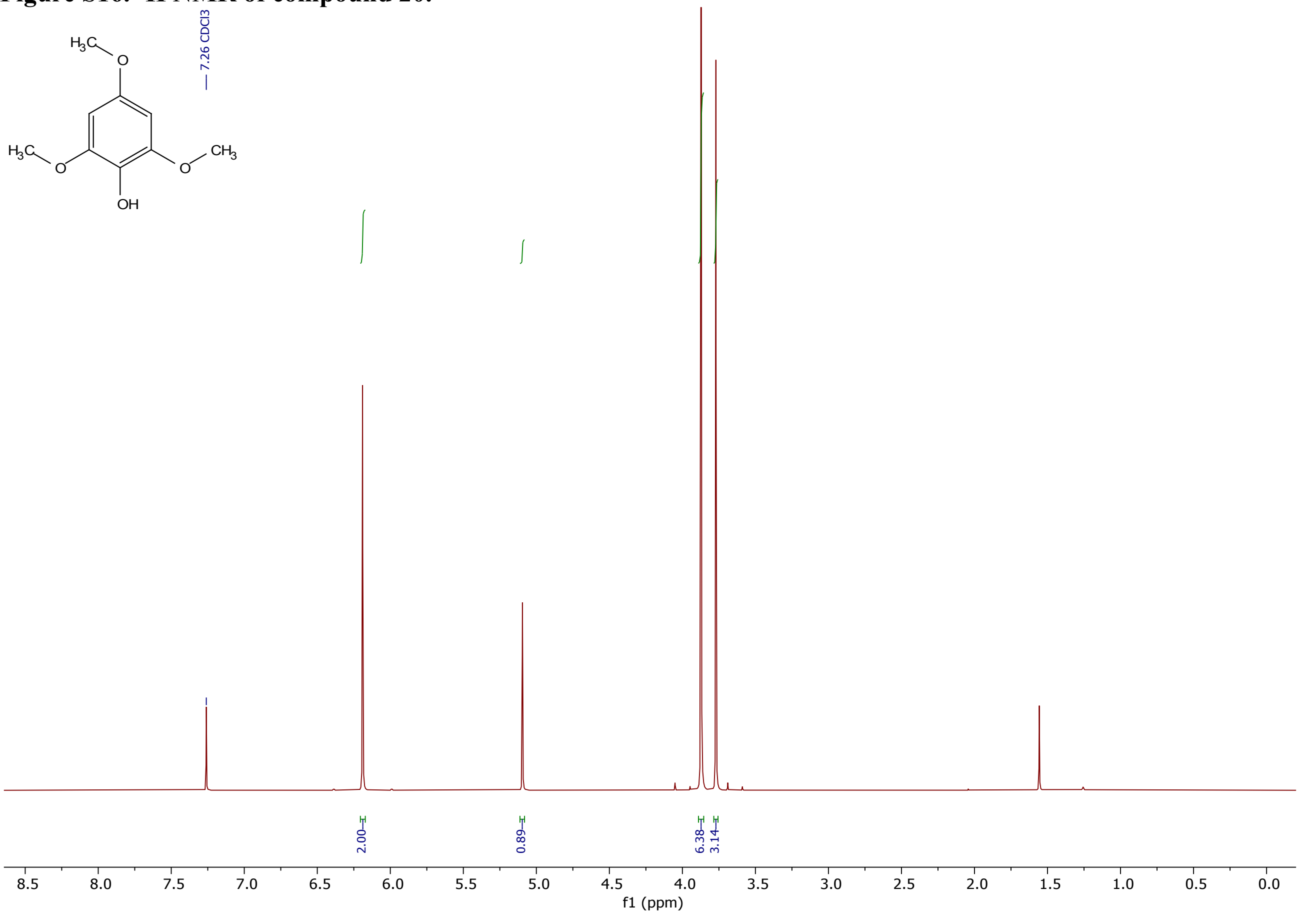

Figure S17. ^{1}H NMR of compound 23.

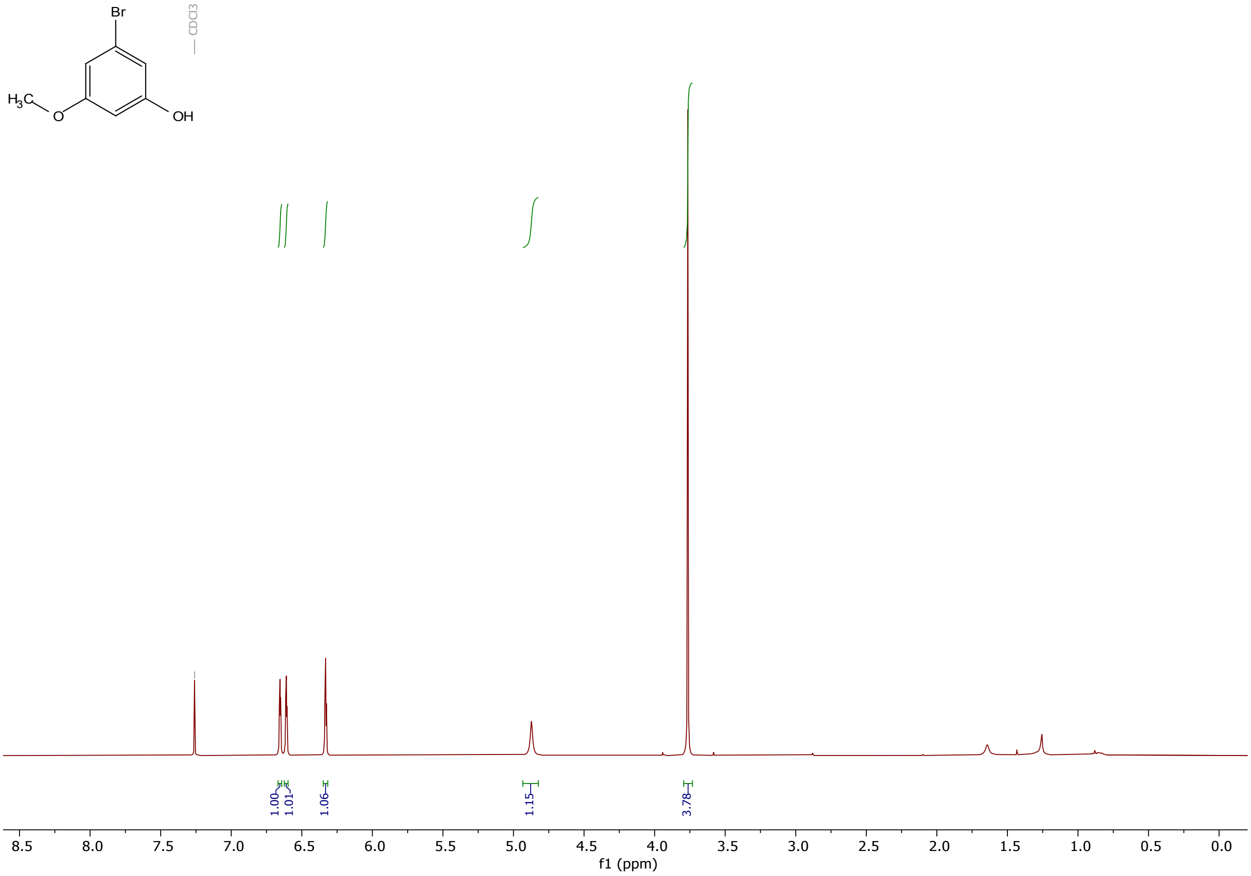

Figure S18. ^{1}H NMR of compound 24.

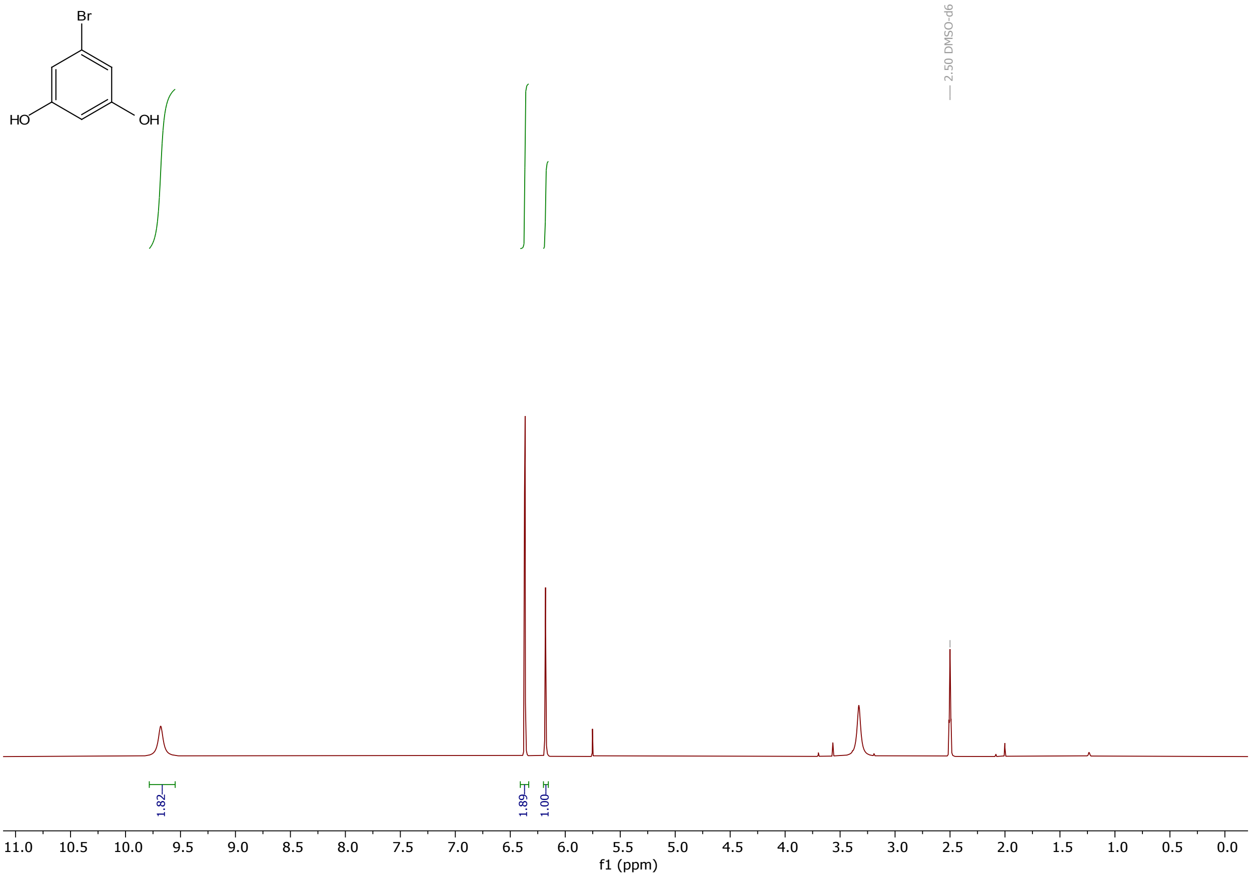

Figure S19. ^{1}H (top) and ^{13}C (bottom) NMR of compound 25.

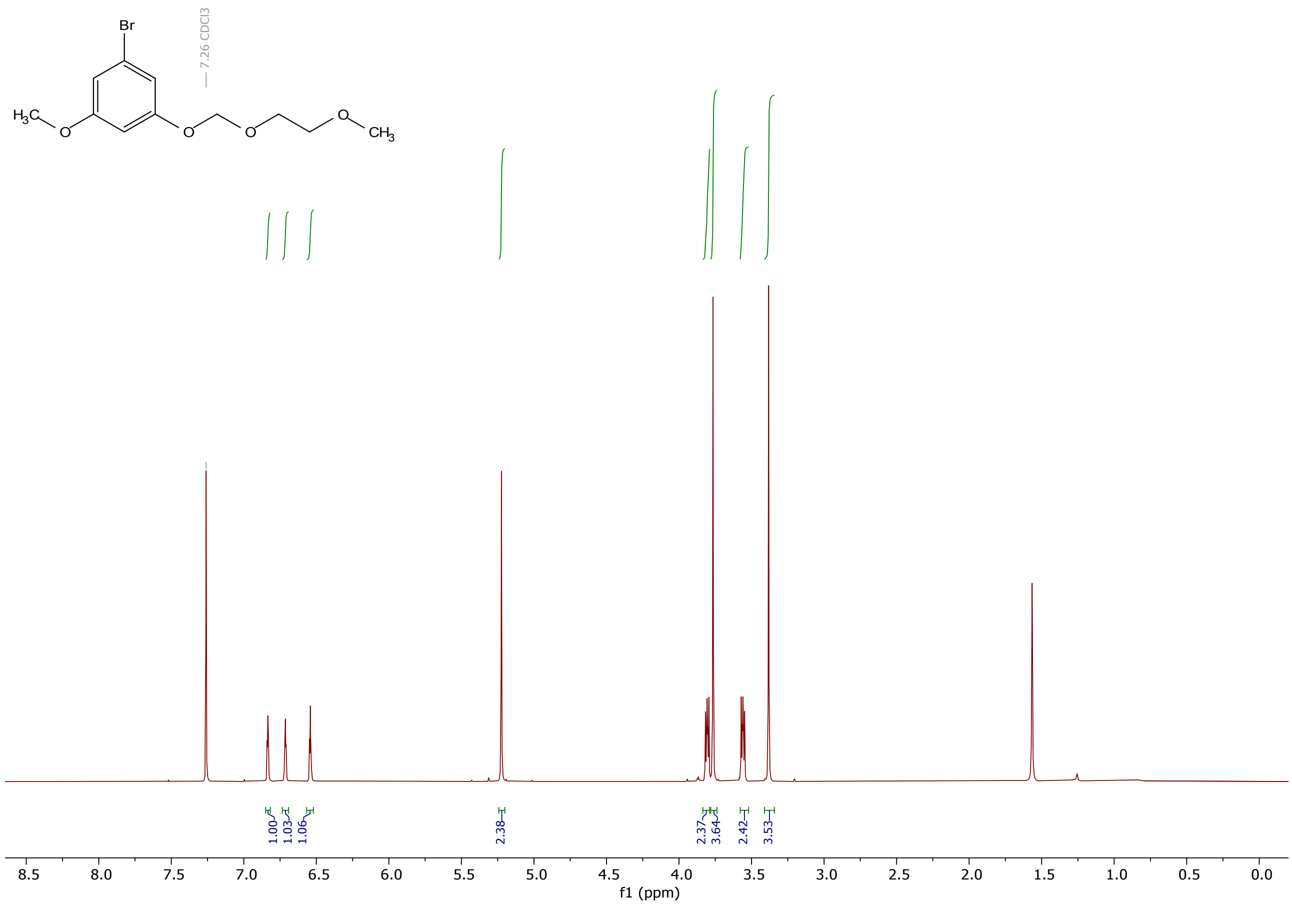

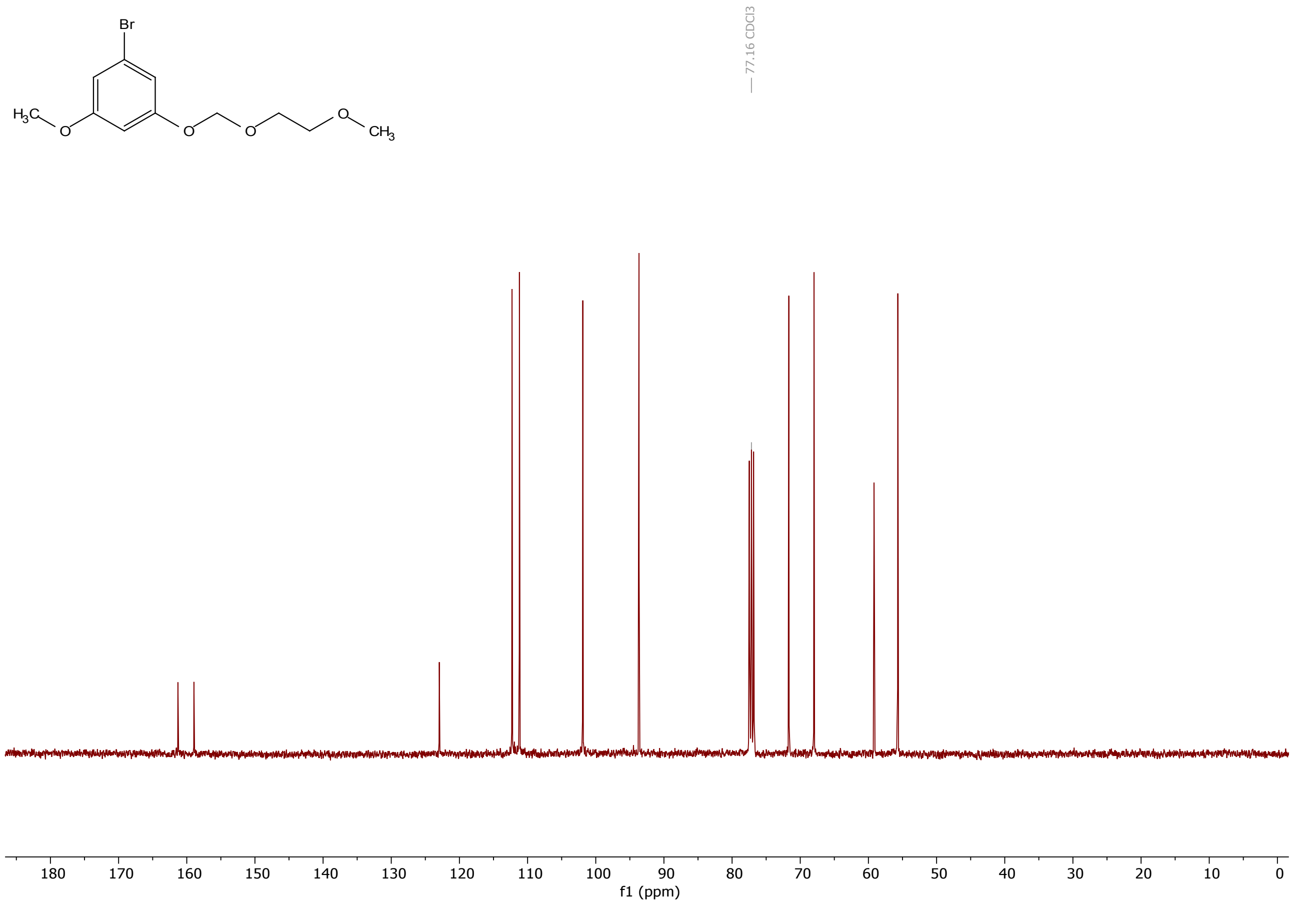

Figure S20. ^{1}H (top) and ^{13}C (bottom) NMR of compound 26.

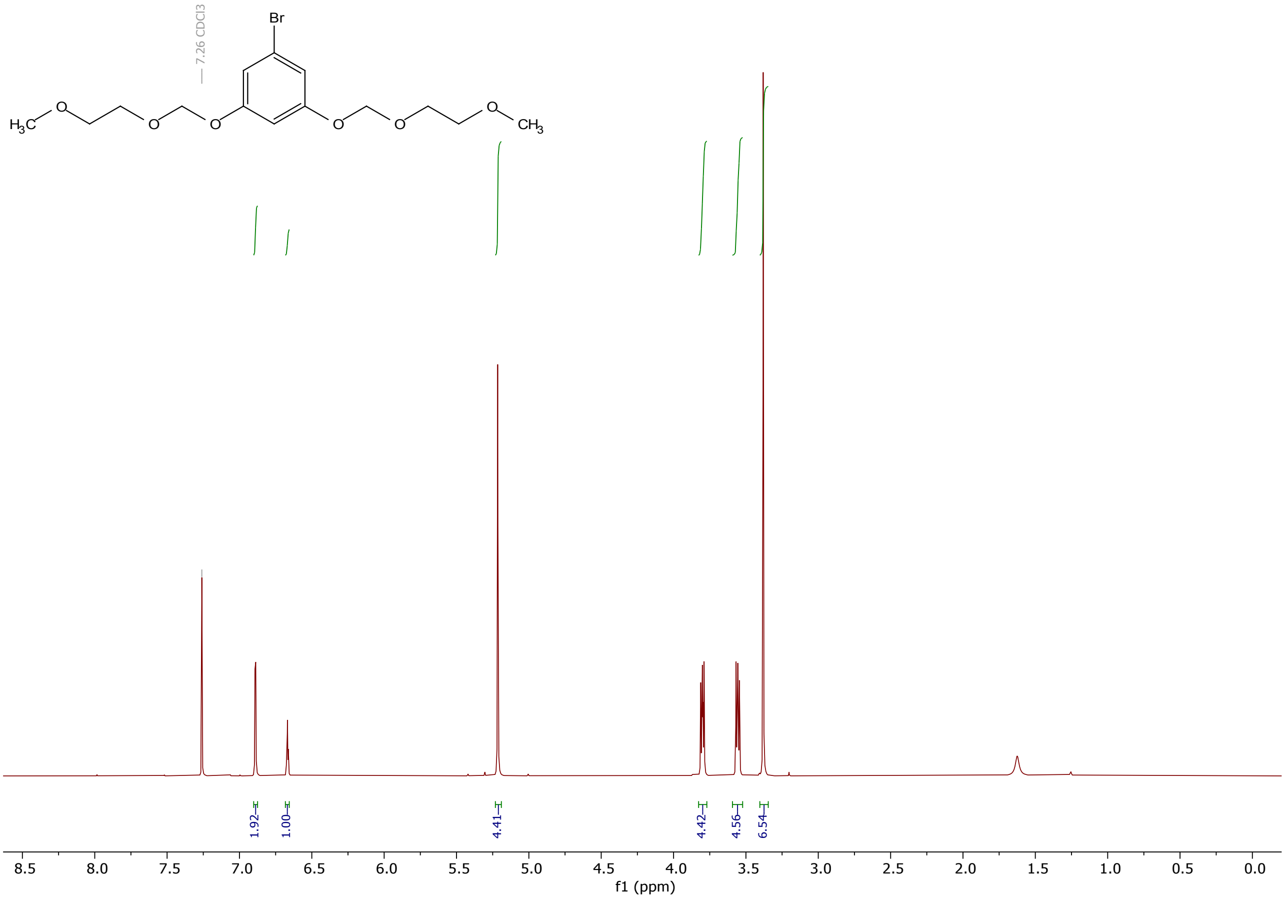

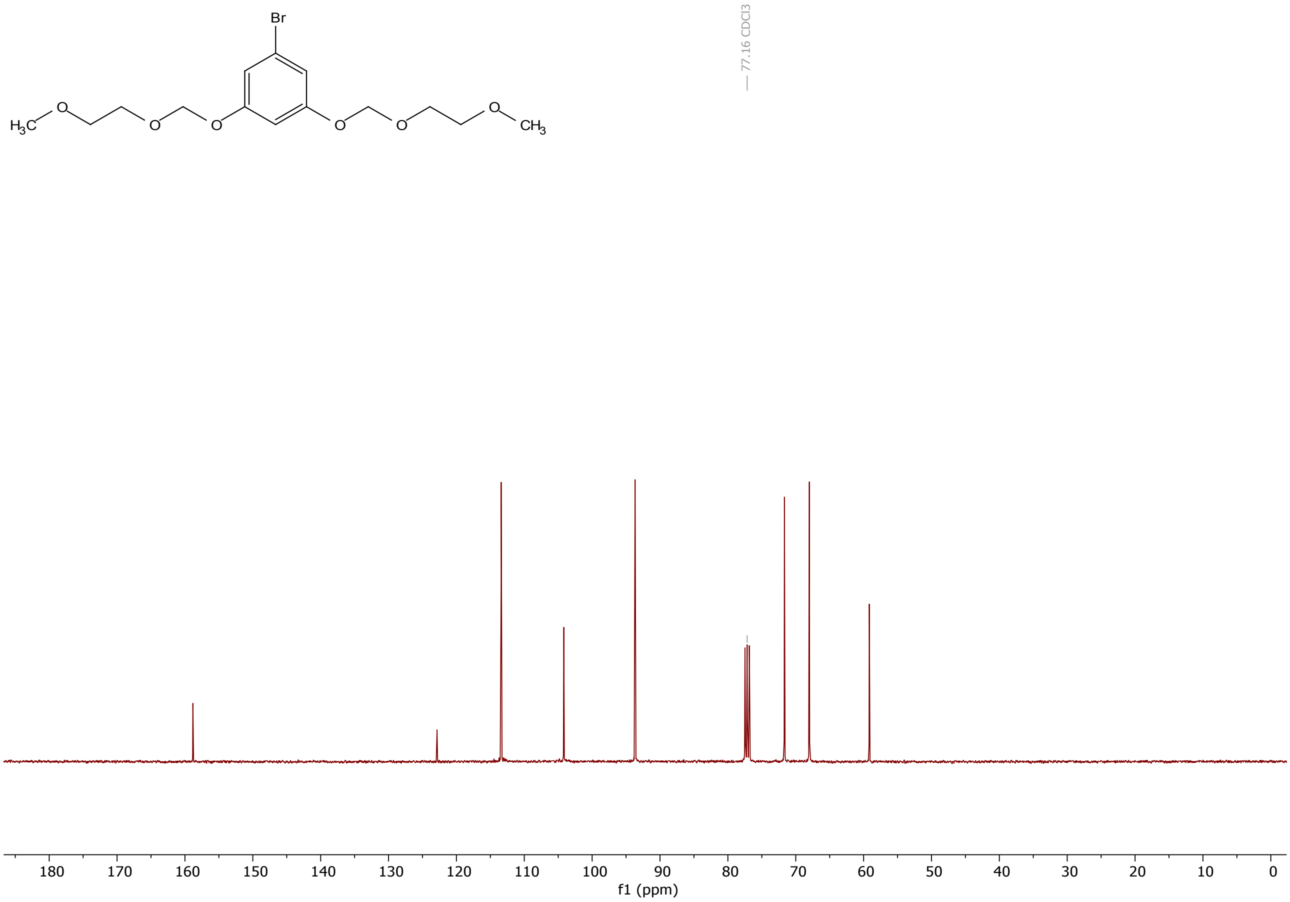

Figure S21. ^{1}H (top) and ^{13}C (bottom) NMR of compound 27.

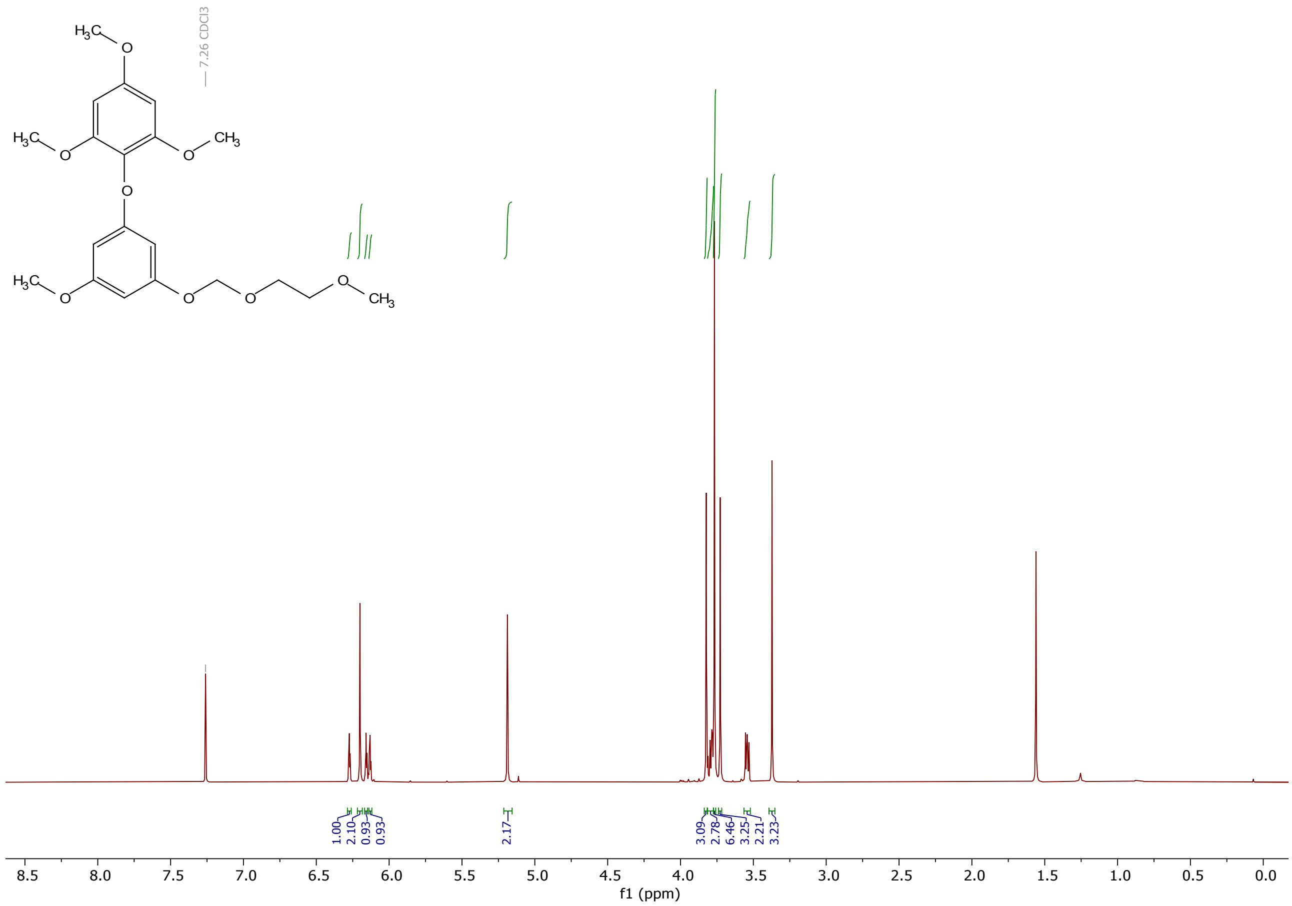

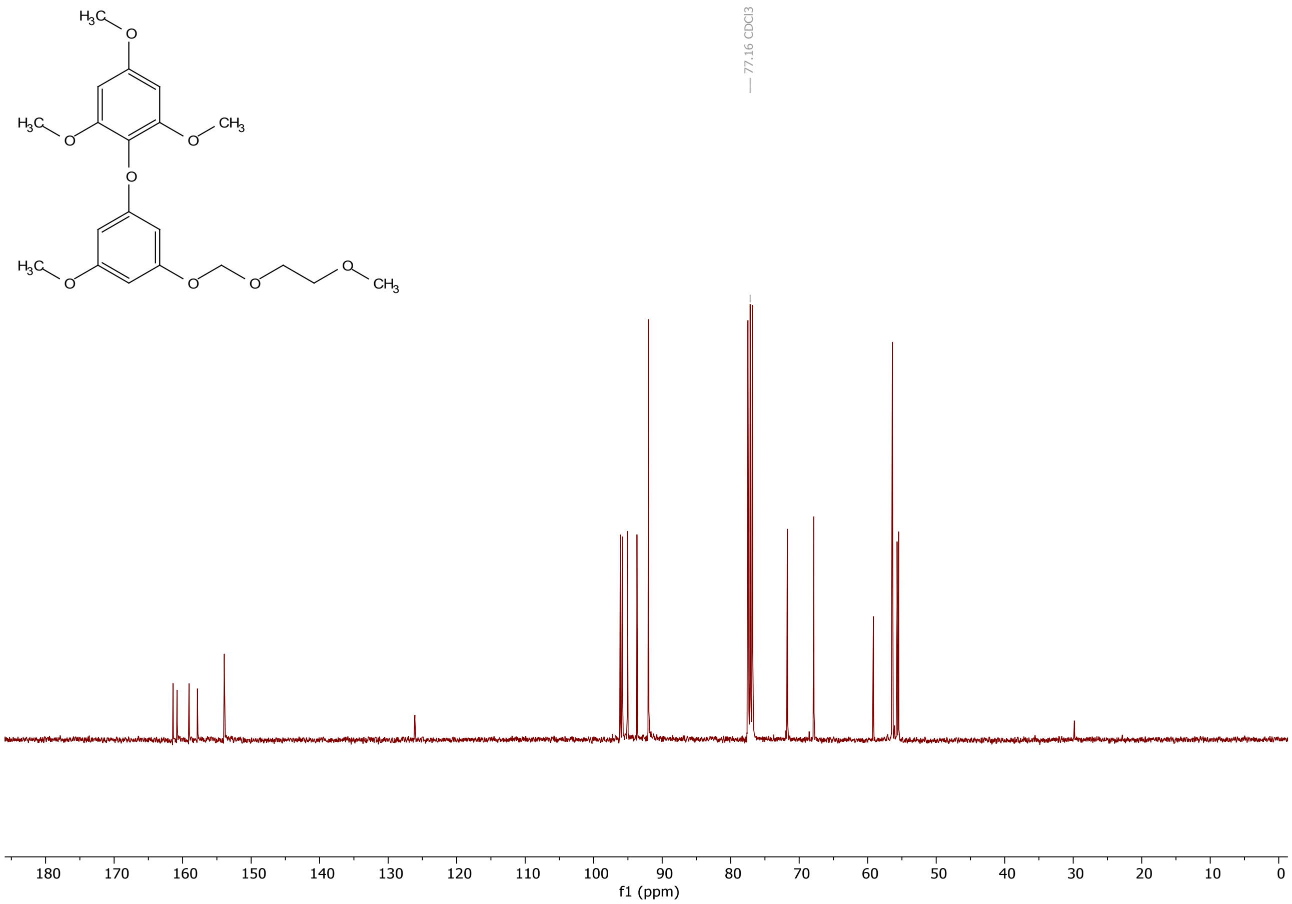

Figure S22. ^{1}H (top) and ^{13}C (bottom) NMR of compound 28.

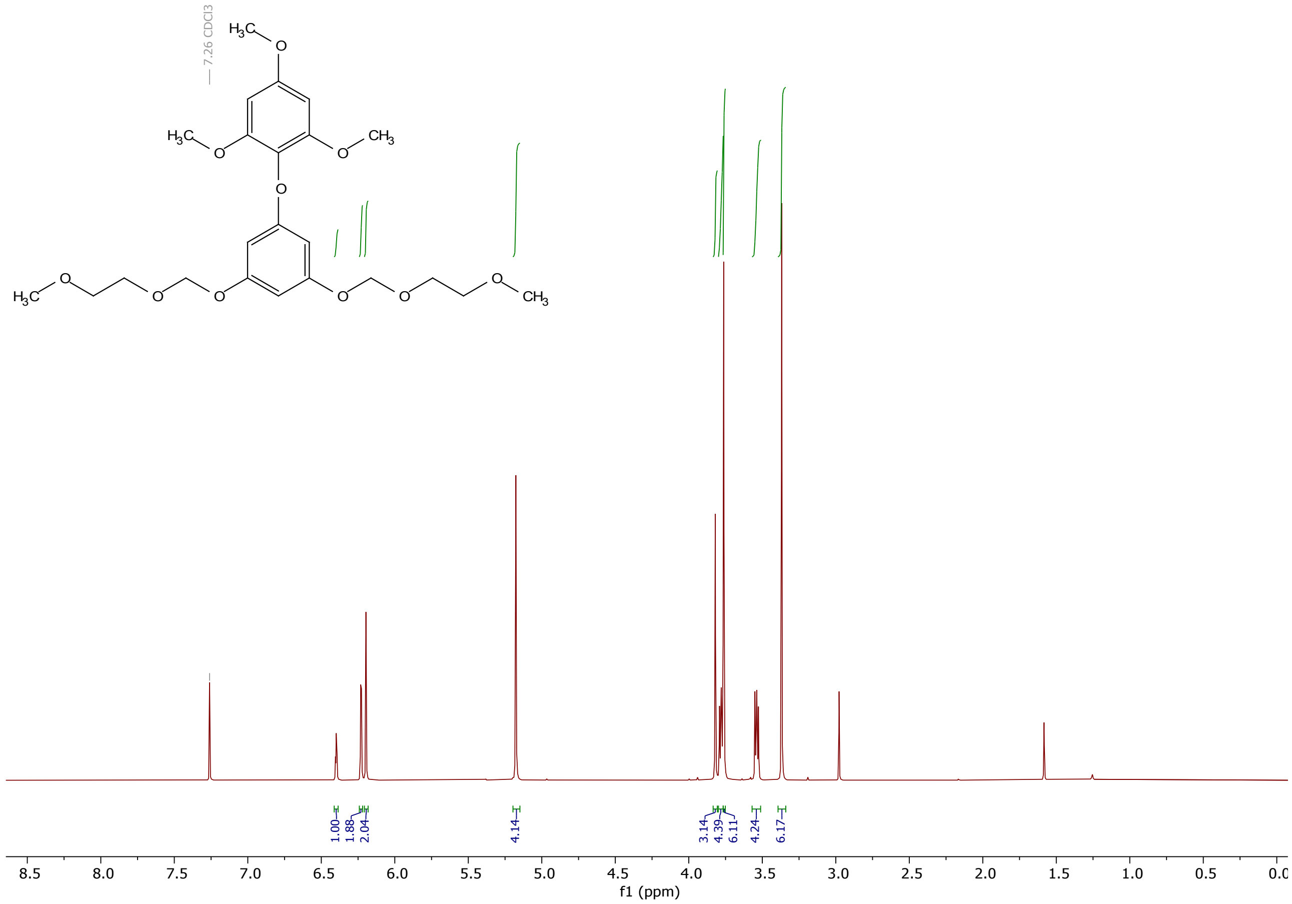

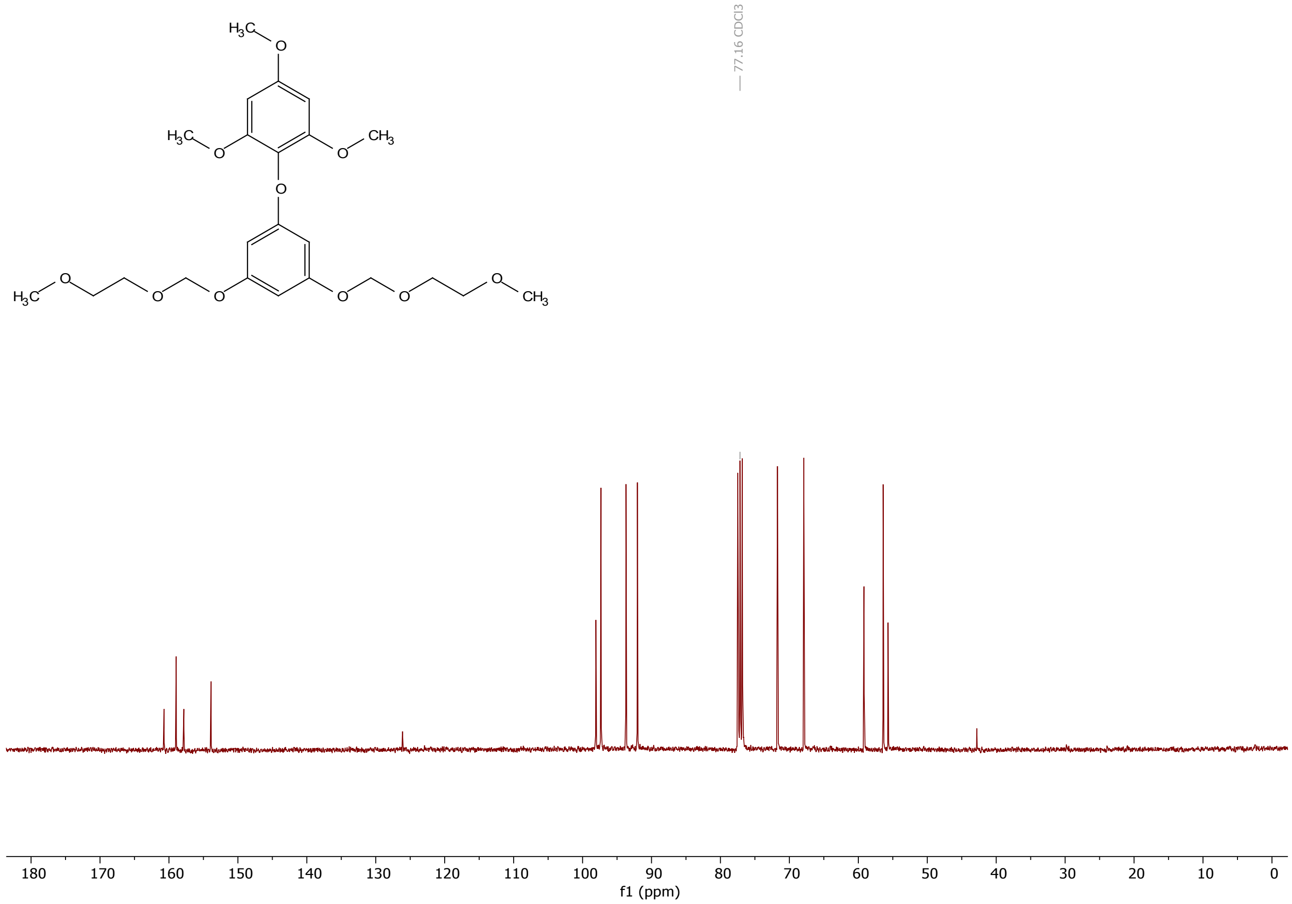

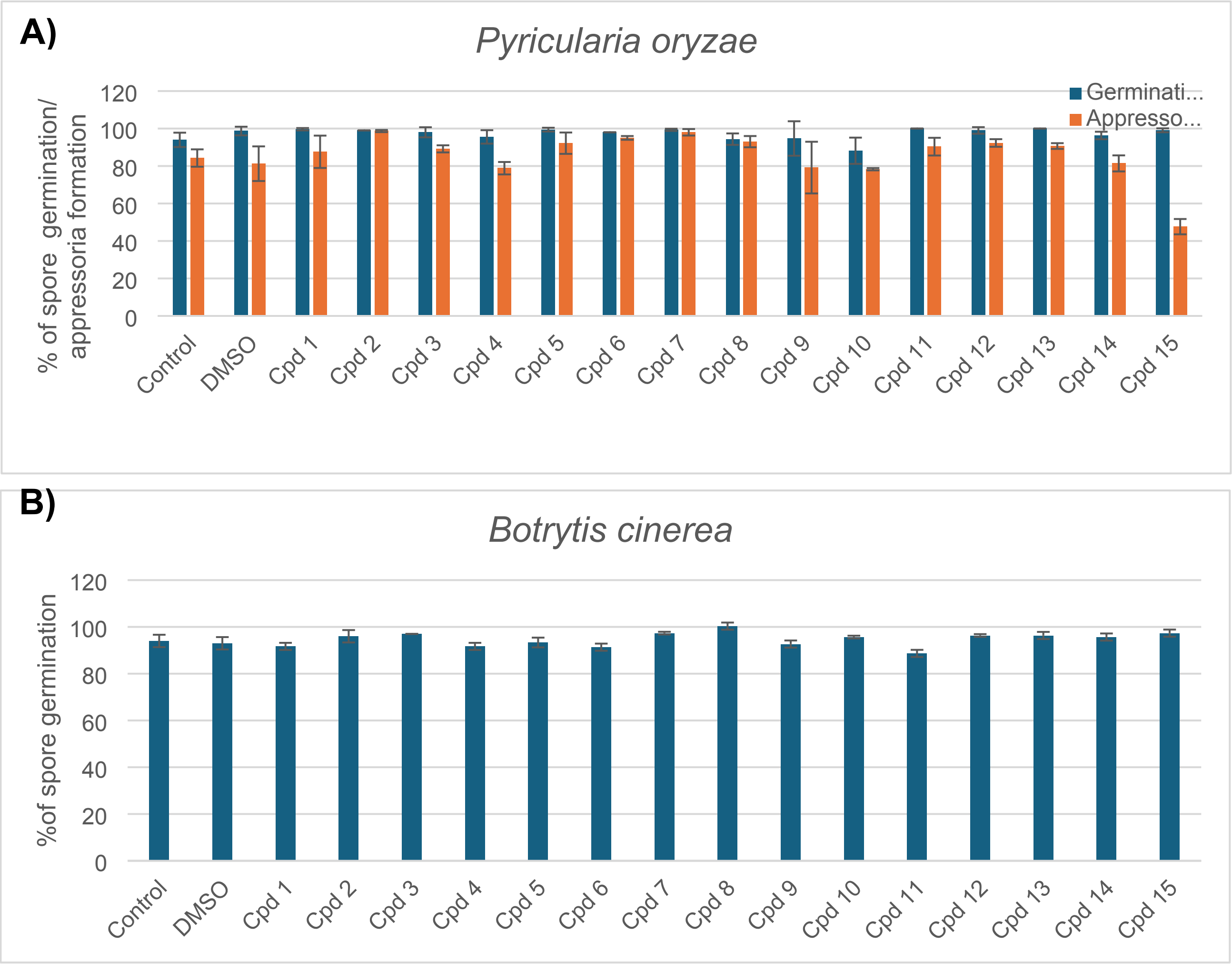

Figure S23: Spore germination and appressorium formation in *Pyricularia oryzae* (A) and in *Botrytis cinerea* (B) in control, 1% DMSO and tested compounds at 200 µM concentration.

Table S1: Predicted cLogP values for the synthesized compounds.

Compound	cLogP
**1**	-0.521
**2**	-0.262
**3**	0.392
**4**	0.132
**5**	-1.172
**6**	1.304
**7**	0.979
**8**	0.717
**9**	-2.116
**10**	3.135
**11**	2.354
**12**	0.405
**13**	0.285
**14**	2.549
**15**	1.963
**Phloroglucinol (PH1)**	0.141

Table S2: Minimum inhibition concentration (MIC) observed for the tested compounds **1-15** against Gram-positive *Staphylococcus aureus* (*SA*) and Gram-negative *Escherichia coli* (*EC*), *Salmonella enterica Enteritidis* (*SE*) and *Pseudomonas aeruginosa* (*PA*).

**Cpd**	***SA*** **MIC (µg/mL)**	***EC*** **MIC (µg/mL)**	***SE*** **MIC (µg/mL)**	***PA*** **MIC (µg/mL)**
**1**	128	128	128	128
**2**	256	128	128	128
**3**	128	128	128	128
**4**	128	256	256	128
**5**	128	128	128	128
**6**	256	128	256	128
**7**	256	256	128	128
**8**	256	128	128	128
**9**	256	256	256	128
**10**	256	128	128	128
**11**	128	128	128	128
**12**	256	128	128	128
**13**	256	128	128	128
**14**	256	128	128	128
**15**	256	128	128	128

SA *= Staphylococcus aureus;* ***EC*** *= Escherichia coli;* ***SE*** *= Salmonella enterica Enteritidis;* ***PA*** *= Pseudomonas aeruginosa*

Table S3: Growth inhibition data on PO-A252 strain.

Treatment	Strain	Growth	Growth average	%Inhibition	%I average
Control	PO-A252	4,8	4,77		
Control	PO-A252	4,75			
Control	PO-A252	4,75			
DMSO	PO-A252	4,5	4,43	-1,50	0,00
DMSO	PO-A252	4,4		0,75	
DMSO	PO-A252	4,4		0,75	
Cpd 1	PO-A252	4,45	4,48	-0,38	-1,13
Cpd 1	PO-A252	4,55		-2,63	
Cpd 1	PO-A252	4,45		-0,38	
Cpd 4	PO-A252	4,5	4,47	-1,50	-0,75
Cpd 4	PO-A252	4,5		-1,50	
Cpd 4	PO-A252	4,4		0,75	
Cpd 5	PO-A252	4,4	4,47	0,75	-0,75
Cpd 5	PO-A252	4,5		-1,50	
Cpd 5	PO-A252	4,5		-1,50	
Cpd 9	PO-A252	4,45	4,43	-0,38	0,00
Cpd 9	PO-A252	4,4		0,75	
Cpd 9	PO-A252	4,45		-0,38	
Treatment	Strain	Growth	Growth average	%Inhibition	%I average
Control	PO-A252	4,25	4,42		
Control	PO-A252	4,5			
Control	PO-A252	4,5			
DMSO	PO-A252	4,3	4,25	-1,18	0,00
DMSO	PO-A252	4,2		1,18	
DMSO	PO-A252	4,25		0,00	
Cpd 2	PO-A252	3,4	3,03	20,00	28,82
Cpd 2	PO-A252	2,65		37,65	
Cpd 6	PO-A252	4,3	4,27	-1,18	-0,39
Cpd 6	PO-A252	4,25		0,00	
Cpd 6	PO-A252	4,25		0,00	
Cpd 7	PO-A252	3,85	3,88	9,41	8,82
Cpd 7	PO-A252	3,9		8,24	
Cpd 8	PO-A252	4,15	4,08	2,35	3,92

Cpd 8	PO-A252	4,2		1,18	
Cpd 8	PO-A252	3,9		8,24	
Cpd 11	PO-A252	4	3,90	5,88	8,24
Cpd 11	PO-A252	3,7		12,94	
Cpd 11	PO-A252	4		5,88	
Treatment	Strain	Growth	Growth average	%Inhibition	%I average
Control	PO-A252	4,7	4,63		
Control	PO-A252	4,5			
Control	PO-A252	4,7			
DMSO	PO-A252	4,8	4,52	-6,27	0,00
DMSO	PO-A252	4,4		2,58	
DMSO	PO-A252	4,35		3,69	
Cpd 3	PO-A252	4	4,08	11,44	9,59
Cpd 3	PO-A252	4,1		9,23	
Cpd 3	PO-A252	4,15		8,12	
Cpd 10	PO-A252	3,65	3,73	19,19	17,34
Cpd 10	PO-A252	3,75		16,97	
Cpd 10	PO-A252	3,80		15,87	
Cpd 12	PO-A252	4,25	4,25	5,90	5,90
Cpd 12	PO-A252	4,25		5,90	
Cpd 12	PO-A252	4,25		5,90	
Cpd 13	PO-A252	4,15	4,23	8,12	6,27
Cpd 13	PO-A252	4,25		5,90	
Cpd 13	PO-A252	4,3		4,80	
Cpd 15	PO-A252	4,4	4,08	2,58	9,59
Cpd 15	PO-A252	3,9		13,65	
Cpd 15	PO-A252	3,95		12,55	
Treatment	Strain	Growth	Growth average	%Inhibition	%I average
Control	PO-A252	4,6	4,73		
Control	PO-A252	4,75			
Control	PO-A252	4,85			
DMSO	PO-A252	4,5	4,45	-1,12	0,00
DMSO	PO-A252	4,35		2,25	
DMSO	PO-A252	4,5		-1,12	
Cpd 14	PO-A252	3,60	3,58	19,10	19,48

Cpd 14	PO-A252	3,60		19,10	
Cpd 14	PO-A252	3,55		20,22	
Treatment	Strain	Growth	Growth average	%Inhibition	%I average
Control	PO-A252	3,9	3,90		
Control	PO-A252	3,9			
Control	PO-A252	3,9			
DMSO	PO-A252	3,8	3,82	0,44	0,00
DMSO	PO-A252	3,8		0,44	
DMSO	PO-A252	3,85		-0,87	
PH1	PO-A252	3,7	3,67	3,06	3,93
PH1	PO-A252	3,65		4,37	
PH1	PO-A252	3,65		4,37	

Table S4: Growth inhibition data on PO21-07 strain.

Treatment	Strain	Growth	Growth average	%Inhibition	%I average
Control	PO21-07	4,9	4,82		
Control	PO21-07	4,8			
Control	PO21-07	4,75			
DMSO	PO21-07	4,3	4,27	-0,78	0,00
DMSO	PO21-07	4,25		0,39	
DMSO	PO21-07	4,25		0,39	
Cpd 1	PO21-07	4,35	4,48	-1,95	-5,08
Cpd 1	PO21-07	4,55		-6,64	
Cpd 1	PO21-07	4,55		-6,64	
Cpd 4	PO21-07	4,1	4,05	3,91	5,08
Cpd 4	PO21-07	4,3		-0,78	
Cpd 4	PO21-07	3,75		12,11	
Cpd 5	PO21-07	3,85	4,08	9,77	4,30
Cpd 5	PO21-07	4,25		0,39	
Cpd 5	PO21-07	4,15		2,73	
Cpd 9	PO21-07	4	4,10	6,25	3,91
Cpd 9	PO21-07	4,1		3,91	
Cpd 9	PO21-07	4,2		1,56	
Treatment	Strain	Growth	Growth average	%Inhibition	%I average
Control	PO21-07	4,75	4,78		
Control	PO21-07	4,8			
Control	PO21-07	4,8			
DMSO	PO21-07	4,3	4,43	3,01	0,00
DMSO	PO21-07	4,5		-1,50	
DMSO	PO21-07	4,5		-1,50	
Cpd 2	PO21-07	4,05	4,22	8,65	4,89
Cpd 2	PO21-07	4,3		3,01	
Cpd 2	PO21-07	4,3		3,01	
Cpd 6	PO21-07	4,2	4,23	5,26	4,51
Cpd 6	PO21-07	4,2		5,26	
Cpd 6	PO21-07	4,3		3,01	
Cpd 7	PO21-07	3,8	3,78	14,29	14,66
Cpd 7	PO21-07	3,75		15,41	

Cpd 7	PO21-07	3,8		14,29	
Cpd 8	PO21-07	4,3	4,37	3,01	1,50
Cpd 8	PO21-07	4,3		3,01	
Cpd 8	PO21-07	4,5		-1,50	
Cpd 11	PO21-07	3,9	3,83	12,03	13,53
Cpd 11	PO21-07	3,75		15,41	
Cpd 11	PO21-07	3,85		13,16	
Treatment	Strain	Growth	Growth average	%Inhibition	%I average
Control	PO21-07	4,7	4,47		
Control	PO21-07	4,35			
Control	PO21-07	4,35			
DMSO	PO21-07	4,1	4,10	0,00	0,00
DMSO	PO21-07	4,2		-2,44	
DMSO	PO21-07	4		2,44	
Cpd 3	PO21-07	3,8	3,83	7,32	6,50
Cpd 3	PO21-07	3,85		6,10	
Cpd 3	PO21-07	3,85		6,10	
Cpd 10	PO21-07	3,25	3,28	20,73	19,92
Cpd 10	PO21-07	3,2		21,95	
Cpd 10	PO21-07	3,4		17,07	
Cpd 12	PO21-07	3,75	3,85	8,54	6,10
Cpd 12	PO21-07	4,05		1,22	
Cpd 12	PO21-07	3,75		8,54	
Cpd 13	PO21-07	4,05	4,08	1,22	0,41
Cpd 13	PO21-07	4,1		0,00	
Cpd 13	PO21-07	4,1		0,00	
Cpd 15	PO21-07	3,75	3,73	8,54	8,94
Cpd 15	PO21-07	3,6		12,20	
Cpd 15	PO21-07	3,85		6,10	
Treatment	Strain	Growth	Growth average	%Inhibition	%I average
Control	PO21-07	4,7	4,47		
Control	PO21-07	4,35			
Control	PO21-07	4,35			
DMSO	PO21-07	4,1	4,10	0,00	0,00
DMSO	PO21-07	4,2		-2,44	

DMSO	PO21-07	4		2,44	
Cpd 14	PO21-07	3,1	3,18	24,39	22,36
Cpd 14	PO21-07	3,3		19,51	
Cpd 14	PO21-07	3,15		23,17	
Treatment	Strain	Growth	Growth average	%Inhibition	%I average
Control	PO21-07	4,3	4,37		
Control	PO21-07	4,4			
Control	PO21-07	4,4			
DMSO	PO21-07	4	3,97	-0,84	0,00
DMSO	PO21-07	3,9		1,68	
DMSO	PO21-07	4		-0,84	
PH1	PO21-07	4	4,00	-0,84	-0,84
PH1	PO21-07	4		-0,84	
PH1	PO21-07	4		-0,84	

Table S5: Growth inhibition data on BC-2A-10 strain.

Treatment	Strain	Growth	Growth average	%Inhibition	%I average
Control	BC-2A-10	5,35	5,13		
Control	BC-2A-10	4,9			
Control	BC-2A-10	5,15			
DMSO	BC-2A-10	4,55	4,48	-1,68	0,00
DMSO	BC-2A-10	4,4		1,68	
Cpd 1	BC-2A-10	4,25	4,13	5,03	7,64
Cpd 1	BC-2A-10	4,05		9,50	
Cpd 1	BC-2A-10	4,1		8,38	
Cpd 4	BC-2A-10	4,45	4,47	0,56	0,19
Cpd 4	BC-2A-10	4,25		5,03	
Cpd 4	BC-2A-10	4,7		-5,03	
Cpd 5	BC-2A-10	3,7	4,23	17,32	5,40
Cpd 5	BC-2A-10	4,5		-0,56	
Cpd 5	BC-2A-10	4,5		-0,56	
Cpd 9	BC-2A-10	4,55	4,35	-1,68	2,79
Cpd 9	BC-2A-10	4,15		7,26	
Treatment	Strain	Growth	Growth average	%Inhibition	%I average
Control	BC-2A-10	4,95	3,32		
Control	BC-2A-10	2,7			
Control	BC-2A-10	2,3			
DMSO	BC-2A-10	3,2	3,60	11,11	0,00
DMSO	BC-2A-10	4		-11,11	
Cpd 2	BC-2A-10	2,35	2,90	34,72	19,44
Cpd 2	BC-2A-10	2,35		34,72	
Cpd 2	BC-2A-10	4		-11,11	
Cpd 6	BC-2A-10	3,55	3,73	1,39	-3,47
Cpd 6	BC-2A-10	3,9		-8,33	
Cpd 7	BC-2A-10	2,6	3,20	27,78	11,11
Cpd 7	BC-2A-10	3,8		-5,56	
Cpd 8	BC-2A-10	4,1	4,00	-13,89	-11,11
Cpd 8	BC-2A-10	3,9		-8,33	
Cpd 11	BC-2A-10	1,55	1,73	56,94	51,85
Cpd 11	BC-2A-10	2		44,44	

Cpd 11	BC-2A-10	1,65		54,17	
Treatment	Strain	Growth	Growth average	%Inhibition	%I average
Control	BC-2A-10	3,2	3,33		
Control	BC-2A-10	3,55			
Control	BC-2A-10	3,25			
DMSO	BC-2A-10	2,65	2,52	-5,30	0,00
DMSO	BC-2A-10	2,55		-1,32	
DMSO	BC-2A-10	2,35		6,62	
Cpd 3	BC-2A-10	3,65	3,70	-45,03	-47,02
Cpd 3	BC-2A-10	3,75		-49,01	
Cpd 10	BC-2A-10	2,05	2,15	18,54	14,57
Cpd 10	BC-2A-10	2,25		10,60	
Cpd 12	BC-2A-10	2,55	2,53	-1,32	-0,33
Cpd 12	BC-2A-10	2,5		0,66	
Cpd 13	BC-2A-10	2,45	2,43	2,65	3,31
Cpd 13	BC-2A-10	2,5		0,66	
Cpd 13	BC-2A-10	2,35		6,62	
Cpd 15	BC-2A-10	2,4	2,38	4,64	5,30
Cpd 15	BC-2A-10	2,5		0,66	
Cpd 15	BC-2A-10	2,25		10,60	
Treatment	Strain	Growth	Growth average	%Inhibition	%I average
Control	BC-2A-10	3,2	3,33		
Control	BC-2A-10	3,55			
Control	BC-2A-10	3,25			
DMSO	BC-2A-10	2,65	2,52	-5,30	0,00
DMSO	BC-2A-10	2,55		-1,32	
DMSO	BC-2A-10	2,35		6,62	
Cpd 14	BC-2A-10	2,45	2,45	2,65	2,65
Cpd 14	BC-2A-10	2,55		-1,32	
Cpd 14	BC-2A-10	2,35		6,62	
Treatment	Strain	Growth	Growth average	%Inhibition	%I average
Control	BC-2A-10	7,25	6,95		
Control	BC-2A-10	6,9			
Control	BC-2A-10	6,7			

DMSO	BC-2A-10	6,9	6,23	-10,70	0,00
DMSO	BC-2A-10	5,5		11,76	
DMSO	BC-2A-10	6,3		-1,07	
PH1	BC-2A-10	6,05	6,13	2,94	1,60
PH1	BC-2A-10	6,2		0,53	
PH1	BC-2A-10	6,15		1,34	

Table S6: Growth inhibition data on FC-UK strain.

Treatment	Strain	Growth	Growth average	%Inhibition	%I average
Control	FC-UK	4,7	4,57		
Control	FC-UK	4,45			
Control	FC-UK	4,55			
DMSO	FC-UK	5,4	5,42	0,31	0,00
DMSO	FC-UK	5,45		-0,62	
DMSO	FC-UK	5,4		0,31	
Cpd 1	FC-UK	5,6	5,52	-3,38	-1,85
Cpd 1	FC-UK	5,45		-0,62	
Cpd 1	FC-UK	5,5		-1,54	
Cpd 4	FC-UK	5,6	5,63	-3,38	-4,00
Cpd 4	FC-UK	5,5		-1,54	
Cpd 4	FC-UK	5,8		-7,08	
Cpd 5	FC-UK	5,4	5,47	0,31	-0,92
Cpd 5	FC-UK	5,35		1,23	
Cpd 5	FC-UK	5,65		-4,31	
Cpd 9	FC-UK	5,35	5,40	1,23	0,31
Cpd 9	FC-UK	5,4		0,31	
Cpd 9	FC-UK	5,45		-0,62	
Treatment	Strain	Growth	Growth average	%Inhibition	%I average
Control	FC-UK	5,05	5,47		
Control	FC-UK	5,55			
Control	FC-UK	5,8			
DMSO	FC-UK	5,7	5,65	-0,88	0,00
DMSO	FC-UK	5,3		6,19	
DMSO	FC-UK	5,95		-5,31	
Cpd 2	FC-UK	5,6	5,70	0,88	-0,88
Cpd 2	FC-UK	6		-6,19	
Cpd 2	FC-UK	5,5		2,65	
Cpd 6	FC-UK	5,45	5,42	3,54	4,13
Cpd 6	FC-UK	5,45		3,54	
Cpd 6	FC-UK	5,35		5,31	
Cpd 7	FC-UK	5,5	5,45	2,65	3,54
Cpd 7	FC-UK	5,5		2,65	

Cpd 7	FC-UK	5,35		5,31	
Cpd 8	FC-UK	5,4	5,35	4,42	5,31
Cpd 8	FC-UK	5,45		3,54	
Cpd 8	FC-UK	5,2		7,96	
Cpd 11	FC-UK	4,75	5,18	15,93	8,26
Cpd 11	FC-UK	5,4		4,42	
Cpd 11	FC-UK	5,4		4,42	
Treatment	Strain	Growth	Growth average	%Inhibition	%I average
Control	FC-UK	3,85	3,98		
Control	FC-UK	4,1			
Control	FC-UK	4			
DMSO	FC-UK	5,15	5,28	2,52	0,00
DMSO	FC-UK	5,65		-6,94	
DMSO	FC-UK	5,05		4,42	
Cpd 3	FC-UK	5,1	5,15	3,47	2,52
Cpd 3	FC-UK	5,05		4,42	
Cpd 3	FC-UK	5,3		-0,32	
Cpd 10	FC-UK	4,8	4,80	9,15	9,15
Cpd 10	FC-UK	4,75		10,09	
Cpd 10	FC-UK	4,85		8,20	
Cpd 12	FC-UK	5,25	5,25	0,63	0,63
Cpd 12	FC-UK	5,2		1,58	
Cpd 12	FC-UK	5,3		-0,32	
Cpd 13	FC-UK	5,3	5,13	-0,32	2,84
Cpd 13	FC-UK	5		5,36	
Cpd 13	FC-UK	5,1		3,47	
Cpd 15	FC-UK	5,15	5,12	2,52	3,15
Cpd 15	FC-UK	4,85		8,20	
Cpd 15	FC-UK	5,35		-1,26	
Treatment	Strain	Growth	Growth average	%Inhibition	%I average
Control	FC-UK	3,85	3,98		
Control	FC-UK	4,1			
Control	FC-UK	4			
DMSO	FC-UK	5,15	5,28	2,52	0,00
DMSO	FC-UK	5,65		-6,94	

DMSO	FC-UK	5,05		4,42	
Cpd 14	FC-UK	5	4,97	5,36	5,99
Cpd 14	FC-UK	5		5,36	
Cpd 14	FC-UK	4,9		7,26	
Treatment	Strain	Growth	Growth average	%Inhibition	%I average
Control	FC-UK	5,6	5,98		
Control	FC-UK	6,25			
Control	FC-UK	6,1			
DMSO	FC-UK	6,9	6,87	-0,49	0,00
DMSO	FC-UK	6,9		-0,49	
DMSO	FC-UK	6,8		0,97	
PH1	FC-UK	6,6	6,47	3,88	5,83
PH1	FC-UK	6,7		2,43	
PH1	FC-UK	6,1		11,17	
